# A Synthetic Phage-Peptide Conjugate as a Potent Antibacterial Agent for *Pseudomonas aeruginosa* Infections

**DOI:** 10.1021/acscentsci.5c00562

**Published:** 2025-07-31

**Authors:** Yanxi Yang, Shelby Vexler, Maria C. Jordan, Serena Abbondante, Dayeon Kang, Huan Peng, Michaela Marshall, Bita V. Naini, Saumya Jain, Yei-Chen Lai, Nasim Annabi, Kenneth P. Roos, Eric Pearlman, Irene A. Chen

**Affiliations:** † Department of Chemical and Biomolecular Engineering, 8783 University of California, Los Angeles, California 90095, United States; ‡ Department of Chemistry and Biochemistry, 8783 University of California, Los Angeles, California 90095, United States; § Department of Physiology, David Geffen School of Medicine, 8783 University of California, Los Angeles, California 90095, United States; ∥ Department of Ophthalmology, School of Medicine, 8788 University of California, Irvine, California 92697, United States; ⊥ Cellular Signaling Laboratory, International Research Center for Sensory Biology and Technology of MOST, Key Laboratory of Molecular Biophysics of MOE, College of Life Science and Technology, Huazhong University of Science and Technology, 430074 Wuhan, Hubei, China; # Department of Pathology & Laboratory Medicine, David Geffen School of Medicine, 8783 University of California, Los Angeles, California 90095, United States; ∇ Department of Chemistry, 34916 National Chung Hsing University, Taichung City 402, Taiwan

## Abstract

Antibiotic resistance among Gram-negative organisms is a major challenge. Some molecules, including antimicrobial peptides such as polymyxin B (PMB), are antibacterial but toxic due to low specificity, causing poor clinical utility. Drug delivery to bacterial cells using a biocompatible nanomaterial is a possible approach to securing such drugs. We engineered a nonlytic phage to recognize the lipopolysaccharide of Gram-negative bacteria and cross-linked thousands of peptides per virion, making “PMB-M13^αLPS^”. PMB-M13^αLPS^ reduced the minimum inhibitory concentration *in vitro* by ∼2 orders of magnitude across multiple pathogen strains. Immunocompetent mice with multidrug-resistant *P. aeruginosa* pneumonia or corneal infection were effectively treated by PMB-M13^αLPS^, which showed potency ∼2 orders of magnitude greater *in vivo* compared to that of PMB. PMB-M13^αLPS^ was well-tolerated, with no toxic effects. Conjugates of antimicrobial peptides and synthetic phages combine engineerable targeting with large payload capacity, improving potency and therapeutic index for otherwise toxic molecules.

## Graphical abstract

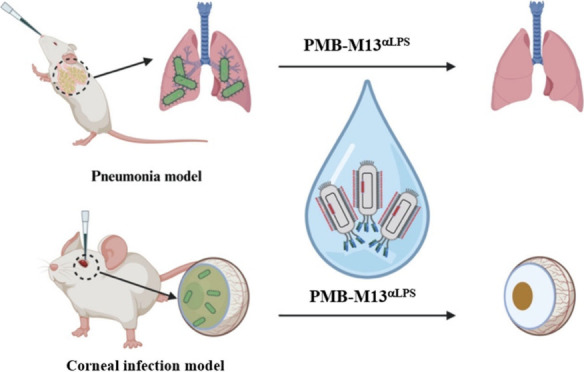

## Introduction

Antimicrobial resistance is a rising healthcare challenge across the globe.^1,2^ Gram-negative bacteria are a particular concern due to the presence of the outer membrane composed primarily of lipopolysaccharides (LPS), making them intrinsically resistant to some classes of antibiotics.^3,4^ Among others, the CDC considers the Gram-negative groups Enterobacterales (including *Escherichia coli* and *Klebsiella pneumoniae*), *Acinetobacter baumannii*, and *Pseudomonas aeruginosa* to be urgent or serious threats due to antibiotic resistance.^5^ At the same time, many antimicrobial peptides are highly effective against Gram-negative pathogens, but are rarely used due to their toxicity.^6,7^ Their toxicity is a consequence of a lack of specificity, resulting in undesired interactions with human cells. Conjugation of antimicrobial peptides to a biocompatible delivery vehicle is a potentially generalizable strategy to deliver high amounts of peptide specifically to bacteria, while minimizing toxicity to human cells. Here we demonstrate the efficacy of this concept by using an engineered variant of bacteriophage (phage) M13 to deliver polymyxin B to Gram-negative bacteria, effectively increasing antibiotic potency by up to 2 orders of magnitude both *in vitro* and *in vivo* .

Polymyxin B (PMB) is a positively charged cyclic peptide with a hydrophobic chain^8–10^ (Figure S1). The peptide binds in a low-affinity electrostatic interaction with the negatively charged bacterial surface, followed by the insertion of the hydrophobic chain into the outer membrane. This insertion displaces membrane-stabilizing cations and disrupts the outer membrane, causing membrane leakage and ultimately cell death.^7^ However, the concentrations required for efficacy overlap significantly with the toxic concentration range for mammalian cells, resulting in a low therapeutic index.^11^ PMB-induced nephrotoxicity is concentration-dependent and associated with the intracellular accumulation of PMB, resulting in acute tubular necrosis.^12,13^ While PMB was introduced clinically in the late 1950s, the high (up to 60%) rate of nephrotoxicity and other side effects caused it to be sidelined in the 1970s in favor of safer antibiotics.^14^ Despite its toxicity, PMB is now seen as a last-line therapy for carbapenem-resistant and multidrug-resistant Gram-negative bacteria, when frontline antibiotics (e.g., β-lactams, aminoglycosides, fluoroquinolones) fail.^7,12^ Nevertheless, nephrotoxicity leading to renal failure represents a difficult clinical trade-off. Thus, although PMB is highly antibacterial, its toxicity currently prevents its use as an earlier or more widespread treatment.^12^ Toxicity toward mammalian cells is a major concern affecting research and development of antimicrobial peptides (AMPs) generally, including the halt of a Phase III clinical trial of murepavidin due to nephrotoxicity.^15^

A strategy to overcome toxic side effects is to deliver the drug specifically to the bacteria, leading to increased potency and a reduction in the overall drug exposure. Phages are viruses that selectively infect bacteria and appear to be safe in clinical trials in humans.^16–19^ While phage therapy typically employs lytic phages to establish an infectious cycle to eliminate the bacterial pathogen, in the current study, phage can also be used as a nanomaterial to deliver the active agent (in this case, PMB), to the bacteria.^20,21^ A well-characterized phage having a high surface area is M13, a rod-shaped phage that is ∼1 μm long and ∼6 nm in diameter, with a 6.4 kb circular ssDNA genome packaged inside its capsid. Wild-type M13 capsids are composed of ∼2700 copies of the major capsid protein pVIII (also called g8p), forming the bulk of the structure, and five copies of each minor capsid protein, including the receptor-binding protein pIII (also called g3p) positioned at one tip.^22,23^ M13 normally initiates a chronic infection of *E. coli* (F+ strains) by attachment of the N-terminal domain of pIII to the conjugative F pilus. However, genetically engineered M13 variants can bind to a wide variety of targets through pIII fusions of antibody fragments.^22^ At the same time, since pVIII carries solvent-exposed carboxylates as well as primary amine groups, M13 phages have many potential sites for chemical conjugation to payloads such as antibiotics.^18,19,24^ Thus, M13 phages can be engineered to bind to specific molecular targets and can be chemically modified to carry a large payload of drug molecules directly to bacteria. Previously, M13 was engineered to bind *Staphylococcus aureus* and release chloramphenicol in the presence of esterases, but this reagent showed limited effect (treatment briefly delayed growth but did not otherwise inhibit growth).^25^ Another in vitro study used an IgG-binding domain to target chloramphenicol-releasing M13, but this approach was acknowledged to pose a problem *in vivo* due to the presence of serum antibodies.^26^ Despite these early efforts, research on phages for antibiotic delivery was characterized as ‘stagnant’ in a recent review.^27^ However, technical advances in phage engineering and understanding of antimicrobial peptides as well as recent demonstrations of the clinical feasibility of phage therapy, combined with the growing need for alternative antimicrobial agents, prompt a reconsideration of the phage delivery concept.

In this study, we engineered a recombinant M13 phage to bind to a wide range of Gram-negative bacteria by targeting lipopolysaccharide (LPS). LPS is composed of three domains: the innermost lipid A, the core oligosaccharide, and the outermost O-antigen (Figure S2).^28^ While the O-antigen is diverse across bacteria, the core oligosaccharide and lipid A structures are relatively conserved.^29^ We designed a single-chain fragment antigen-binding (scFab) version of a known monoclonal mouse antibody that recognizes the LPS core oligosaccharide (WN1 222–5; PDB ID:3 V0 V), to replace the F pilus-binding domain of M13.^30^ We then cross-linked PMB molecules to the phage virions, creating ‘PMB-M13^αLPS^’ (Scheme 1A). Since PMB, like other cationic lipopeptide AMPs, is membrane-active and does not require cell uptake,^31^ PMB-M13^αLPS^ does not require a mechanism for drug release. In addition, polymyxins have been recently shown to create semicrystalline membrane patches (100–300 nm dia.) with LPS,^32^ implicating cooperativity in the mechanism of action. Thus, delivery of a localized concentration of PMB to the outer cell membrane could take advantage of the mechanism of action of PMB. Given the specificity conferred by the scFab domain, localized delivery, and large payload capacity, we hypothesized that PMB-M13^αLPS^ would exhibit high potency and efficacy of bacterial killing at low concentrations, widening the therapeutic window. Here we report *in vitro* studies of PMB-M13^αLPS^ across several Gram-negative pathogenic organisms and *in vivo* studies in mouse models of *P. aeruginosa* pneumonia and corneal infection (Scheme 1B,C).

**Scheme 1:**
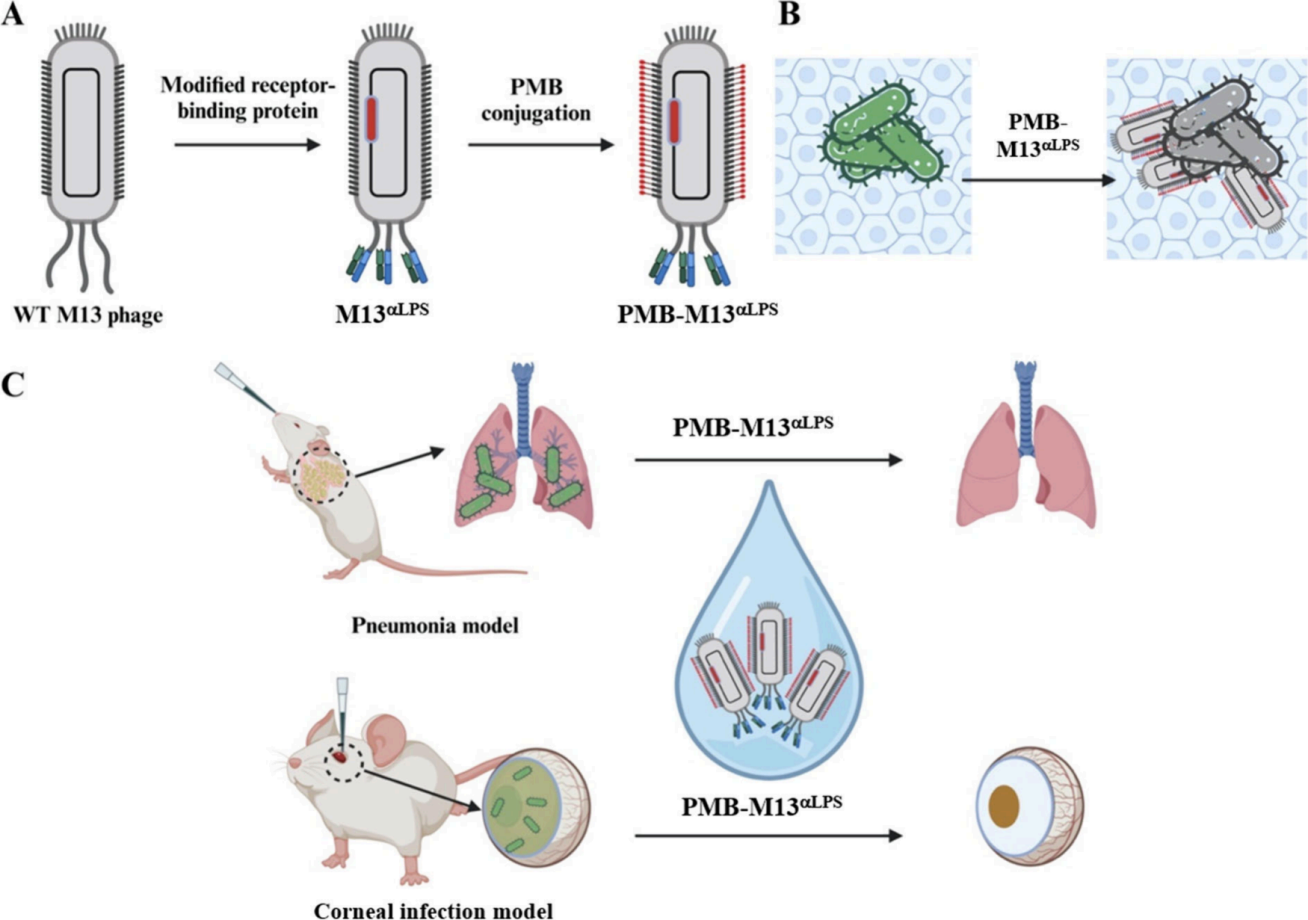
Illustration of PMB-M13^αLPS^ Construction and Application in the Treatment of Gram-Negative Organism Infections^a^

a (A) The receptor-binding protein of wild-type M13 phage was modified to express an anti-LPS antibody scFab, creating M13^αLPS^. Conjugation of PMB molecules to the virion surface yields PMB-M13^αLPS^. (B) PMB-M13^αLPS^ binds specifically to Gram-negative bacteria (green) and not mammalian cells (blue), enabling PMB-mediated death of Gram-negative pathogens. (C) PMB-M13^αLPS^ was tested for treatment efficacy in two mouse models: *P. aeruginosa* pneumonia and *P. aeruginosa* keratitis. Not drawn to scale.

## Materials and Methods

### Materials

All chemical reagents, including polymyxin B sulfate, were purchased from Millipore Sigma unless otherwise specified. All enzymes and buffers used for restriction enzyme digestion, PCR, cloning, and Gibson assembly were purchased from New England Biolabs. Helper phage CM13d3 and phagemid vector pADL-10b were purchased from Antibody Design Laboratories. All oligonucleotides used as primers were purchased from Integrated DNA Technologies and listed in Table S1.

### Bacterial Cultures

Strains used for this project and their sources are given in Table 1. Bacterial strains were revived from glycerol stocks stored at −80°*C* by streaking on LB agar plates and culturing overnight in a 37°*C* incubator. A single colony from each plate was inoculated into a corresponding liquid culture and incubated at 37°*C* with shaking at 220 rpm. To ensure bacteria were fully revived from the dormant state, cells were subcultured >3 times by inoculating 100 μL of overnight culture into 10 mL fresh media prior to use.

**Table 1:** List of Species, Strains, and Sources for Microorganisms Used in This Study

Species	Strain name	Source
*Escherichia coli*	ATCC BAA1161	American Type Culture Collection (ATCC)
*Escherichia coli*	ATCC 700927	ATCC
*Escherichia coli*	DH5alpha	Zymo Research (Irvine, CA)
*Escherichia coli*	ATCC 25922	ATCC
*Pseudomonas aeruginosa*	ATCC 25102	ATCC
*Pseudomonas aeruginosa*	PAKpmrB6 ^33^	Jian Li, Monash University
*Pseudomonas aeruginosa*	Clinical Strain A	UCLA Clinical Microbiology Laboratory
*Pseudomonas aeruginosa*	Clinical Strain B	UCLA Clinical Microbiology Laboratory
*Pseudomonas aeruginosa*	Clinical Strain C	UCLA Clinical Microbiology Laboratory
*Pseudomonas aeruginosa*	Clinical Strain E	UCLA Clinical Microbiology Laboratory
*Pseudomonas aeruginosa*	Clinical Strain F	UCLA Clinical Microbiology Laboratory
*Pseudomonas aeruginosa*	Clinical Strain G	UCLA Clinical Microbiology Laboratory
*Pseudomonas aeruginosa*	Clinical Strain J	UCLA Clinical Microbiology Laboratory
*Pseudomonas aeruginosa*	GFP-PAO1 ^34^	Eric Pearlman, UCI
*Pseudomonas aeruginosa*	ATCC 27853	ATCC
*Pseudomonas aeruginosa*	BCCM/LMG 27624	BCCM
*Pseudomonas aeruginosa*	AR Bank #0246	CDC and FDA AR Bank
*Pseudomonas aeruginosa*	AR Bank #0266	CDC and FDA AR Bank
*Klebsiella quasipneumoniae*	ATCC 700603	ATCC
*Klebsiella pneumoniae*	Clinical Strain A	UCLA Clinical Microbiology Laboratory
*Klebsiella pneumoniae*	Clinical Strain 326	UCLA Clinical Microbiology Laboratory
*Acinetobacter baumannii*	ATCC 19606	ATCC
*Burkholderia cepacia*	ATCC 25416	ATCC
*Staphylococcus aureus*	ATCC 25904	ATCC

### Phagemid Constructs

The sequences of the heavy chain and the light chain of the antibody WN1 222-5 Fab domain were obtained from RCSB Protein Data Bank entry 3V0V.^30^ Two single-chain constructs of the antibody Fab domain were designed with each of the following protein domain orders: light chain - linker - heavy chain (LLH) and heavy chain - linker - light chain (HLL), with the linker sequence being (GGGGS)_3_ (Figure S3). The sequences were codon-optimized by the vendor (Twist Bioscience) for *E. coli* expression and obtained as synthetic DNA. PCR of each synthetic gene fragment was performed with two primers (UpstreamInsert and DownstreamInsert; Table S1), adding NotI and AgeI restriction sites for cloning. Phagemid vector pADL-10b was mutated to insert an AgeI restriction site in the glycine-rich linker between the N2 domain and C-terminal domain of g3p using S239T Forward and S239T Reverse primers (Table S1) and the Q5 Site Directed Mutagenesis Kit (New England Biolabs). Sequence design and visualization was done using Benchling (2024, retrieved from https://benchling.com). The construct was transformed into Mix & Go! Competent Cells-TG1 (Zymo Research) and purified with QIAprep Spin Miniprep Kit (Qiagen), resulting in new phagemid vector pADL-10bS239T. The two synthetic gene inserts were digested with NotI and AgeI restriction enzymes, run on a 1% agarose gel, and purified using a Qiaquick gel extraction kit (Qiagen). The vector pADL-10bS239T was digested and purified in the same way, and then ligated to each of the digested inserts using ElectroLigase, followed by transformation into Mix & Go! Competent Cells-TG1 (Zymo Research). Clones were confirmed by Sanger sequencing using sequencing primers pADL-sequencing Forward and pADL-sequencing Reverse (Table S1; Text S1).

### Production and Purification of Recombinant M13^αLPS^ Phages

Phage virions were produced following the helper phage CM13d3 user manual (Antibody Design Laboratories, San Diego, CA) as follows. *E. coli* TG1 cell strains with phagemid vectors of interest were inoculated in 5 mL of 2xYT media (100 μg/mL ampicillin and 1% w/v glucose) and grown overnight at 37 °C under shaking at 250 rpm. The overnight culture was subcultured into 50 mL of 2xYT media (100 μg/mL ampicillin) and incubated with shaking until OD_600_ reached 0.5. Helper phage CM13d3 was defrosted and vortexed; 1 μL helper phage was added per 1 mL of cell culture for superinfection. The culture was incubated for 1 h at 25 °C followed by addition of stock solutions to reach 50 μg/mL kanamycin and 200 μM IPTG concentration. The phages were produced by allowing the culture to grow for 8 h to overnight by incubating at 25 °C and shaking at 200 rpm. The cell solution was spun down at 5,000*g* for 5 min in a benchtop centrifuge and the supernatant was collected. The produced phages were purified following a revised version of a published protocol.^35^ The supernatant containing the phages was filtered through a 0.22 μm membrane filter to remove the majority of cell debris. The filtrate was then concentrated using a 30 kDa MWCO Amicon protein desalting column (Millipore Sigma) and washed with 1 × PBS buffer 5 times by resuspending the concentrate in 1 x PBS after each round of centrifugation. The retentate was then collected and resuspended in a desired volume of 1 x PBS buffer for further characterization. The concentration of phage virions was determined by UV absorbance spectrometry at 269 and 320 nm, using the equation $\frac{virions}{mL}=\frac{\left(A_{269}-A_{320}\right)\times 6\times 10^{16}}{\frac{number\;\mathrm{of}\;bases}{phage\;genome}}$ and confirmed by qPCR quantification as described below. For short-term storage (less than 12 weeks), phage solutions were stored at 4 °C.^36^

### Binding Assay for Phage to Bacteria

To quantify the amount of phage binding to different bacterial cell targets, 10^5^ to 10^11^ virions in 100 μL volume were mixed with 1 mL of bacterial cells in the exponential growth phase at a concentration of OD_600_ = 1 (approximately 10^8^ cells/mL).^37^ Bacteria and phages were incubated at room temperature (RT) on a rotator for 30 min and then centrifuged at 4,000*g* for 5 min. Bacteria were washed with 1 x PBS buffer to remove unbound or nonspecifically bound phage and resuspended in 1x PBS buffer. Bound phage DNA in each sample was extracted using a miniprep procedure and quantified using qPCR as described below. A negative control consisting of bacteria without phage was performed in parallel, and the corresponding quantity was used for background subtraction.

### Quantitative Polymerase Chain Reaction (qPCR) Assay

To quantify the number of phage virions in a sample, 100 μL of phage sample was processed following the standard miniprep protocol using QIAprep Spin Miniprep Kit (Qiagen), and the phage DNA was eluted in 50 μL of Milli-Q water. The quantity of phagemid present in eluted samples was quantified using qPCR with a standard curve made from a known concentration standard of purified phagemid vector pADL10b. The qPCR assay was carried out using SYBR Green Master Mix (Bio-Rad Laboratories) with primer set qPCR-upstream and qPCR-downstream on a Bio-Rad C1000 PCR machine. PCR conditions were: 95 °C for 30 s followed by 45 cycles of denaturation for 30 s at 95 °C and extension for 15 s at 60 °C. Data collection was performed using the CFX Maestro Software and data analysis was performed using Microsoft Excel.

### Transmission Electron Microscopy (TEM)

Negatively stained TEM samples were prepared following our previously published protocol.^38^ Bacterial cell culture in stationary phase was spun down at 4,000 rpm for 5 min, washed once with 1 x PBS buffer and resuspended in 1 x PBS buffer to OD_600_ value between 2 and 3. 100 μL of the cells were incubated with either 1 x PBS as control, or 10 μL of phages at a concentration of 1 × 10^11^ virions/mL, for 30 min at RT. The samples were spun down and washed twice with 1 x PBS buffer to remove any unbound or nonspecifically bound phages and resuspended in 1 x PBS buffer to the original volume. Eight μL of samples were loaded on the shiny side of Formvar/Carbon 200 Mesh, Ni TEM grid (Electron Microscopy Sciences) and incubated for 2 min. The grid was washed 4 times in filter-sterilized wash buffer composed of 1% bovine serum albumin in 1 x PBS buffer. The sample was then blocked with blocking buffer, composed of filter-sterilized 0.1% gelatin in 1 x PBS buffer, at RT for 1 h, washed once, and incubated with 100 μL of 1:100 dilution anti-M13 major coat protein monoclonal antibody (ThermoFisher Scientific) or Polymyxin B Monoclonal Antibody (ThermoFisher Scientific) for 1 h at RT. The samples were then washed with the same wash buffer 4 times, blocked with blocking buffer, and incubated with 100 μL of 1:20 dilution of preadsorbed secondary antibody-coated 6 nm gold nanoparticles (Abcam) for 1 h at RT. The sample was washed with Milli-Q water 5 times and stained with 8 μL of 1% uranium acetate for 1.5 min, dried and imaged on a FEI Tecnai T12 transmission electron microscope using the DigitalMicrograph software (California NanoSystems Institute at UCLA) and processed using ImageJ.

### Cell-Based Enzyme-Linked Immunosorbent Assay (ELISA) for Phage Binding

5 mL cultures of *E. coli* strains DH5α or ER2738 (New England Biolabs) were grown overnight in LB broth, with tetracycline added at 10 μg/mL for ER2738. The following day, 1 mL of each culture was centrifuged for 5 min at 5000 rpm. The supernatant was discarded, and cells were resuspended in 1 mL of PBS. Cells were centrifuged under the same conditions and resuspended in fresh PBS. The OD600 of the cell resuspension was adjusted to 1, and the cells were diluted 100-fold in PBS. 200 μL of diluted cells or blocking buffer (5 mg/mL BSA, 0.1 M NaHCO_3_, pH 8.6) per well were added to 96-well flat bottom Nunc plates with MultiSorp coating (ThermoFisher) and incubated overnight at 4 °C to allow cell attachment. The following day, the solution was shaken out and replaced with 200 μL of blocking buffer and then incubated for 2 h at 4 °C. Plates were washed thoroughly by pipetting 200 μL of Tris-Buffered Saline with Tween 20 (TBST) into each well with a multichannel pipet, shaking out excess solution, and slapping face down on a paper towel. This washing process was repeated six times. A series of M13 or M13^αLPS^ phage dilutions from 5 × 10^11^ to 10^8^ virions/mL in PBS were prepared. 100 μL of phage at the appropriate concentration was added to each well, then incubated for 2 h at room temperature. Plates were washed again before adding 200 μL of anti-M13-g8p-HRP antibody (Antibody Design Laboratories) at a concentration of 0.2 μg/mL in PBS supplemented with 5 mg/mL BSA, and incubated for 1 h at room temperature. Plates were washed again before adding 100 μL of TMB substrate solution (ThermoFisher) and incubating for 15 min at room temperature. The reaction was stopped by adding 50 μL of 2 N sulfuric acid, and absorbance at 450 nm was read using a Tecan Infinite M200 PRO plate reader.

### Conjugation of Polymyxin B to M13^αLPS^ to Produce PMB-M13^αLPS^

Polymyxin B (PMB) molecules were conjugated on the surface of recombinant M13 phages using carbodiimide cross-linker chemistry. Surface-exposed primary amines of M13^αLPS^ were first blocked with sulfo-NHS-acetate to prevent phage cross-linking. The reacted product was dialyzed (1:45 volume ratio) 2 times in 1 x PBS buffer and 3 times in 0.1 x MES buffer (pH 5.5) for 4 h per dialysis cycle at 4 °C, using a Slide-A-Lyzer Mini Dialysis Device (20K MWCO, Fisher Scientific, Waltham, MA). The dialyzed product was incubated with 100 μL of EDC (4 mg/mL) and Sulfo-NHS (11 mg/mL) mixture dissolved in water for 20 min. The reacted product was dialyzed 3 times in 1 x PBS buffer for 40 min per dialysis cycle at 4 °C. The dialyzed product was transferred to a 15 mL tube, and 1 x PBS buffer was added to reach a final volume of 10 mL. 0.75 mg of PMB was then mixed and incubated with each 1 mL (∼1 × 10^12^ virion/mL) of phage for 2 h. The samples were then dialyzed 2 times with 1x TBS buffer (pH 7.2) to quench the reaction and 3 times with 1 x PBS buffer to remove excess unreacted PMB. Amino acid composition analysis was performed on 435 μg of PMB-M13^αLPS^ (pooled from 5 independently synthesized batches) by Creative Proteomics (Shirley, New York).

### Quantification of Primary Amines on Polymyxin B, Sulfo-NHS Acetate Blocked M13^αLPS^, and PMB-M13^αLPS^

50 μL portion of Fluoraldehyde o-Phthaldialdehyde Reagent Solution (OPA) (Fisher Scientific) was mixed with 200 μL of samples diluted in 1 x PBS buffer supplemented with 0.5% v/v Triton X-100 (*N* = 3). The reactions were incubated at room temperature in the dark for 10 min. Fluorescence signals were measured by fluorescence plate reader at 350 nm excitation/450 nm emission (Tecan Infinite M200 PRO, Tecan Group Ltd., Switzerland) using the i-control software and analyzed using Microsoft Excel.

### Minimal Inhibitory Concentration and Minimal Bactericidal Concentration Assays

The minimal inhibitory concentrations (MIC) of PMB and PMB-M13^αLPS^ for different pathogen species and strains were determined following published broth microdilution protocols in triplicate samples.^39,40^ In brief, in nontreated and sterile U-Shaped-Bottom 96 well plates (Fisher Scientific), 50 μL of PMB at concentrations ranging from 1 to 256 μg/mL, or PMB-M13^αLPS^ at concentrations ranging from 2.5 × 10^9^ to 8 × 10^10^ virions/mL, in 2-fold serial dilutions with Muller-Hinton broth (MH broth), were mixed with 50 μL of bacterial cells (5 × 10^5^ colony forming units (CFU)/mL) in each well. 100 μL of MH broth with or without the same concentration of bacteria were used as growth controls and sterility controls, respectively. The sterilely covered 96-well plate was incubated at 37°*C* overnight without shaking and cell growth in each well was examined by spectrophotometry at 600 nm on a plate reader (Tecan Infinite M200 PRO, Tecan Group Ltd., Switzerland) through the i-control software and analyzed using Microsoft Excel. The well with the lowest antibiotic concentration without significant growth (OD_600_ increase <0.1 compared to sterility control) indicated the value of the MIC. Subsequently, 50 μL of culture from each well having no significant growth was spread uniformly on a Muller-Hinton plate and incubated overnight. The lowest concentration of PMB or PMB-M13^αLPS^ yielding a plate with no cell colonies indicated the minimal bactericidal concentration (MBC). The MIC assays for *P. aeruginosa* strains ATCC 27853, LES 431, AR-Bank no. 0246, and AR-Bank no. 0266 were conducted by Pharmacology Discovery Services (PDS).

### *P. aeruginosa* Biofilm MBC Assay

The MBC of PMB and PMB-M13^αLPS^ for biofilm-forming strain *P. aeruginosa* strain ATCC 25102 was determined based on published broth microdilution protocols in quadruplicate samples.^37,39–41^ In brief, in nontreated and sterile U-Shaped-Bottom 96 well plates (Fisher Scientific), 150 μL of MH broth with 5 × 10^5^ CFU of *P. aeruginosa* strain ATCC 25102 were incubated in each well for 3 days under room temperature, covered by a sterile lid. By day 3, a bacterial floc(s) could be observed near the bottom of each well. 150 μL of PMB (200, 240, 280, 320, 360, or 400 μg/mL) or PMB-M13^αLPS^ (1.25 × 10^11^ to 4 × 10^12^ virions/mL in 2-fold serial dilutions) in MH broth were added to each well without disturbing the bacterial flocs. The sterilely covered 96-well plate was incubated at room temperature overnight without shaking, and cell viability in each well was examined by spreading uniformly on a MH plate with incubation overnight at 37 °C. The lowest concentration of PMB or PMB-M13^αLPS^ yielding a plate having no cell colonies indicated MBC for the biofilm.

### Time-Kill Kinetics Assay

PMB and PMB-M13^αLPS^ stocks in 1 x PBS were diluted into MH broth to a concentration 2x their respective MIC. For PMB-M13^αLPS^, a stock of 10^12^ virions/mL in 1x PBS was diluted 100-fold into MH broth for a concentration of 10^10^ virions/mL (2 x MIC). 50 μL of the diluted PMB or PMB-M13^αLPS^ was added to 50 μL of *E. coli* culture grown in MH broth (final dilution of PMB-M13^αLPS^ = 200-fold). Following established protocols,^42,43^ in 96-well plates, the resulting cultures containing PMB and PMB-M13^αLPS^ at the concentration equal to the MIC and 5 × 10^5^ CFU/mL of bacterial cells were incubated at 37°*C*. 100 μL of cultures from three triplicate wells were plated on individual Muller-Hinton plates at 0.5, 1, 2, 3, 4, 5, 6, 7, and 8 h time points. The plates were incubated at 37°*C* overnight, and the number of CFUs was determined for each time point. Data were analyzed using Microsoft Excel [v.16.16] and Origin64 version 2024b.

### Biocompatibility with Mammalian Cells Assayed with MTT

Cell metabolic activity in the presence of different PMB and PMB-M13^αLPS^ concentrations was measured using 3-(4,5-dimethylthiazolyl-2)-2,5-diphenyltetrazolium bromide (MTT) assay with human embryonic kidney (HEK-293) cells (ATCC CRL-1573) following published protocols.^44,45^ Cells were cultured in Dulbecco’s Modified Eagle’s Medium (DMEM) supplemented with 10% fetal bovine serum (FBS) and 1% antibiotics (penicillin/streptomycin) using Falcon polystyrene tissue culture flasks with ventilated caps (Corning). The cells were incubated at 37 °C with 5% CO_2_ until confluency. Then, cells were detached from the culture flask, seeded at a density of 1 × 10^4^ cells/well, and incubated with DMEM media in each well of 24-well plates (Corning). Five hours after seeding, the media was changed to DMEM containing 10% FBS and 1% antibiotics (penicillin/streptomycin) supplemented with either PMB (at concentrations of 2, 8, and 32 μg/mL), PMB-M13^αLPS^ (at concentrations of 5 × 10^9^, 1 × 10^10^, and 2 × 10^10^ virions/mL) or no supplements as the negative control. Triplicate samples were prepared for each condition, and media was refreshed every 2 days with fresh media supplemented with the corresponding concentrations of PMB and PMB-M13^αLPS^. HEK-293 cell metabolic activity was quantified using an MTT assay according to the manufacturer’s protocol. After 1, 3, and 5 days of culture, 100 μL of MTT stock solution (5 mg/mL) was added to each well. After 4 h of incubation at 37 °C with 5% CO_2_, 1 mL of solubilization solution was added and left overnight. The next day, the absorbance of the wells was read at 550 nm (reference wavelength of 650 nm) using a Tecan Infinite M200 PRO plate reader. Data analysis was performed using Microscoft Excel (v.16.16) and Origin64 version 2024b.

HEK-293 cell spreading and morphology for cells incubated under all conditions described above was observed using AlexFlour 594 (phalloidin) and 4′,6-diamidino-2-phenylindole dihydrochloride (DAPI) (Invitrogen) staining. After 1, 3, and 5 days of culture, the cells were fixed using 4% (v/v) paraformaldehyde for 10 min. Then, the cells were washed with 0.3% (v/v) Triton X-100 for 10 min and blocked with 1% bovine serum albumin (BSA) for 30 min. A light-sensitive mixture of 0.1% (v/v) phalloidin and 0.05% (v/v) DAPI in 1 x PBS was added to stain the cells for 20 min. All procedures were conducted at RT. After washing with DPBS, the cells were imaged with an AxioObserver Z1 inverted microscope (Zeiss) by using the Zen Lit [v3.10] software.

### *In Vitro* Hemolysis Assay

Hemolysis assays were performed following previously established protocols to determine and compare the hemolytic activity of PMB and PMB-M13^αLPS^ at serially diluted concentrations.^40,46^ Five mL of 10% sheep red blood cells (MP Biomedicals) were washed five times by spinning down at 500*g* for 5 min, removing the supernatant, and refilling to the original volume with 1 x PBS buffer for gentle resuspension. After the last washing step, cells were resuspended in 1 x PBS buffer to obtain a 4% red blood cells solution. 100 μL of the 4% solution was added into each 1.5 mL sterile Eppendorf tube and mixed with 100 μL of each sample, 1 x PBS for negative control, or 2% Triton X-100 for positive control (N = 3). The mixtures were incubated for 1 h at 37 °*C*. The samples were then spun down at 500x g for 5 min, and 100 μL of supernatant was transferred into 96-well plates. Absorbance at 405 nm was measured using a plate reader and the hemolytic activity was determined using the following equation:

$$\mathrm{\% hemolysis}=\left(A_{sample}-A_{PBS}\right)/\left(A_{\mathrm{Triton X‐100}}-A_{PBS}\right)$$

*A*_sample_, *A*_PBS_, and *A*_Triton X‑100_ are absorbance values at 405 nm of the sample, negative control, and positive control correspondingly. Data was processed using Microsoft Excel [v.16.16] and Origin64 version 2024b.

### *In Vivo* Maximum Tolerated Dose and Toxicity

These experiments were conducted under protocol ARC-2020–044-AM approved by the UCLA Animal Research Committee (IACUC). Male 10-week-old C57BL/6J mice (24–30 g, The Jackson Laboratory) were used for this study. Mice were housed in rigid, nonporous, leak-proof containers (maximum 4 animals per cage) that were compatible with biohazardous animals. Intravenous (IV) tail vein injections were performed using BD Ultra-Fine Insulin Syringes 30G 3/10 cc 1/2 in. (Becton Dickinson). 100 μL of PMB-M13^αLPS^ or PMB at different concentrations in 1 x PBS buffer were administered to each animal through IV tail vein injection once a day for seven consecutive days (N = 3). 24 h after the last injection, 350 μL of blood was collected through retro-orbital bleeding from each animal under 2% isoflurane anesthesia, into a BD Microcontainer Tube with Serum Separator (Becton Dickinson). The animals were euthanized through decapitation under anesthesia. The left kidney, spleen, and liver samples from each animal (size of approximately 5 mm × 5 mm × 3 mm) were collected. The blood samples were spun down at 1,000*g* for 10 min after allowing the blood to clot for 30 min at 4°*C*. The serum was collected in sterile 1.5 mL Eppendorf tubes, stored at 4 *°*C, and sent for biomarker analysis (Standard Tox Panel 62794 analysis performed by IDEXX Bioanalytics, USA). The tissues were soaked in formalin for 24 h before being transferred into 70% ethanol for downstream H&E staining.^47^ Data was processed using Microsoft Excel [v.16.16] and Origin64 version 2024b.

### μPET/CT Study of PMB-M13^αLPS^ Biodistribution

These experiments were conducted under protocol ARC-2020–044-AM approved by the UCLA Animal Research Committee. Positron emission tomography (PET) imaging was used to characterize the biodistribution of PMB-M13^αLPS^ in male 10-week-old C57BL/6J mice, 24–30 g (The Jackson Laboratory), at time points across a 72 h period, for conditions with and without bacteria in the bloodstream (N = 4). PMB-M13^αLPS^ virions were radioactively labeled with ^89^Zr by first covalently conjugating with chelator desferrioxamine (DFO).^48^ The isothiocyanate-bearing derivative of DFO p-SCN-Bn-desferrioxamine B (DFO-NCS) was dissolved in dry DMSO at a concentration of 5 mg/mL. Five mL of PMB-M13^αLPS^ at a concentration of 5 × 10^11^ virions/mL in 1 x PBS were adjusted to pH 9 with small aliquots of 0.1 M Na_2_SO_4_. Twenty-five μL of DFO-NCS were added to PMB-M13^αLPS^ and incubated for 1 h on a rotator at RT.^49^ The product DFO-PMB-M13^αLPS^ was purified from unreacted DFO-NCS and DMSO through 5 x dialysis cycles (4 h each cycle in 1 x PBS buffer using a Slide-A-Lyzer dialysis cassette). DFO-PMB-M13^αLPS^ was further conjugated with ^89^Zr and purified similarly. Radioactivity was determined using radio-thin layer chromatography. For the group of animals with bacteria, 1 × 10^7^ CFU of the *E. coli* strain ATCC BAA 1161 in 1 x PBS, diluted from samples with OD_600_ = 0.5 (Table S2) were injected in 50 μL volume via tail vein, 2 h before injection of ^89^Zr-DFO-PMB-M13^αLPS^ with 100 μCi radioactivity (∼5 × 10^10^ virions). The mice were scanned using μPET/CT at time points of 10 and 30 min and 1, 2, 4, 6, 24, 48, and 72 h. One animal in the bacterial injection group died after 24 h and one injection failed for an animal in the group without bacterial injection, leaving N = 3 for each group. The images were acquired with an energy window between 350 to 650 keV, generated using the 3D-OSEM/MAP method, and analyzed using the software AMIDE v0.9.0. Data were analyzed using Microsoft Excel [v.16.16] and Origin64 version 2024b.

### *In Vivo* Effect of PMB-M13^αLPS^ in a *P. aeruginosa* Mouse Lung Infection Model

Pathogen-free 7-week-old female BALB/c mice (specific pathogen-free (SPF)) were obtained from BioLASCO Taiwan, an AAALAC-certified Charles River Licensee and rodent breeder. Mice were quarantined for 3 days after receipt in an SPF facility before transfer to the vivarium. During infection studies, animals were housed in a separate room in negative-pressure individually ventilated cages (GM500 IVC seal safe cage system; Tecniplast, Italy) and supplied with sterile bedding and gamma-irradiated food. *P. aeruginosa* strain AR Bank #0266 was prepared from a 0.2 mL aliquot of a single-use glycerol stock seeding 20 mL of Tryptic Soy Broth and then incubated at 35–37°*C* with shaking (250 rpm) for 16 h. 0.5 mL of the culture was used to seed 49.5 mL of fresh media and incubated in the same manner for 3 h. The cells were then pelleted, resuspended in 10 mL of 1 x PBS, and quantified by optical density at 620 nm. The cells were then diluted to desired concentration using 1 x PBS and inoculum was administered within 1 h of time. The inoculum size ((2–4) × 10^7^ CFU) was estimated to be a lethal dose (LD90) in a titration study. On Day 0, after anesthesia with pentobarbital (50 mg/kg, IP), animals were infected with 2.8 × 10^7^ CFU of pathogen suspension (*P. aeruginosa* strain AR Bank no. 0266) by intranasal (IN) administration in 0.05 mL volume. Each mouse was held upright and 25 μL of the bacterial suspension was gradually released into each nostril using a micropipette. The mouse was held in an upright position until the breathing rate and depth renormalized (approximately 2 min) and then placed into the cage for recovery. The test articles (PMB, PMB-M13^αLPS^, M13^αLPS^ and wild-type M13 phage) were formulated in 1× PBS. One hour past infection, animals used to count disease baseline CFUs were sacrificed with CO_2_ asphyxiation for aseptic lung tissue collection, and other animals were subject to treatment. For treated animals, an additional anesthetic dose (pentobarbital 25 mg/kg at 30 min postinfection) was administered IP and the treatment administered IN in the manner described. Animals were observed at 30 min and 12 h after IN dosing for signs of acute toxicity; none were found to have reached humane end points prior to the scheduled time for euthanasia. Twenty-five h postinfection, the treated animals were sacrificed with CO_2_ asphyxiation for aseptic lung tissue collection. The lung tissues were weighed and homogenized in 1 mL sterile 1 x PBS buffer using a Polytron homogenizer. The bacterial burden in the tissue homogenates was determined by performing 10-fold serial dilutions and plating 0.1 mL of each onto MacConkey II agar. Colonies were counted after 18–24 h incubation, and the dilution plate yielding the most colonies (10–300) was used for calculation. The CFU value per tissue was calculated with the following equation:

$$\frac{\mathrm{CFU}}{\mathrm{lung}}=\frac{\frac{\mathrm{CFU}}{\mathrm{plate}}}{\frac{\mathrm{mL sample plated}}{\mathrm{plate}}}\times \mathrm{dilution factor}\times \mathrm{total sample volume}\left(\frac{\mathrm{mL}}{\mathrm{tissue}}\right)$$

Bacterial burden (CFU/tissue) of treated animal groups was compared to the baseline bacterial count at 1 h after infection and to animals treated with 1 x PBS as a negative control. The significance of effects was assessed with ANOVA followed by Dunnett’s test using GraphPad Prism software. Experiments for this infection model were performed by Pharmacology Discovery Services (PDS) under PDS IACUC protocol IM002-02082022, under NIAID Contract No. HHSN272201700020*I*/75N93023F00002. Data were analyzed using Microsoft Excel [v.16.16] and Origin64 version 2024b.

### *In Vivo* Effect of PMB-M13^αLPS^ on a *P. aeruginosa* Corneal Infection Model

Age-matched male and female 6–8 week-old C57BL6/J (WT) mice were purchased from The Jackson Laboratory (Bar Harbor, ME) and bred and housed in a UC Irvine vivarium. *P. aeruginosa* GFP-expressing strain (PAO1-GFP) bacteria were grown from frozen stock in a high-salt Luria–Bertani broth (BD Biosciences) supplemented with 50 μg/mL carbenicillin. The culture was incubated at 37 °C overnight. 100 μL of the overnight culture was subcultured into new culture media and grown to mid log phase at 37 °C with 5% CO_2_ with agitation until an OD of 0.2 was reached. Bacteria were then centrifuged at 3,000*g* for 10 min, resuspended in sterile PBS, and diluted to 2.5 × 10^7^ cells/mL. Mice were anesthetized according to IACUC approved standards using ketamine/xylazine, and the corneal epithelium was abraded using a sterile 30-gauge needle (BD Biosciences) to create three parallel 1 mm scratches. After the corneas were abraded, 2 μL of 5 × 10^4^ bacteria was applied topically. At 2, 20, and 24 h postinfection, mice were given 10 μL of phage or PMB-M13^αLPS^ topically, and the lids were closed for 1 min. Mice were euthanized at 48 h postinfection and eyes were imaged for corneal opacity and GFP quantification using a Leica MZ10 F Modular Stereo Microscope for fluorescent imaging with a Leica DFC450 C camera. Brightfield and GFP fluorescent images were taken using the following settings: Zoom: 3.2x, brightfield exposure: 40 ms, fluorescence exposure: 2 s. For CFU quantification, whole eyes were collected and placed in 1 mL of sterile 1 x PBS containing a sterile steel ball bearing (5 mm). Eyes were homogenized using a Qiagen TissueLyser II at 30 Hz for 3 min. Homogenates were 10-fold serially diluted, and 10 μL of each dilution was plated onto LB agar plates. Plates were incubated at 37 °C with 5% CO_2_ overnight, and colonies were counted manually the next day. CFUs were calculated following the equation of:

$$\frac{\mathrm{CFU}}{\mathrm{eye}}=\frac{\frac{\mathrm{CFU}}{\mathrm{plate}}}{\frac{\mathrm{mL sample plated}}{\mathrm{plate}}}\times \mathrm{dilution factor}\times \mathrm{total sample volume}\left(\frac{\mathrm{mL}}{\mathrm{tissue}}\right)$$

These experiments were conducted under protocol AUP-21-123 approved by the UC Irvine Institutional Animal Care and Use Committee. Data were analyzed using Microsoft Excel [v.16.16] and Origin64 version 2024b.

## Results

### Construction, Production, and Initial Binding Tests of Recombinant M13^αLPS^

The gene fragments encoding two different constructs of antibody WN1 222-5 as single-chain fragment antigen-binding regions (scFab) were cloned into the phagemid vector pADL10b-S239T, replacing the wild-type pIII N-terminal domains (N1 and N2). The two constructs differed in ordering of the heavy and light chains, and a glycine linker (GGGGS)_4_ was placed between the two chains (Figure S3). Construct HLL had the order heavy chain–linker–light chain, and the construction of LLH had the order light chain–linker–heavy chain. Clones of the constructed phagemids were confirmed by Sanger sequencing (Text S1). Phage virions were produced and purified by standard methods, quantified by UV absorbance spectrum (Figure S4) and verified by TEM (Figure 1A,B).^50^

**Figure 1:**
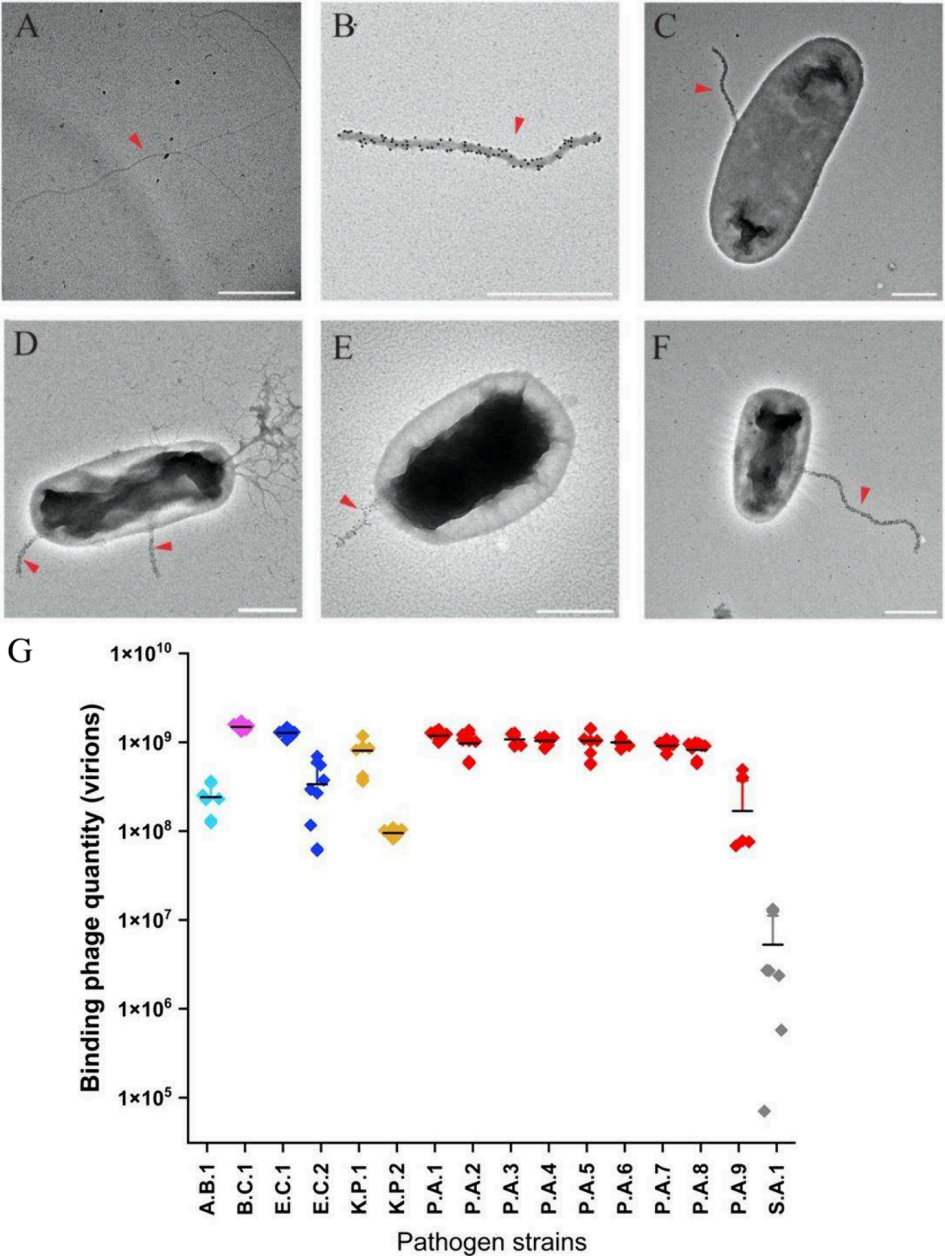
M13^αLPS^ and binding to Gram-negative pathogens. Transmission electron microscopy (TEM; negative stain) images show that recombinant M13 phages have rod-like filamentous morphology (A). To differentiate M13^αLPS^ from bacteria pili and other structures, M13^αLPS^ were labeled using a mouse monoclonal anti-pVIII primary antibody, followed by gold nanoparticles (dark spheres) coated with donkey antimouse secondary antibody, allowing phages to be easily identified (red darts). Gold-labeled M13^αLPS^ is shown (B) without bacteria; or incubated with (C) *E. coli* DH5α; (D) *P. aeruginosa* strain ATCC 25102; (E) *K. quasipneumoniae* strain ATCC 700603; and (F) *A. baumannii* ATCC 19606. Note that the phage width is increased by the labeling reagents. See Figure S7 for more bacterial species. Scale bars: 500 nm. (G) Saturating amount of M13^αLPS^ bound to various bacterial strains and clinical isolates determined by qPCR. The bacterial strains are *A. baumannii* ATCC 19606 (A.B.1, sky blue); *B. cepacia* ATCC 25416 (B.C.1, pink); *E. coli* (E.C., navy blue) 1: DH5alpha, 2: ATCC BAA 1161; *K. quasipneumoniae* 1:ATCC 700603 and *K. pneumoniae* 2:clinical strain A (K.P., brown),; *P. aeruginosa* (P.A., red) 1:ATCC 25102, 2:clinical strain A, 3:clinical strain B, 4:clinical strain C, 5:clinical strain E, 6:clinical strain F, 7:clinical strain G, 8:clinical strain J, 9:PAKpmrB6; *S. aureus* ATCC 25904 (S.A.1; gray). All Gram-negative strains show substantial binding compared to *S. aureus*. Mean value (solid black dash) and error bars (1 standard deviation, shown in the + direction) were calculated from three experimental replicates, each of which included three technical replicates (except for P.A.3 which was performed in experimental duplicates).

Without the N-terminal domain of pIII, the recombinant phages were noninfectious and do not form plaques. Since plaque-forming assays could not be used to quantify binding or infection, binding of recombinant phages to several species of Gram-negative bacterial cells was tested, for both HLL and LLH constructs, by incubation of phages with cells, pelleting and washing of cell-phage complexes, and phagemid-specific qPCR of isolated DNA. In general, the HLL construct showed approximately 1 order of magnitude more phage binding to each target pathogen (*E. coli* strain DH5α; *P. aeruginosa* strain ATCC 25102 and clinical strains B, C, E, F, G, J; *K. quasipneumoniae* strain ATCC 700603, *K. pneumoniae* clinical strain A; *A. baumannii* ATCC 19606; *B. cepacia* ATCC 25416; and *C. sakazakii* ATCC 25944) compared to the LLH construct (Figures S5–S6, Table S3). Since the HLL construct demonstrated stronger binding than the LLH construct, the HLL construct was selected for downstream experiments and termed ‘M13^αLPS^’ hereafter.

### Binding of M13^αLPS^ to Various Gram-Negative Bacterial Species

LPS has three distinct domains, namely, a core oligosaccharide bridging lipid A in the outer membrane and an O-antigen exposed to the external environment. The O-antigen contributes greatly to LPS diversity, with more than 180 reported O-antigen serotypes for *E. coli* alone.^51^ In contrast, the core oligosaccharide, composed of an inner core of keto-deoxyoctulose and heptose residues and an outer core of hexoses, is relatively conserved. The antibody WN1 222-5 recognizes the inner core and is the only reported antibody up to today to be able to specifically recognize a large number of pathogenic Gram-negative pathogens including *E. coli*, *Salmonella*, *Shigella*, and *Citrobacter* species.^30^ Thus, M13^αLPS^ was expected to bind a wide range of Gram-negative organisms. Binding was visualized by TEM of phage incubated with bacterial cells, using an anti-M13 primary antibody and gold-nanoparticle-labeled secondary antibody to positively identify the phages^38^ (Figure 1C–F, Figure S7). M13^αLPS^ were observed to be bound to cell surface structures via the virion tip (not sidewall), consistent with the expected virion structure that should display the modified receptor-binding protein at the tip (Scheme 1A).

The amount of M13^αLPS^ bound to the cells was quantified using the qPCR method described above, by mixing 1 mL of cells (OD_600_ = 1) with 100 μL of phage (10^5^ to 10^11^ virions), pelleting of phage-cell complexes, washing, and qPCR of extracted DNA to determine the number of phagemid copies isolated. For comparison, a Gram-positive bacterial strain (*Staphylococcus aureus* strain ATCC 25904) was used as a negative control since Gram-positive organisms lack LPS. In general, for phage concentrations ≥ 10^7^ or 10^8^ virions/mL (depending on the strain), the amount of phage bound to Gram-negative strains exceeded the amount bound to *S. aureus* by 1–2 orders of magnitude (Figure 1G, Figures S8–S10, Table S4). Among the Gram-negative strains tested (*E. coli*, *P. aeruginosa*, *K. pneumoniae*, *A. baumannii*, and *B. cepacia*), some strains exhibited greater binding than others, but the saturating amounts bound were within the same order of magnitude (Figure 1G). These results indicate that M13^αLPS^ exhibits binding to a range of Gram-negative organisms, consistent with its design.

Binding of M13^αLPS^ to *E. coli* was also verified by cell-based ELISA. *E. coli* cells were directly attached to plates and incubated with a solution containing M13 or M13^αLPS^ in varying amounts. Attached phages were detected by an anti-g8p antibody. The assays showed that M13 bound to an F+ strain (ER2738) but not an F- strain (DH5α), as expected. In contrast, M13^αLPS^ showed a similarly high amount of binding to both ER2738 and DH5α, consistent with its design. The ELISA results confirmed that engineering g3p with the anti-LPS sequence broadened the binding range of M13^αLPS^ compared to that of M13 (Figure S11).

### Conjugation of Polymyxin B to M13^αLPS^

To deliver PMB using M13^αLPS^, polymyxin B (PMB) molecules were conjugated to purified M13^αLPS^ using carbodiimide cross-linking (EDC) chemistry to form PMB-M13^αLPS^. PMB contains multiple primary amines from 2,4-diaminobutyric acid (Dab) side chains of amino acids on the peptide ring, and the major phage coat protein pVIII contains three solvent-exposed carboxylic acid residues (Glu2, Asp4 and Asp5) per copy.^52,53^ Since pVIII also contains primary amines, phage cross-linking was prevented by first blocking M13^αLPS^ using sulfo-NHS-acetate. The ratio of phage to PMB in the cross-linking reaction was empirically optimized by incubating 1 × 10^12^ virions of M13^αLPS^ with an increasing amount of PMB in 1 mL volume reaction to obtain the highest potency *in vitro*, i.e., the lowest minimum inhibitory concentration (MIC) (see next section).

The amount of PMB conjugated per virion was determined by using two methods: amino acid composition analysis and quantitation of primary amines. In amino acid composition analysis, the mole percentage of different amino acids (Asx (Asn + Asp), Thr, Ser, Glx (Gln + Glu), Pro, Gly, Ala, Val, Ile, Leu, Phe, His, Lys, Arg, and Tyr) was measured experimentally by acid hydrolysis of PMB-M13^αLPS^ and chromatographic separation of amino acids (Figure S12 **)**. The measured percentages were compared to the theoretically expected mole percentages calculated from the known sequences of M13^αLPS^ proteins, for varying ratios of PMB molecules conjugated per g8p protein (Table S5, Text S2).^54^ The amino acid composition analysis indicated that on average, ∼2 PMB molecules were conjugated to each copy of pVIII, or ∼5400 PMB carried by each virion (1.1 × 10^–11^ μg of PMB per virion). This value was used for conversion of virions to the PMB amount.

In addition, primary amines were quantified using the Fluoraldehyde o-Phthaldialdehyde Reagent Solution (OPA) assay for derivatization to fluorescent products. A standard curve for fluorescence at varying PMB concentration was generated **(** Figure S13). The fluorescence of derivatized PMB-M13^αLPS^ (1 × 10^12^ virions/mL) was measured, and the fluorescence of Sulfo-NHS-acetate-blocked M13^αLPS^ at the same concentration was subtracted as background, yielding the fluorescence attributable to conjugated PMB. Comparison to the standard curve gave an estimate of 18 ± 1.3 μg/mL of PMB in a sample containing 10^12^ virions/mL, or 3.1 ± 0.2 PMB molecules per copy of g8p, in reasonable agreement with the amino acid composition analysis.

Given three surface-accessible carboxylic acids on each of approximately 2,700 copies of pVIII per virion, up to ∼8,100 molecules of PMB could be theoretically conjugated per virion based on the number of reactive groups. However, since the phage surface is negatively charged while PMB is positively charged, increased loading would also cause colloidal instability as the particle charge is neutralized. Consistent with this expectation, conjugation to PMB altered the phage morphology, showing more compaction compared with unconjugated M13^αLPS^ (Figure S14). The PMB-conjugated M13^αLPS^, termed PMB-M13^αLPS^ hereafter, was filtered (0.22 μm) to remove the aggregates. Yield for the conjugation reaction after purification was approximately 20%. Another potential problem with high PMB loading is that nonspecific conjugation of PMB might interfere with binding to LPS, although prior literature indicates that nonspecific conjugation of small and large molecules (e.g., FITC, urease, neomycin) can occur without affecting phage binding to their targets.^55–57^ Regardless, these concerns regarding PMB loading were addressed by empirical optimization of the conjugation reaction to maximize the *in vitro* antibacterial effect of PMB-M13^αLPS^ (see below).

### Bactericidal Effect of PMB-M13^αLPS^ *In Vitro*

According to the recommended breakpoints for PMB (standard form: PMB sulfate salt) published by the United States Committee on Antimicrobial Susceptibility Testing in 2020,^58^ organisms with an MIC ≤ 2 μg/mL are considered susceptible, and those with an MIC ≥ 4 μg/mL are considered resistant.^59,60^ PMB-M13^αLPS^ concentrations were determined by UV absorbance. Based on the conversion factor from amino acid composition analysis of PMB-M13^αLPS^, we calculated the equivalent PMB concentrations and MIC values from PMB-M13^αLPS^ concentrations. MICs in liquid culture were determined by a standard broth microdilution assay, in which cells were cultured in the presence of varying concentrations of PMB or PMB-M13^αLPS^. We first tested the commonly accepted *E. coli* reference strain ATCC 25922 (acceptable MIC range for PMB: 0.25–2 μg/mL)^59,60^ and found the MIC for PMB (MIC_PMBSO4_) to be 2 μg/mL, validating the assay. Using PMB-M13^αLPS^, the MIC (MIC_PMB‑M13αLPS_) for the reference strain was found to be 5 x10^9^ virions/mL (equivalent to 0.054 μg/mL PMB), indicating that PMB-M13^αLPS^ indeed lowers the MIC (in this case, by 37-fold). We then determined MIC values for multiple Gram-negative species and strains.

In general, PMB-M13^αLPS^ effectively lowered the MIC by 1–2 orders of magnitude compared to PMB sulfate, for several different species and strains of Gram-negative bacteria, including clinical isolates (Table 2, Figure 2A). For *P. aeruginosa* strains, MIC_PMB‑M13αLPS_ was 19–74 -fold lower than MIC_PMBSO4_. For some strains that are resistant to PMB (MIC_PMBSO4_ above the breakpoint), namely *P. aeruginosa* PAKpmrB6 and *K. pneumoniae* clinical strain A, the MIC_PMB‑M13αLPS_ value was well within the susceptible range, confirming the greater potency of PMB-M13^αLPS^. Two strains were included that are extremely resistant to PMB (with MIC_PMBSO4_ > 100 times the breakpoint), namely *K. pneumoniae* clinical strain 326 and *B. cepacia* ATCC 25416. For these, the MIC_PMB‑M13αLPS_ value was higher than the tested concentration range, indicating a limitation for the potency of PMB-M13^αLPS^. To verify that PMB was the active component of PMB-M13^αLPS^, M13^αLPS^ (without PMB conjugation) was tested in the same MIC assay. As expected, M13^αLPS^ alone did not show bactericidal activity within the tested concentration range (Table 2). Certain clinical isolates, such as *E. coli* BAA 1161, had shown reduced M13^αLPS^ binding (Figure 1G), potentially due to strain-specific differences in LPS structure or accessibility or ssDNA isolation. However, this reduction did not translate into a higher MIC, indicating that delivery of PMB for cell-killing effect was robust to these differences.

**Table 2:** Minimum Inhibitory Concentration (MIC) of PMB Sulfate, PMB-M13^αLPS^, and M13^αLPS^ Determined *In Vitro* for Several Gram-Negative Organisms^a^

		MIC_PMBSO4_	MIC_PMB‑M13αLPS_	MIC_PMB‑M13αLPS_	MIC_PMBSO4_/MIC_PMB‑M13αLPS_	MIC_M13αLPS_
Species	Strain	(μg/mL)	μg/mL	virions/mL	(*n*-fold MIC reduction)	(virions/mL)
*E. coli*	ATCC 25922	2	0.054	5 × 10^9^	37	>5 × 10^12^
*E. coli*	ATCC BAA 1161	2	0.054	5 × 10^9^	37	>5 × 10^12^
*E. coli*	ATCC 700927	2	0.054	5 × 10^9^	37	>5 × 10^12^
*P. aeruginosa*	ATCC 25102	2	0.054	5 × 10^9^	37	>5 × 10^12^
*P. aeruginosa*	Clinical Strain E	2	0.027	2.5 × 10^9^	74	>5 × 10^12^
*P. aeruginosa*	PAKpmrB6	8*	0.43	4 × 10^10^	19	>5 × 10^12^
*P. aeruginosa*	GFP-PAO1	0.5	0.027	2.5 × 10^9^	19	>5 × 10^12^
*P. aeruginosa*	ATCC 27853	2	0.054	5 × 10^9^	37	n.d.
*P. aeruginosa*	LES 431	1	0.027	2.5 × 10^9^	37	n.d.
*P. aeruginosa*	AR Bank #0246	2	0.054	5 × 10^9^	37	n.d.
*P. aeruginosa*	AR Bank #0266	1	0.027	2.5 × 10^9^	37	n.d.
*K. quasipneumoniae*	ATCC 700603	2	0.054	5 × 10^9^	37	>5 × 10^12^
*K. pneumoniae*	Clinical Strain A	4*	0.107	1 × 10^10^	61	>5 × 10^12^
K. pneumoniae	Clinical Strain 326	>256*	>0.859	>8 × 10^10^		>5 × 10^12^
*A. baumannii*	ATCC 19606	2	0.054	5 × 10^9^	37	>5 × 10^12^
*B. cepacia*	ATCC 25416	>256*	>0.859	>8 × 10^10^		>5 × 10^12^

a For PMB, an MIC of 4 μg/mL or greater is defined as resistant (designated by *). *n*-fold reduction in MIC was calculated as the ratio MIC_PMBSO4_/MIC_PMB‑M13αLPS_ (both in units of μg/mL). n.d. = not determined. The experimental assay for MIC_PMBSO4_ and MIC_PMB‑M13αLPS_ is illustrated for three species in Figure 2A.

**Figure 2:**
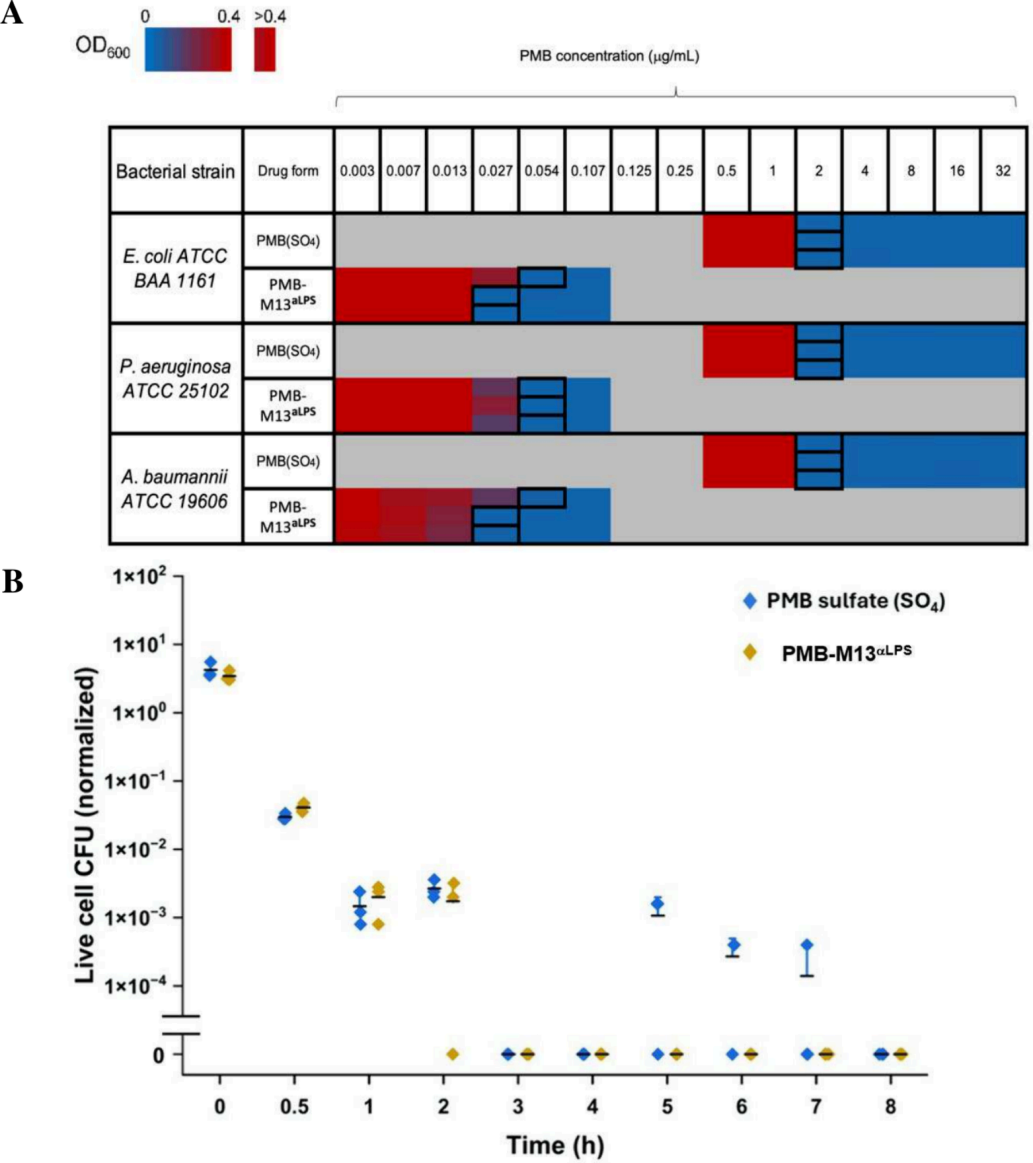
Antibacterial activity of PMB-M13^αLPS^ *in vitro*. (A) Microdilution assay to determine MICs, shown for three different bacterial species, as labeled. Heat map corresponds to bacterial cell density after incubation with various concentrations of PMB sulfate (SO_4_) or PMB-M13^αLPS^. The MIC value is the lowest concentration resulting in no growth, determined based on plate reader OD_600_ reading, and is labeled by the black box outline in each row. Triplicates are shown as 3 consecutive rows. If MIC measurements differed among triplicates, the highest concentration was taken to be the MIC. Gray indicates concentrations not tested. Raw data points are listed in Table S9. MICs are tabulated for 16 different bacterial species in Table 2. (B) Decrease in viable *E. coli* (ATCC BAA 1161) cells over time when incubated with PMB (blue) or PMB-M13^αLPS^ (yellow) at their respective MIC concentrations. Similarly rapid bacterial death is observed in both cases. The *y*-axis value is normalized by the number of cells (5 × 10^4^) estimated in each well at *t* = 0 h, based on OD_600_ (Table S2) (*n* = 3). Mean value (solid black dashed line) was calculated from three experimental replicates. Error bars (whisker in + direction) representing 1 standard deviation were calculated from experimental triplicates.

PMB is known to kill cells rapidly. PMB-M13^αLPS^ also killed bacteria, as verified by plating and colony counting to determine the minimum bactericidal concentrations (MBC). As observed for the MIC values, the MBC_PMB‑M13αLPS_ values were 19–74 lower than the MBC_PMBSO4_ values (Table S6). PMB-M13^αLPS^ also showed a rapid mechanism of action, with a >1,000-fold reduction of viable bacterial cells within 1 h in a time-kill kinetics assay (Figure 2B),^42^ comparable with PMB kinetics. Thus, PMB-M13^αLPS^ substantially lowers the MIC and MBC while maintaining the bactericidal effect and rapid kill time of PMB.

Biofilms are a major contributing factor for prolonged virulence in nosocomial infections, especially in *P. aeruginosa*,^61^ and can significantly limit antibiotic exposure of bacterial cells.^62^ We grew *P. aeruginosa* biofilms undisturbed for 3 days and then added various concentrations of PMB or PMB-M13^αLPS^ to assay bactericidal activity on cells in the biofilms. We found that both MBC_PMBSO4_ and MBC_PMB‑M13αLPS_ were higher for biofilm cells compared to planktonic cells by approximately 2 orders of magnitude. Regardless, a higher potency of PMB-M13^αLPS^ was also observed for the biofilm, with an MBC_PMB‑M13αLPS_ of 11 μg/mL (10^12^ virions/mL) compared to MBC_PMBSO4_ of 200 μg/mL, or a ∼20-fold improvement (Table S7).

### Stability of PMB-M13^αLPS^ in Solution

M13 phages have been reported to have a half-life of >120 days.^63^ PMB sulfate in 0.9% NaCl stored at 4 °C retains >75% activity after 7 days.^64,65^ We measured the stability of PMB-M13^αLPS^ using the MIC for *E. coli* (ATCC 25922) to assay retention of antibacterial activity. Five mL of PMB-M13^αLPS^ at 1 × 10^12^ virions/mL stored at 4 °C in 1 x PBS for >12 weeks showed no changed in MIC (Table S8). Thus, PMB-M13^αLPS^ retained full activity for >3 months of refrigerated storage.

### Cytotoxicity Testing of PMB-M13^αLPS^ on Mammalian Cells *In Vitro*

PMB-M13^αLPS^ was tested for hemolytic activity using sheep red blood cells. Materials demonstrating <2% loss of cell content are considered nonhemolytic.^66^ PMB-M13^αLPS^ was nonhemolytic at concentrations up to 8 x10^10^ virions/mL (32 times the MIC for *E. coli* strain 25922) (Figure 3A).

**Figure 3:**
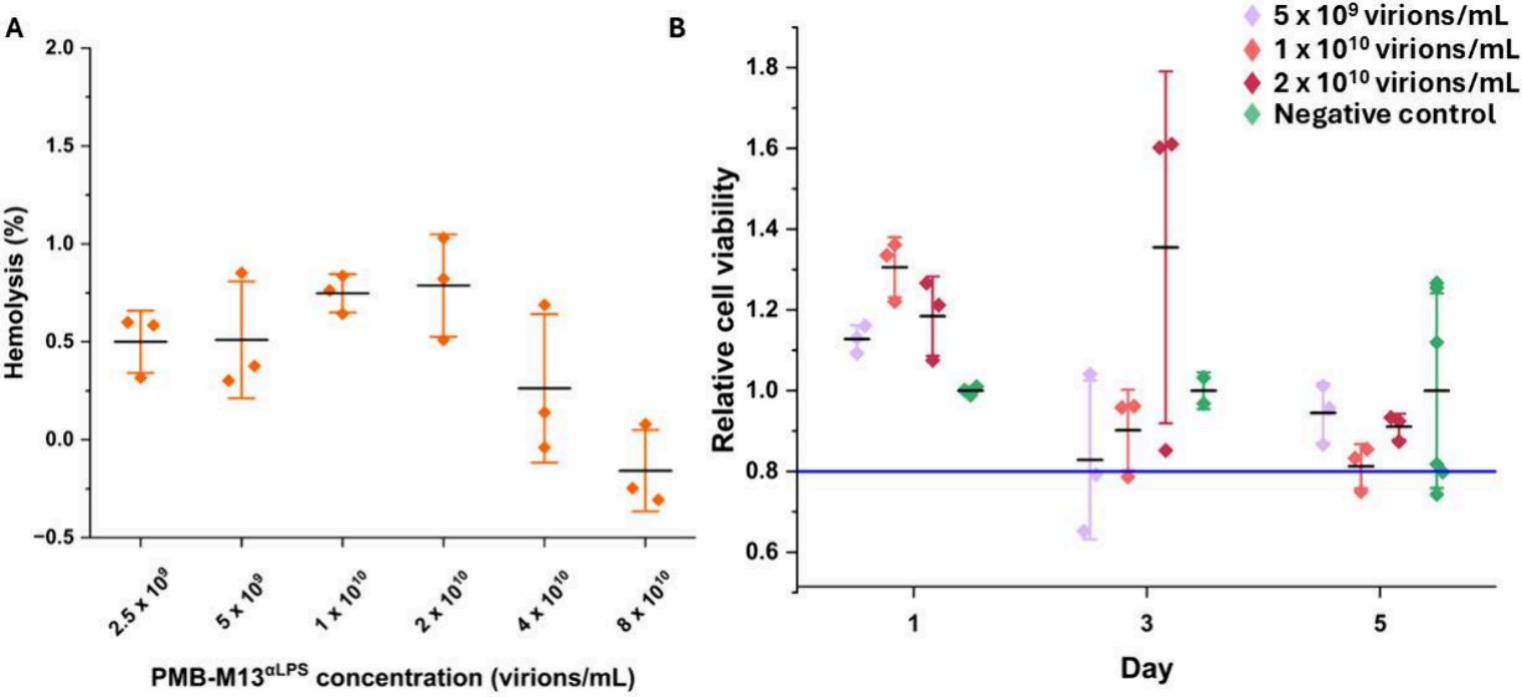
Cytotoxicity studies of PMB-M13. (A) Hemolysis assay of PMB-M13^αLPS^ on sheep red blood cells. Less than 2% of hemolysis is considered nonhemolytic, as observed for PMB-M13^αLPS^ at these concentrations.^66^ (B) HEK 293 cell viability, indicated by cell metabolic activity measured using the MTT assay, when incubated in growth media with and without PMB-M13^αLPS^. >80% cell viability is considered acceptable.^67^ Mean value (solid black dash) was calculated from three experimental replicates. Error bars (whiskers) representing 1 standard deviation were calculated from experimental triplicates.

Cell viability was tested using human embryonic kidney (HEK 293) cells cultured in media containing (0.5–2) × 10^10^ virions/mL of PMB-M13^αLPS^ (2–8 times the MIC for *E. coli* strain 25922). Fresh media containing the same concentration of PMB-M13^αLPS^ was added during passaging. Metabolic activity was assayed by MTT. All cultures exposed to PMB-M13^αLPS^ had >80% activity for the duration of the 5-day assay (Figure 3B) compared to negative control samples with no PMB-M13^αLPS^ supplemented, which is considered nontoxic.^67^ This result was verified by microscopy with Phalloidin/DAPI staining of cells cultured with PMB and PMB-M13^αLPS^ (Figure S15), which showed normal cell growth and morphology after 1, 3, and 5 days of culture with exposure to PMB-M13^αLPS^. The results indicated a lack of toxicity for mammalian cells *in vitro*.

### PMB-M13^αLPS^ Efficacy in a Multidrug-Resistant *P. aeruginosa* Pneumonia Model in Immunocompetent Mice

A mouse infection model was established based on a published model for lethal *P. aeruginosa* pneumonia.^68^ Infection was initiated by intranasal inoculation of 2.8 × 10^7^ CFU of *P. aeruginosa* strain AR Bank #0266, a clinical isolate strain that is resistant to multiple antibiotics including carbapenems and fluoroquinolones (Table S10), using female 7-week-old BALB/c mice. One h after infection, a 50 μL volume of each test article was formulated with 1 x PBS buffer and administered intranasally (N = 5). Test articles were vehicle control (1 x PBS), wild type (WT)-M13 phage (4 × 10^10^ and 8 × 10^10^ virions/dose), M13^αLPS^ (4 × 10^10^ and 8 × 10^10^ virions/dose), PMB sulfate (0.5, 80, and 160 μg/dose) and PMB-M13^αLPS^ (2 × 10^10^, 4 × 10^10^, and 8 × 10^10^ virions/dose, containing 0.2, 0.4, and 0.9 μg of PMB respectively). At 24 h after treatment, animals were sacrificed and lungs were homogenized to determine the viable bacteria (CFU count). The initial baseline CFU count was measured at one h post bacterial inoculation (without treatment). When 1x PBS, WT-M13, or M13^αLPS^ was administered, a 16-fold increase in bacterial CFU count was observed compared to baseline, reflecting bacterial growth during the 24-h period.

In comparison, a dose-dependent CFU reduction was observed in animals receiving PMB-M13^αLPS^ or PMB. Treatment with PMB-M13^αLPS^ at 4 × 10^10^ and 8 × 10^10^ virions/dose (equivalent to 0.4 and 0.9 μg of PMB, respectively) reduced the CFU count by 320-fold and 500-fold, respectively (*p* < 0.05 and *p* < 0.001, respectively) compared to 1 x PBS (Figure 4 **;** Table S11). The lowest dose of PMB-M13^αLPS^ (2 × 10^10^ virions) did not cause a significant reduction in CFU compared to 1x PBS. For PMB, the 80 and 160 μg doses resulted in 500-fold and 20,000-fold decrease in CFU (*p* < 0.001), while PMB at 0.5 μg dose did not cause a significant decrease in CFU, compared to 1x PBS. 160 μg of PMB corresponds to a previously determined maximum tolerated dose of PMB.^69^ Significantly, PMB-M13^αLPS^ containing 0.9 μg of PMB resulted in the same CFU reduction as PMB sulfate at 80 μg, indicating an ∼90-fold increase in drug potency *in vivo*.

**Figure 4:**
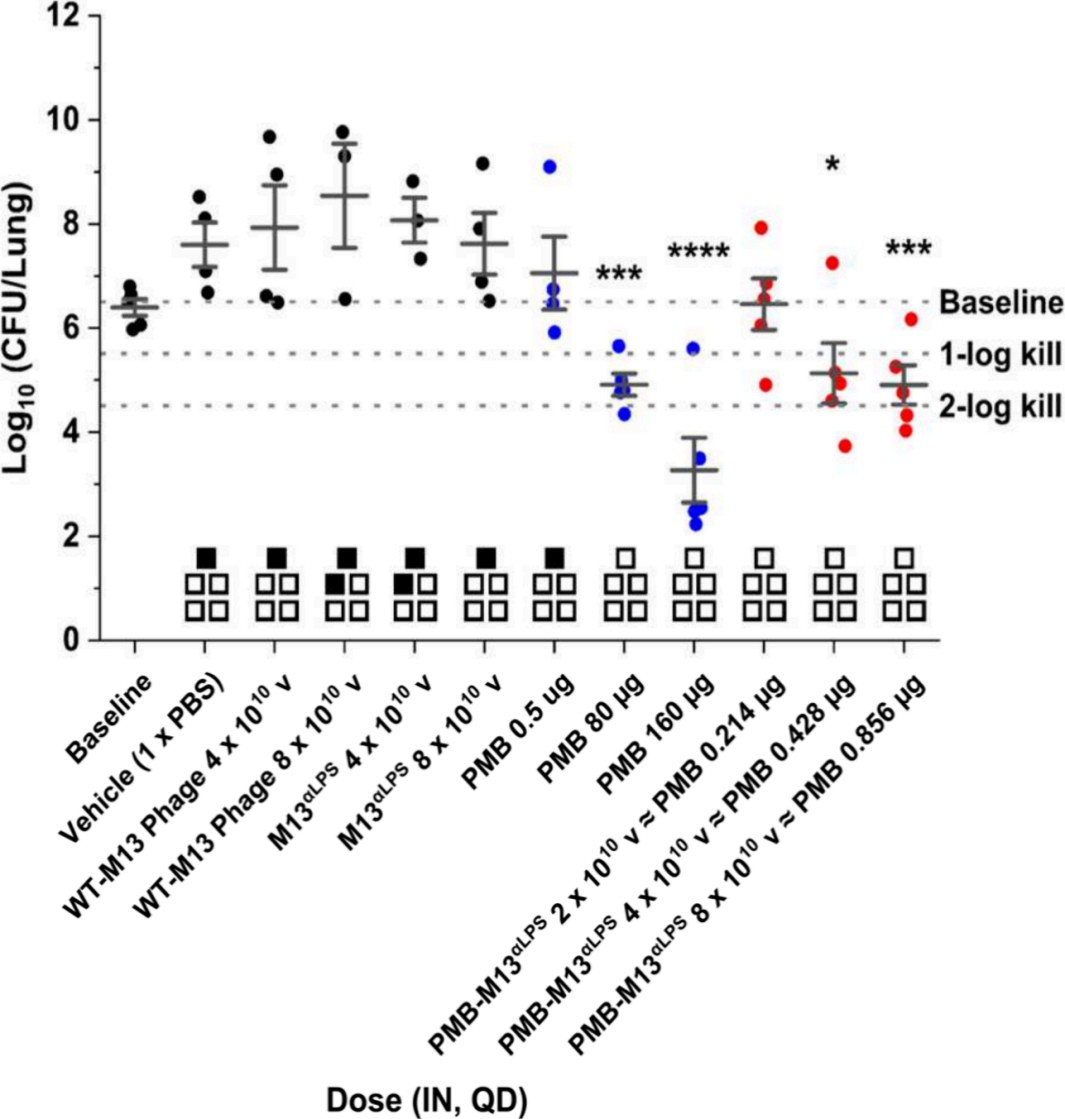
Efficacy of PMB-M13^αLPS^ in the immunocompetent lung infection model with multidrug-resistant *P. aeruginosa*. Test animals were intranasally (IN) inoculated with 2.8 × 10^7^ CFU of *P. aeruginosa* strain AR Bank #0266. 1 x PBS, wild-type M13 phage (WT-M13), M13^αLPS^, PMB sulfate (blue), or PMB-M13^αLPS^ (red) was administered IN at 1 h post infection. Animals were sacrificed at 1 or 25 h post infection and the lung tissues harvested. The bacterial counts (CFU/lung) of lung tissue homogenates were measured. Each point represents one individual animal. Baseline, 1-log kill, and 2-log kill levels are illustrated with respect to the CFU counts at 1 h post infection, without treatment. Control treatments gave increased CFU counts over baseline due to bacterial proliferation until 25 h. Statistical significance of comparisons to the 1 x PBS control group was determined by one-way ANOVA and Dunnett’s test. (*) means *p* < 0.05; (***) means *p* < 0.001; (****) means *p* < 0.0001. |d| = 2.86605, 95% CI, degrees of freedom = 36, *p* < 0.0001 for PMB (160 μg), *p* = 0.0085 for PMB (80 μg), *p* = 0.0087 for PMB-M13^αLPS^ (8E10 v), and *p* = 0.0188 for PMB-M13^αLPS^ (4E10 v). The boxes above the *x*-axis indicate death or survival of the five animals within each group: solid black box = animal died before 25 h and empty box = animal alive at 25 h. QD: once per day (single dose). *N* = 5 for each group, including animals that died. Error bars represent standard deviation calculated from available experimental replicates within each group, depending on animal attrition prior to time of sacrifice at 25 h post infection.

Animal mortality (death before 25 h) was 20–40% for groups given PBS, WT-M13, or M13^αLPS^, consistent with an untreated infection from the bacterial inoculum. The lowest PMB dose also resulted in 20% mortality, consistent with ineffective bacterial killing as determined by CFU counts, while the medium and high PMB doses resulted in no deaths. No animal deaths were observed in the three groups treated by low, medium, or high doses of PMB-M13^αLPS^ (Figure 4, Figure S16). These findings demonstrate that PMB-M13^αLPS^ is safe and highly effective at killing a multidrug-resistant strain of *P. aeruginosa* *in vivo*, resulting in the survival of all infected mice.

### PMB-M13^αLPS^ Efficacy in a Mouse Model of Blinding *P. aeruginosa* Corneal Infection

We next used a well-established murine model of *P. aeruginosa* keratitis to test the efficacy of PMB-M13^αLPS^ treatment. The corneal epithelium of C57Bl/6 mice were abraded, and 5 × 10^4^ CFU of a *P. aeruginosa* strain expressing Green Fluorescent Protein (GFP), PAO1-GFP, was added topically to the ocular surface. Use of a GFP-expressing strain allows fluorescence quantitation of bacteria in addition to CFU counts. Mice were treated with 1 × 10^10^ virions/dose of M13^αLPS^ or wild-type M13 phage, PMB sulfate at 0.5 and 0.25 μg/dose, or PMB-M13^αLPS^ at 6.25 × 10^8^ to 2.5 × 10^9^ virions/dose (containing 0.007 to 0.027 μg of PMB), by topical application of 10 μL at 2-, 6-, and 24 h postinfection. 48 h postinfection, animals were euthanized, corneal disease and GFP were quantified by image analysis, and eyes were homogenized for CFU counts.

Corneas infected with GFP-PAO1 develop pronounced opacification that is associated with GFP expressing bacteria (Figure 5A, Figure S17). There was no difference in corneal disease or total GFP in mice treated with M13^αLPS^ or M13 phage compared with control mice given topical PBS. In contrast, mice treated with 0.5 or 0.25 μg of PMB alone exhibited clear corneas with no GFP, indicating bacterial killing. Importantly, corneas treated with PMB-M13^αLPS^ containing 10 to 100-fold less PMB were also clear with little detectable GFP.

**Figure 5:**
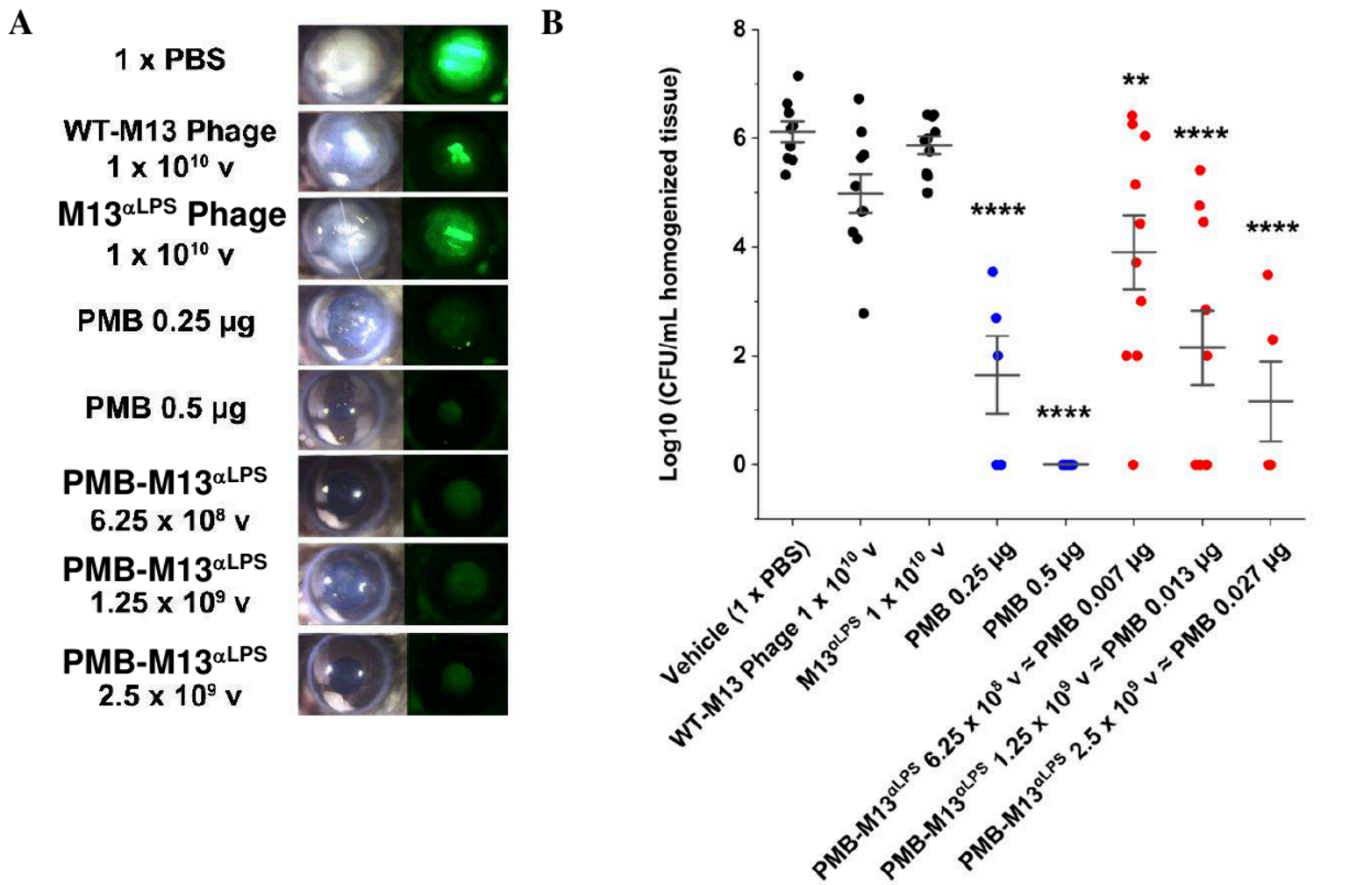
Efficacy of PMB-M13 in a mouse keratitis model with *P. aeruginosa* infection. Animals were subject to corneal scratch and inoculated with 5 × 10^4^ CFU of the fluorescent *P. aeruginosa* strain PAO1-GFP. M13^αLPS^, WT-M13 phage, PMB sulfate, or PMB-M13^αLPS^ was administered topically as the treatment. Animals were euthanized at 48 h postinfection. (A) Representative bright-field and fluorescent images of infected eyes in each group. Additional images and quantification are given in Figure S17. (B) CFU was counted from harvested eye tissue. Statistical significance of comparisons to the PBS control were determined by one-way ANOVA and Tukey’s test. (**) means *p* < 0.01; (****) means *p* < 0.0001. v = virions. |d| = 2.69698, 95% CI, degrees of freedom = 61, *p* < 0.0001 for PMB (50 μg/mL) and PMB-M13^αLPS^ (2.5E9 (v), *p* = 0.0218 for PMB (25 μg/mL), *p* = 0.011 for PMB-M13^αLPS^ (1.25E9v), and *p* = 0.0279 for PMB-M13^αLPS^ (6.25E8 (v). Mean value (solid black dashed line) and error bars (whiskers) representing 1 standard deviation were calculated from experimental replicates.

Analysis of viable bacteria by CFU counts reflected this clinical outcome. Compared to the PBS control (N = 9; 1 animal died prior to data collection), treatment with M13^αLPS^ (N = 10) or wild-type M13 phage (N = 10) did not significantly reduce CFU (Figure 5B; Table S12). In comparison, dose-dependent CFU reductions were observed following treatment with PMB-M13^αLPS^ or PMB. All doses of PMB-M13^αLPS^ (6.25 × 10^8^ (N = 10), 1.25 × 10^9^ (N = 10), and 2.5 × 10^9^ (N = 5) virions/dose, containing 0.007, 0.013, and 0.027 μg of PMB per dose, respectively) yielded significant >2-log CFU reductions relative to 1 x PBS (*p* < 0.01, *p* < 0.0001, and *p* < 0.0001 respectively). Similarly, topical PMB at 0.5 (N = 10) and 0.25 (N = 5) μg/dose resulted in significant >2-log reductions in CFU compared to 1x PBS (*p* < 0.0001 for both). PMB-M13^αLPS^ containing 0.013–0.027 μg of PMB resulted in a significant reduction in CFU (>4-log_10_ decrease compared to PBS) similar to 0.25 μg of PMB sulfate, indicating a ∼10–20-fold increase in drug potency. These findings demonstrate that PMB-M13^αLPS^ was effective at killing *P. aeruginosa* and blocking the corneal opacification associated with visual impairment and blindness.

### Toxicity Testing of PMB-M13^αLPS^ in Mice

Unlike PMB, M13 phage has been shown previously to demonstrate no toxicity *in vivo*.^70,71^ To test PMB-M13^αLPS^, male mice were subjected to daily IV tail vein injections of PMB-M13^αLPS^ for 1 week, at daily doses of 6.9 × 10^9^, 1.4 × 10^10^, 2.7 × 10^10^, and 1.6 × 10^11^ virions. Doses administered in the mouse infection models fell within or below this concentration range of PMB-M13^αLPS^. Given the PMB loading (1.1 × 10^–11^ μg of PMB/virion) and a typical animal weight of 27 g, these doses are equivalent to 3–64 mg of PMB per kg of body weight per dose. Control animals were untreated or injected with 1x PBS or PMB sulfate solution (2.5, 21, or 72 mg/kg).^72–74^ Blood samples taken 24 h after the last injection showed no impairment of liver or kidney function, as assessed by biomarkers (Figure 6, Figure S18). Blinded histological analysis also showed no observable toxicity at these PMB-M13^αLPS^ concentrations for kidney, liver, and spleen samples (Supplementary Data Files, Table S13). All animals receiving PMB-M13^αLPS^ survived with no observable indication of sickness based on lethargy, fur condition, posture, or body temperature. In contrast, a single dose of PMB at 72 mg/kg led to death in all animals (3/3) on day 1. The lack of toxicity *in vivo* for PMB-M13^αLPS^ supported the *in vitro* cytotoxicity results and supported the premise that phage-based delivery should widen the therapeutic window of PMB.

**Figure 6:**
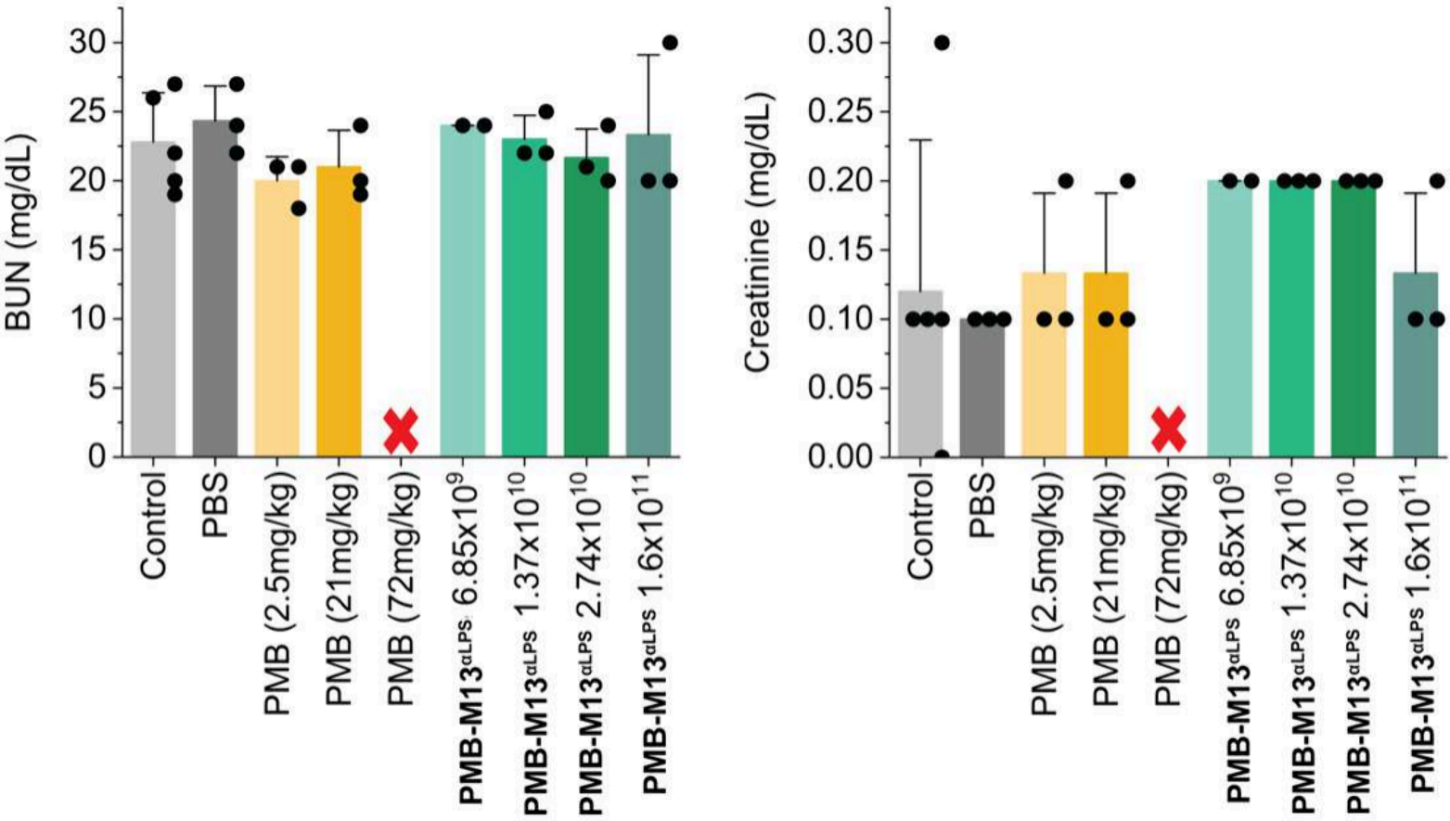
Kidney function blood biomarkers, blood urea nitrogen (BUN) and creatinine, at the end point after 7-day toxicity studies of PMB-M13^αLPS^. Animals in treated groups were injected with 100 μL of test materials (PMB-M13^αLPS^, PMB, or 1x PBS) through IV tail vein injection daily for 7 consecutive days. Biomarker levels did not indicate liver or kidney injury from PMB-M13^αLPS^. However, all animals receiving 72 mg/kg body weight of PMB (*N* = 3) died after the first dose on day 1 (indicated by red ‘x’ in figure). *N* = 3 per group. Error bars (whiskers in plus direction) representing 1 standard deviation were calculated from experimental replicates

### Biodistribution of PMB-M13^αLPS^ in Mice

To characterize PMB-M13^αLPS^ biodistribution, PMB-M13^αLPS^ was radioactively labeled with ^89^Zr, using the chelator deferoxamine (DFO), and 5 × 10^10^ virions (containing 100 μCi radioactivity) were injected into male mice. The mice were separated into 2 groups, with one group also having 1 × 10^7^ CFU of *E. coli* BAA 1161 administered IV through tail vein injection 30 min before PMB-M13^αLPS^ injection, to investigate whether bloodstream *E. coli* would impact biodistribution of PMB-M13^αLPS^. At time points of 10 min, 30 min, and 1, 2, 4, 6, 24, 48, and 72 h after injection, the mice were imaged with μPET coregistered with μCT and the percentage of injected dose (ID) per volume (cc) was quantified from images after accounting for radioactive decay. PMB-M13^αLPS^ was found to be distributed to all organs tested within 1 h and remained present for at least 6 h, with an overall elimination half-life of approximately 1 day (Figure 7, Figure S19). The rate of PMB-M13^αLPS^ systemic clearance decreased after 24 h, with ∼50% injected dose remaining in the system after 72 h, which did not depend on the bacterial injection. The ^89^Zr-labeled PMB-M13^αLPS^ was substantially distributed to kidneys, liver, and spleen, each with approximately 8–25% of the injected dose/cc after 24 h. Bacteremia affected the distribution among organs moderately, with increased PMB-M13^αLPS^ observed in the lungs and muscle and decreased PMB-M13^αLPS^ in the kidneys and spleen, with effects generally within 5% change in ID/cc. Overall, distribution from the circulation to major organs occurred quickly (within 1 h), and PMB-M13^αLPS^ stayed within the body for a significant time (24 h).

**Figure 7:**
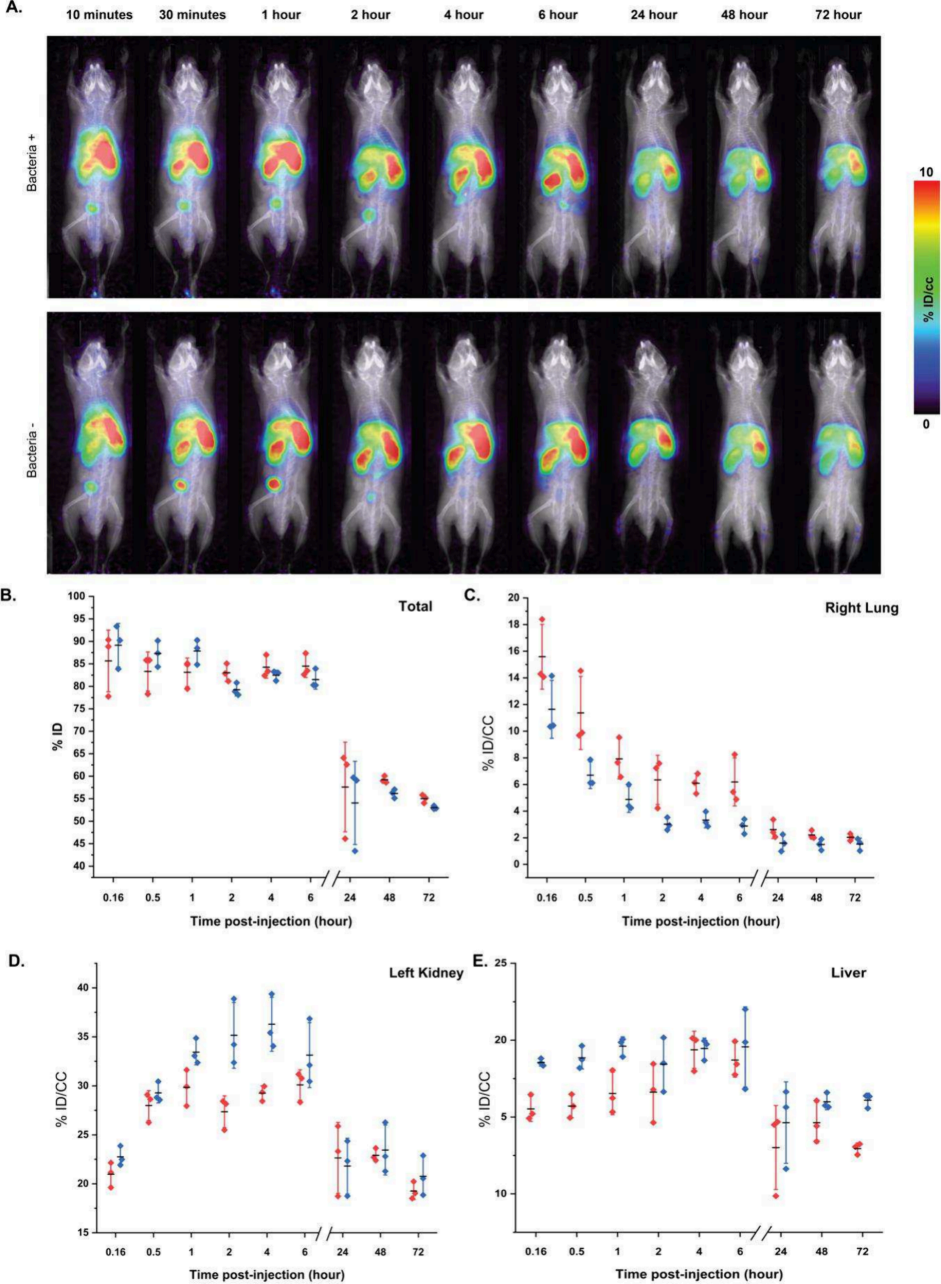
Biodistribution of ^89^Zr-labeled PMB-M13^αLPS^. Representative μPET/CT images across the 72-h time period are shown in (A), for one animal in the group with (top row) or without (bottom row) bacterial injection. The distribution into different organs and tissues over time were measured for (B) the whole animal, (C) right lung, (D) left kidney, and (**E**) liver over a 72 h time period. Mice were injected with PMB-M13^αLPS^ alone (blue) or were preinjected with 1 × 10^7^ cfu *E. coli* ATCC BAA 1161 followed by injection of PMB-M13^αLPS^ (red). Error bars (whiskers) show standard deviation (*N* = 3). Data were quantified from μPET images. Signals are measured as percentages of the injected dose (ID) per volume (cc). Mean value (solid black dash) was calculated from three experimental replicates.

## Discussion

As the discovery pipeline for new antibiotics has dwindled in the past few decades, generalizable methods to increase the potency of existing antimicrobial molecules are increasingly important to salvage antibiotics having low potency or high toxicity, such as AMPs. Here we demonstrate how phage-AMP conjugates could fulfill this role. Phage M13 has a large surface area and thousands of sites for chemical conjugation. Each virion of PMB-M13^αLPS^ carried thousands of PMB molecules, delivering them simultaneously to a single bacterium. Display of an anti-LPS scFab domain on the phage conferred the ability to bind and kill a wide range of Gram-negative microbes through conjugated PMB molecules, including the Gram-negative ESKAPE pathogens. *In vitro*, PMB-M13^αLPS^ decreased the amount of PMB required to kill the organisms by 1–2 orders of magnitude. Due to the increased potency, some strains that are resistant to PMB were susceptible to PMB-M13^αLPS^. For example, *P. aeruginosa* PAKpmrB6 expresses a modification to lipid A^75^ that renders this strain resistant to PMB (MIC = 8 μg/mL). However, PMB-M13^αLPS^ was able to kill this strain (MIC = 0.43 μg/mL), as the phage targets the core oligosaccharide of LPS. At the same time, strains that are extremely resistant to PMB (MIC > 256 μg/mL) were also resistant to PMB-M13^αLPS^, consistent with the upper limit of potency improvement (2 orders of magnitude) observed here. Regardless, the *in vitro* results validated the main advantage of the phage-AMP conjugate design, namely, the potential to greatly reduce dosage.

For *in vivo* testing, we focused on the ESKAPE pathogen *P. aeruginosa*, which is a clinically important pathogen that causes a variety of infections. *P. aeruginosa* is the most common Gram-negative organism causing hospital-acquired and ventilator-associated pneumonia^76^ and frequently exhibits a high level of resistance to multiple antibiotics, requiring increased dosing.^77,78^ *P. aeruginosa* lung infections were established in mice using a multidrug-resistant strain from the CDC & FDA Antimicrobial Resistance Isolate Bank, and then treated with a single intranasal dose of PMB-M13^αLPS^. PMB-M13^αLPS^ treatment resulted in >2-log_10_ decrease in bacterial CFU compared to the negative control treatments (e.g., wild-type M13), which is considered to be a potentially translatable antibacterial effect.^79^ Furthermore, the results validated the phage-AMP conjugate design: the high potency observed *in vitro* carried over *in vivo*, as PMB-M13^αLPS^ showed efficacy in reducing bacterial CFUs at <1 μg total dose per mouse, compared to 80 μg total dose needed of PMB sulfate, or approximately 2 orders of magnitude (∼90-fold) increase in potency.

*P. aeruginosa* is also responsible for 6%–39% of bacterial keratitis (corneal infection) cases in the US,^80^ as illustrated by a nationwide outbreak from artificial tears contaminated by extensively drug-resistant *P. aeruginosa* in 2023.^81–83^ *P. aeruginosa* keratitis was established in mice and treated topically with PMB-M13^αLPS^. As with the lung infection model, effective treatment and increased potency (∼10–20-fold for the keratitis model) were observed *in vivo*.

Intracellular accumulation of PMB and subsequent apoptosis of kidney proximal tubular cells is proposed to be the mechanism of PMB-induced nephrotoxicity.^84^ Toxicity, including hemolysis, is a general challenge for AMPs.^85^ For PMB-M13^αLPS^, no cytotoxicity or hemolysis was observed *in vitro* or during *in vivo* studies with intranasal or ophthalmic treatment. To test whether toxicity might be observed with intravenous injection, mice were injected with daily doses for 1 week, with each daily dose exceeding the amounts used in either mouse infection model. Nephrotoxicity and other toxic effects were not observed in serum biomarkers or by histology. Thus, PMB-M13^αLPS^ appears to be highly potent, efficacious, and nontoxic.

In contrast to phage therapy, the phage-AMP conjugate does not rely on phage infection and gene expression or lysis of bacterial cells for efficacy, and indeed phage alone did not show detectable antimicrobial activity *in vitro* or *in vivo*.^20,86^ Instead, the phage serves as an engineerable delivery vehicle for the active molecule (PMB). Only binding, not infectiousness, is required for antimicrobial activity. Without the need to maintain infectiousness, the phage could be engineered for broad-range activity by targeting a widespread receptor, the core antigen of LPS. Indeed, this approach enabled binding to a wide range of Gram-negative strains, including clinical isolates. This sidesteps a major challenge of phage therapy, namely, the narrow host range of most phages, which typically necessitates a lengthy personalized process to identify a phage for each clinical isolate and thus is not suited to acute infections. Isolating, characterizing, and manufacturing clinically acceptable phages takes 28–386 days.^87^ On the other hand, a conjugate like PMB-M13^αLPS^ would not be personalized and could potentially be applied in acute or resource-limited settings.

The general concept of increasing potency by targeted delivery could also be executed, in principle, with antibodies. Indeed, antibody-drug conjugates (ADCs) are a current growth area for the treatment of cancer. In principle, phages have a larger surface area compared to antibodies and a correspondingly greater payload potential. The payload capacity in our system was on the order of 10^3^–10^4^ PMB molecules per phage, compared to typical drug:antibody ratios of <10. In addition, phages deliver cargo in a spatial arrangement that differs from antibody-mediated delivery, which may be advantageous depending on the antimicrobial mechanism. Phage production using *E. coli* is also potentially low-cost compared to monoclonal antibody production. ADCs and phage-drug conjugates may both be seen as part of a continuum of delivery agents of varying properties, particularly, payload capacity.

PMB-M13^αLPS^ showed biodistribution to several major organs with IV injection, in comparison to PMB, which distributes heavily to the kidney, leading to renal tubular cell apoptosis and nephrotoxicity.^12,88^ M13 phage, like most nanoparticles of this size, is known to be more heavily distributed to the liver and spleen compared to kidney.^89^ The biodistribution of PMB-M13^αLPS^ showed a mixed pattern, consistent with a lack of observed toxicity. At the same time, localized administration, as used for the *in vivo* studies here, may be clinically preferable when possible (e.g., nebulization for phage administration to *P. aeruginosa* lung infections).^90,91^

Potential clinical concerns for PMB-M13^αLPS^ include the development of bacterial resistance, immunogenicity, and collateral microbiome damage. While some mechanisms of bacterial resistance, such as CRISPR-Cas systems and drug efflux pumps, would not apply to phage-AMP conjugates, spontaneous mutation of LPS altering the core antigen is a potential source of resistance. In addition, the expression of bacterial capsules may compromise access to LPS in some strains; in such cases, engineering to target capsular antigens may be considered to counter this problem. With respect to immunogenicity, antiphage antibodies can develop over several weeks of continued treatment, so phage-AMP conjugate treatment might be better suited to shorter regimens.^92,93^ Interestingly, drug-conjugated M13 causes ∼4-fold lower antiphage antibodies compared to unconjugated M13 phages *in vivo*.^70^ Immunogenicity and other interactions with the mammalian host are likely to depend on the specific phage (and drug) used in the conjugate. While M13 is being considered for biomedical applications, use of other phages may change properties such as cellular uptake, biofilm formation, or adherence to host surfaces.^94–99^ While PMB-M13^αLPS^ would not be selective for Gram-negative pathogens vs commensal organisms, localized delivery (e.g., to the lung) could mitigate potential microbiome damage. In addition, the majority of beneficial microflora are Gram-positive species,^100^ which would not be targeted by the anti-LPS design. Additional studies would be needed to define these aspects of the clinical potential of PMB-M13^αLPS^.

## Conclusion

In this proof-of-concept study, a phage-antimicrobial peptide conjugate, PMB-M13^αLPS^, was shown to be highly potent and effective against Gram-negative infection. Potency, and therefore therapeutic index, was increased 90-fold in a *P. aeruginosa* pneumonia model and 20-fold in a *P. aeruginosa* keratitis model *in vivo*. No toxicity was observed by serum biomarkers or histology after 1 week of daily intravenous dosing of PMB-M13^αLPS^. To our knowledge, this is the first report of a phage-antibiotic conjugate treatment demonstrating efficacy *in vivo*. This strategy for increasing therapeutic index appears useful for AMPs, as shown here, and would be potentially generalizable to other cargo with appropriate chemical cross-linking.^25^ Given the modular design, the delivery strategy might also be adapted to target specific pathogen species or Gram-positive organisms by engineering phage protein pIII to bind the desired targets. As recent outbreaks of multidrug-resistant organisms in community and hospital settings emphasize the need, synthetic nonlytic phage delivery may provide a route toward additional antimicrobial agents through salvage of low-potency or toxic molecules. This salvage approach may enable a higher rate of clinical translation compared to that of de novo antibiotic discovery. The phage-drug conjugate platform may also be applicable to diseases other than bacterial infections, such as cancer, in which precision targeting to increase the therapeutic index is desired.

## Supplementary Material



## Data Availability

All data and code presented in this paper are shown in the main or supplementary text, tables and figures. Histological images will be given on the DRYAD database at DOI: 10.5061/dryad.47d7wm3mw. File format.svs can be viewed using freely accessible software (Aperio Imagescope developed by Leica Biosystems (Wetzar, Germany)).

## Notes

Structures of polymyxin B and LPS, design of anti-LPS domain, UV–vis absorbance spectrum, DNA gels of PCR products, TEM of phage and bacteria, qPCR and ELISA binding data, amino acid analysis data and code, standard curves, cytocompatibility microscopy images, corneal infection data, biomarker data, biodistribution data, primer sequences, MBC and MIC data, antibiotic resistance characterization, and CFU data from *in vivo* study (PDF)
