# Supplementary material for: A Synthetic Phage-Peptide Conjugate as a Potent Antibacterial Agent for Pseudomonas aeruginosa Infections
Source: ACS Cent Sci. 2025 Jul 31;11(9):1715–35. doi: 10.1021/acscentsci.5c00562 (PMC12464783; doi:10.1021/acscentsci.5c00562)
Supplement: Supplementary file 1 [file oc5c00562_si_001.pdf]

Supporting Information

A Synthetic Phage-peptide Conjugate as a Potent Antibacterial Agent for *Pseudomonas aeruginosa* Infections

Yanxi Yang[1,2], Shelby Vexler[1,2], Maria C. Jordan[3], Serena Abbondante[4], Dayeon Kang[1,2], Huan Peng[5], Michaela Marshall[4], Bita V. Naini[6], Saumya Jain[1], Yei-Chen Lai[7], Nasim Annabi[1], Kenneth P. Roos[3], Eric Pearlman[4], Irene A. Chen[1,2]*

1. Department of Chemical and Biomolecular Engineering, University of California, Los Angeles, CA 90095, USA
2. Department of Chemistry and Biochemistry, University of California, Los Angeles, CA 90095, USA
3. Department of Physiology, David Geffen School of Medicine, University of California, Los Angeles, CA 90095, USA
4. Department of Ophthalmology, School of Medicine, University of California, Irvine, CA 92697, USA
5. Cellular Signaling Laboratory, International Research Center for Sensory Biology and Technology of MOST, Key Laboratory of Molecular Biophysics of MOE, College of Life Science and Technology, Huazhong University of Science and Technology, 430074 Wuhan, Hubei, China
6. Department of Pathology & Laboratory Medicine, David Geffen School of Medicine, University of California, Los Angeles, CA 90024, USA
7. Department of Chemistry, National Chung Hsing University, Taichung City 402, Taiwan

*Correspondence: ireneachen@ucla.edu

Supporting Figures

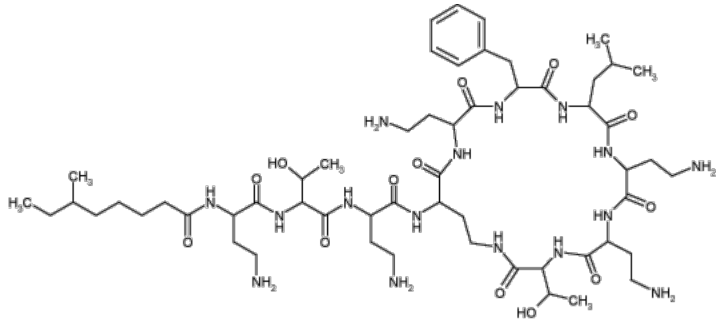

Figure S1. Structure of Polymyxin B. Polymyxin B is composed of a fatty acyl chain, a linear tripeptide (1L-Dab, 2L-Thr, 3L-Dab) domain and a positively charged heptapeptide ring (4L-Dab, 5L-Dab, 6D-Phe, 7L-Leu, 8L-Dab, 9L-Dab, 10L-Thr). Polymyxin B sulfate is sold as a mixture of two components of similar structures, polymyxin B1 and B2, which differ from each other at the fatty acid domain(*98*).

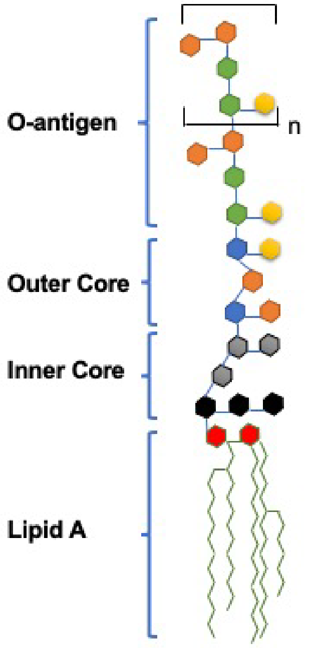

Figure S2. Schematic illustration of lipopolysaccharide (LPS). LPS is composed of lipid A (integrated into the outer membrane), core oligosaccharide (composed of inner core and outer core), and an O-antigen (facing the environment). The polygons represent sugars as follows: Black, 3-deoxy- α -D-mannooctulosonic acid; Gray, Heptulose; Blue, Glucose; Orange, Galactose; Green, Galactosamine; Yellow, Glucosamine; Red, β-glucosamine-(1→6)-glucosamine-1-phosphate(*99*).

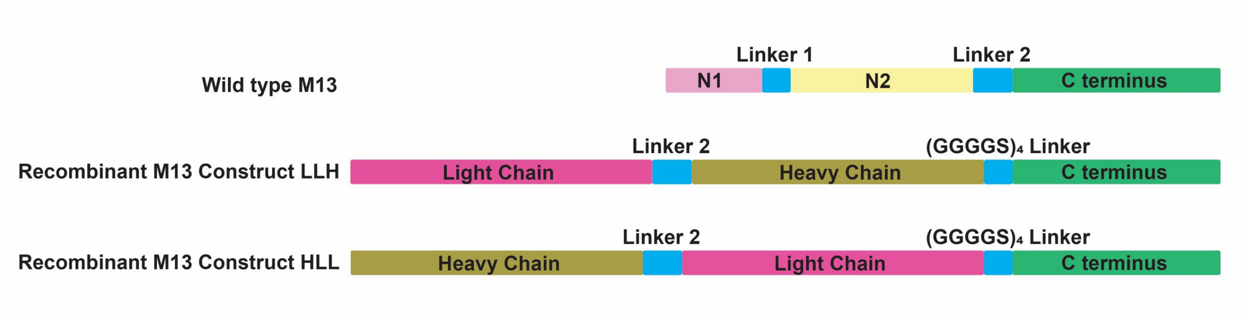

Figure S3. Design of two recombinant M13 derivatives with modified receptor-binding proteins (pIII). Colored rectangles indicate protein domains as labeled. In the recombinants, the C-terminal domain and Linker 2 on the phagemid vector were retained, while the N2, Linker 1, and N1 domains were replaced by single-chain fragment antigen binding regions of antibody WN1 222-5. The two constructs LLH and HLL differ from each other by the order of the heavy chain and light chain.

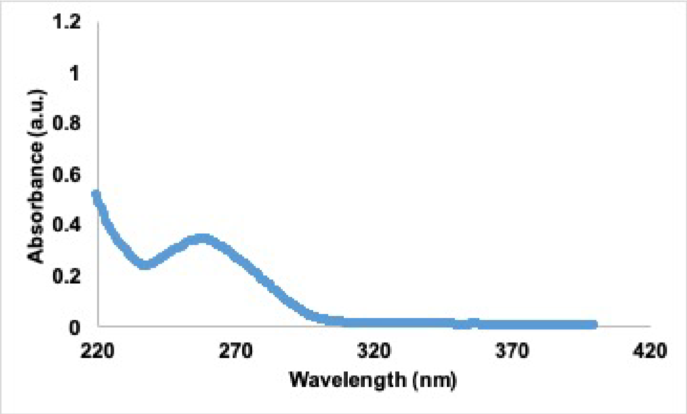

Figure S4. Representative spectrophotometry for quantification of recombinant M13 phages by absorbance. The purified phages in 1x PBS buffer gave a peak between 260 nm and 280 nm, consistent with expectation(*46*).

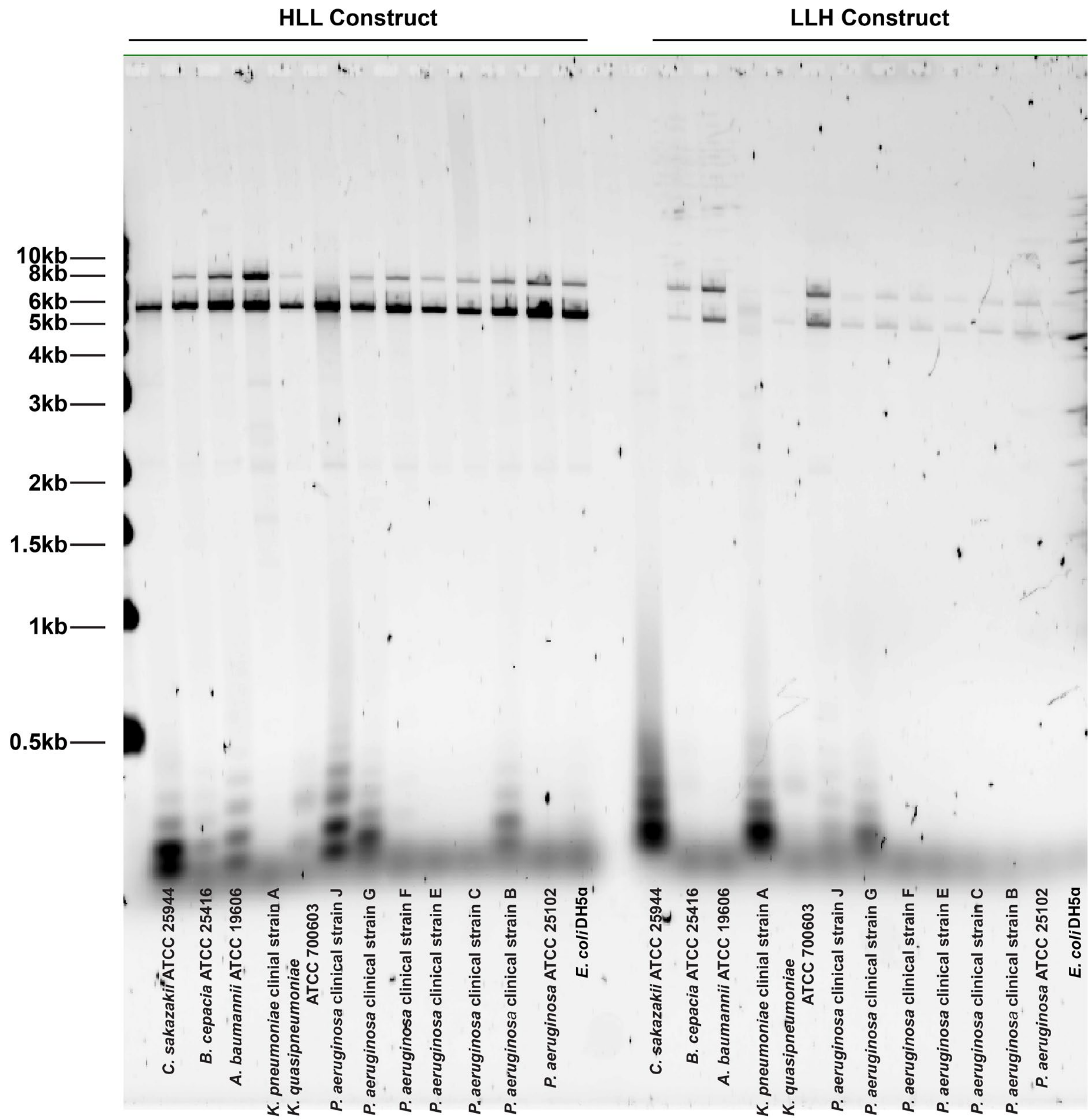

Figure S5. Binding of HLL vs. LLH phages. After incubation of bacterial cells with phages for binding, phage pull-down, and PCR, an agarose gel was used to analyze PCR products after 15 cycles using primer pair OrfiF1longForward and OriF1longReverse. Two bands are visible. The phagemid vector is expected at 5615 bp and the helper phage is expected at 7391 bp. At the far left is a DNA ladder with molecular weight markers indicated. The bacterial species and strain for each lane is given at the bottom; the phage construct is given at the top. In general, HLL samples gave more PCR products, indicating stronger binding and supporting qPCR results.

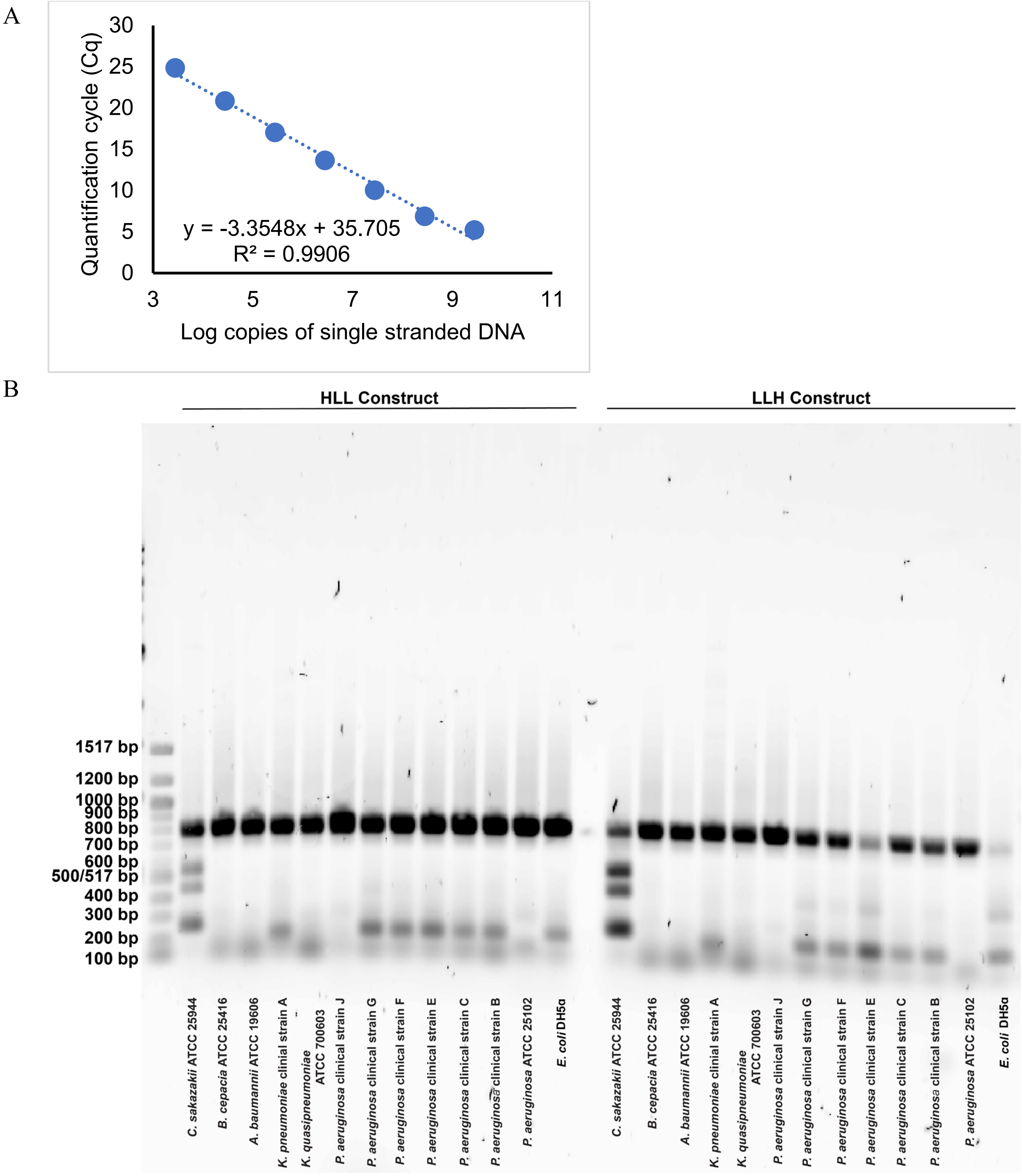

Figure S6. qPCR of HLL and LLH binding to bacterial cells using primer pair qPCR-upstream and qPCR-downstream and 3 µL of miniprepped sample in a 10 µL reaction volume. (A) Standard curve of the Cq value vs. amount of phagemid vector pADL10b (concentration determined by UV spectrometry). (B) Agarose gel of qPCR end products after 40 cycles. The expected product is 780 bp in length, matching the observed bands.

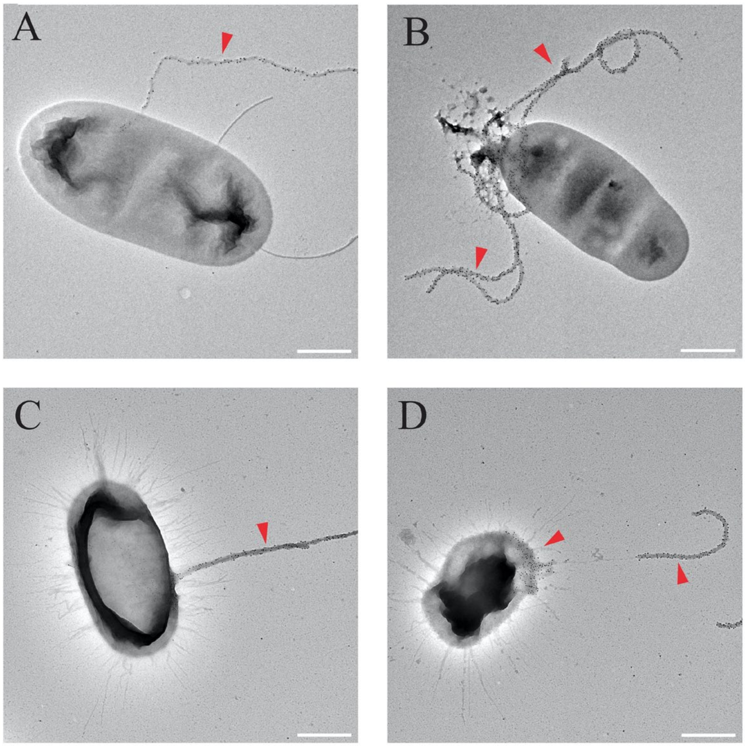

Figure S7. Transmission electron microscopy (TEM; negative stain) images are shown. M13$^{\alpha LPS}$ was labeled using a mouse monoclonal anti-pVIII primary antibody, followed by gold nanoparticles (dark spheres) coated with donkey anti-mouse secondary antibody, allowing phages to be easily identified (red darts). Gold-labeled M13$^{\alpha LPS}$ is incubated with (A) *E. coli* BAA 1161; (B) *P. aeruginosa* strain PAKpmrB6; (C) *K. pneumoniae* strain clinical strain A; and (D) *B. cepacia* ATCC 25416. Note that phage width is increased by the labeling reagents. Scale bars = 500 nm.

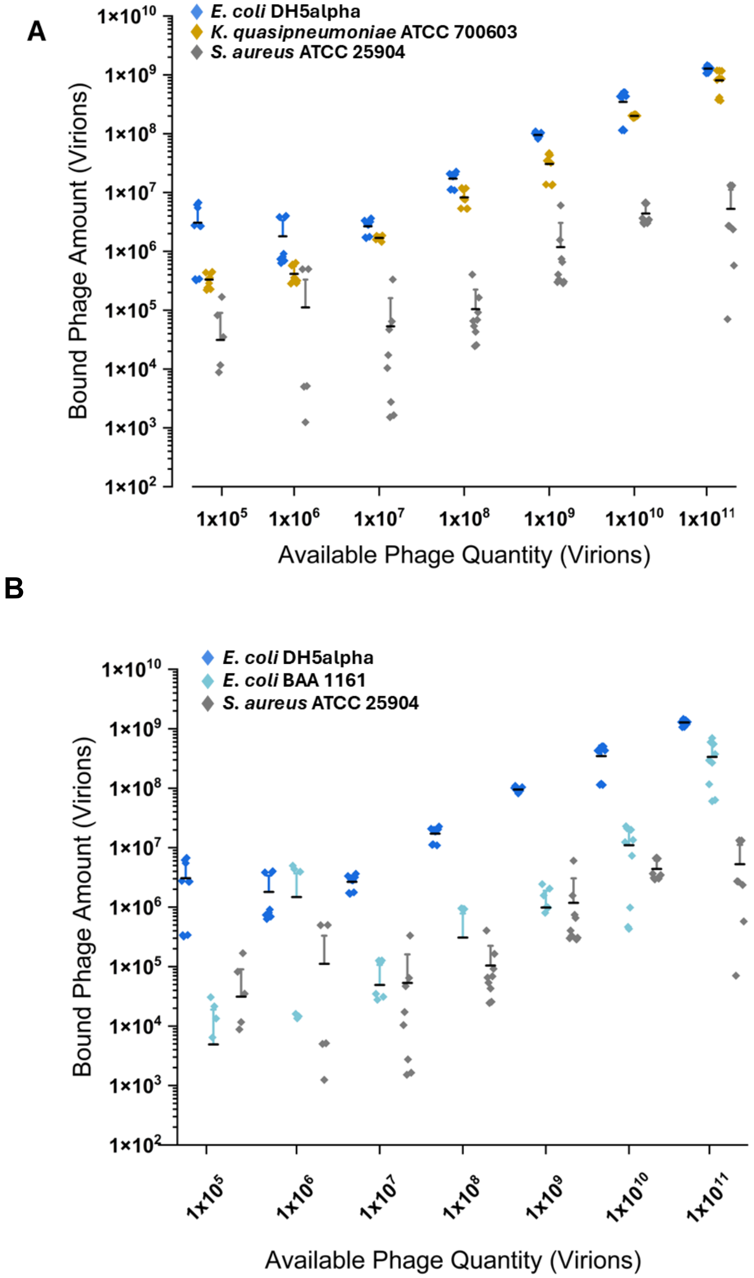

Figure S8. Binding curve showing the amount of M13$^{\alpha LPS}$ bound to cells determined by qPCR, given an increasing incubation amount of phages with a constant amount of bacterial cells (~10^8 colony forming units (cfu)). (A) *E. coli* DH5α (blue), *K. pneumoniae* strain ATCC 700603 (yellow), and gram-positive strain *S. aureus* ATCC 25904 (gray). At 10^7 virions/mL and above, the amount of phage bound to either gram-negative organism significantly exceeds that bound to *S. aureus* by > 1 order of magnitude. (B) M13$^{\alpha LPS}$ phage binding curve for *E. coli* DH5α, *E. coli* ATCC BAA 1161 and negative control strain *S. aureus* ATCC 25904. Mean value (solid black dash) was calculated from three experimental replicates, each including three technical replicates. Error bars (whisker in + direction) representing 1 standard deviation were calculated from experimental triplicates. See Table S4 for data.

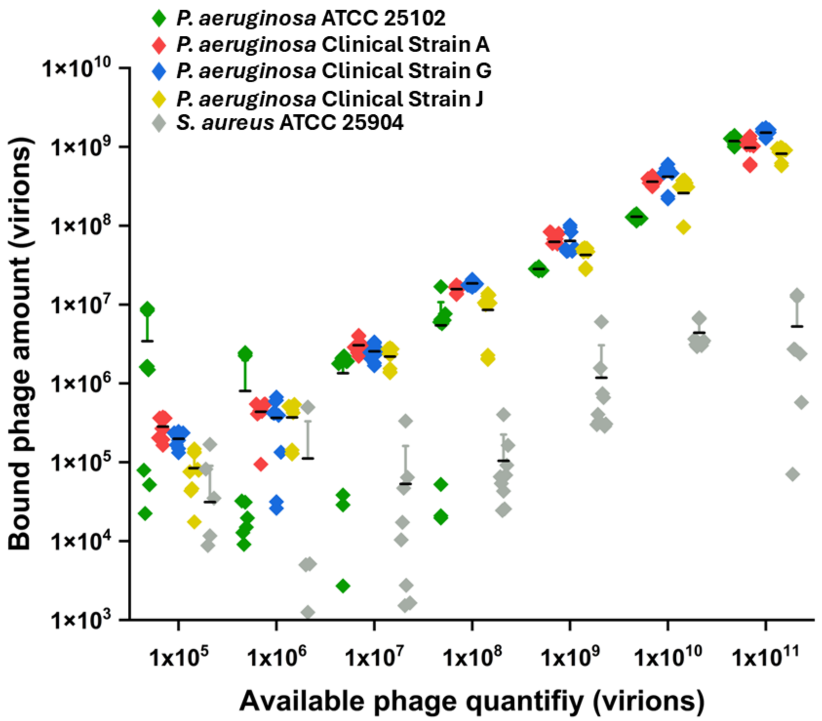

Figure S9. M13$^{\alpha LPS}$ phage binding curve for *P. aeruginosa* strain ATCC 25102, clinical strain A, G and J, as well as negative control strain *S. aureus* ATCC 25904. Same method as shown in Figure S8. Error bars (whisker in + direction) representing 1 standard deviation were calculated from experimental triplicates. See Table S4 for data.

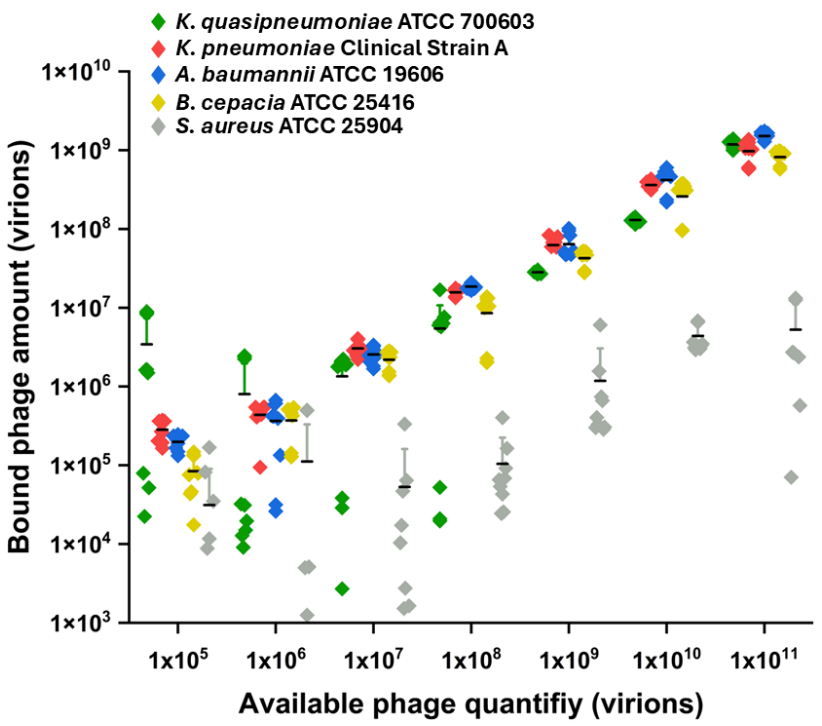

Figure S10. M13$^{\alpha LPS}$ binding curve for *K. pneumoniae* strain ATCC 700604 and clinical strain A, *A. baumannii* strain ATCC 19606, *B. cepacia* strain ATCC 25904 and negative control strain *S. aureus* ATCC 25904. Same method as shown in Figure S8. Error bars (whisker in + direction) representing 1 standard deviation were calculated from experimental triplicates in plus direction. See Table S4 for data.

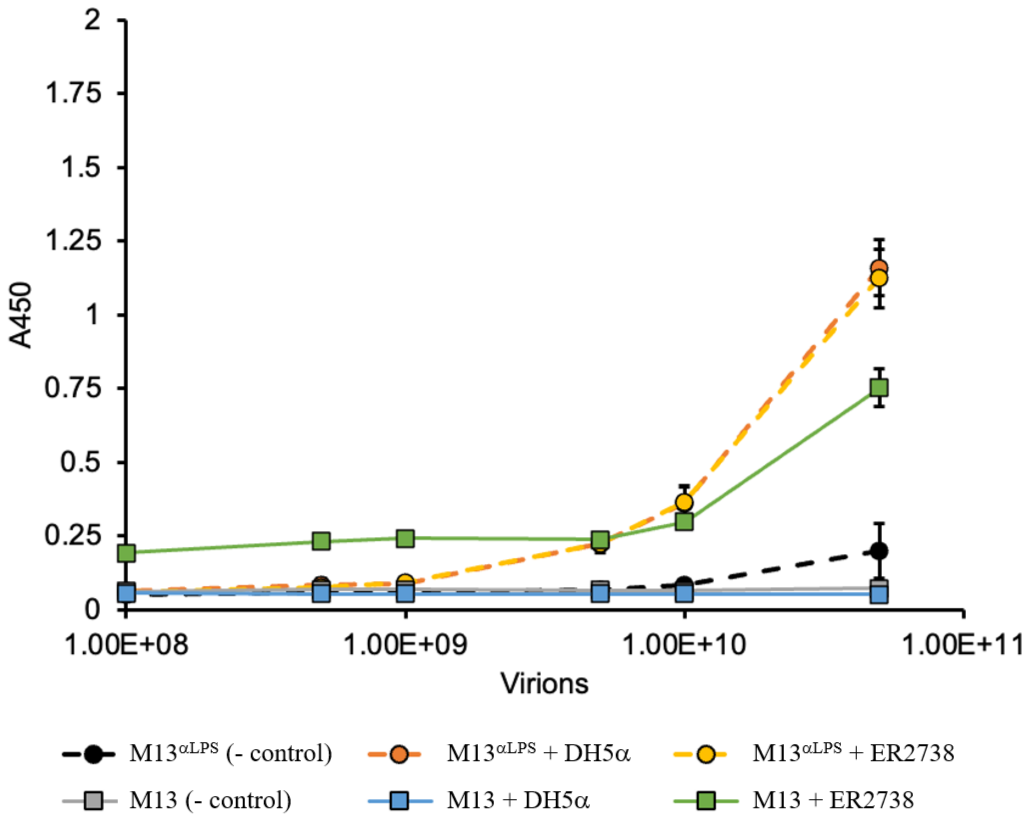

Figure S11. Cell-based ELISA for binding of phage to *E. coli*. *E. coli* cells were adsorbed to plates and incubated with M13 or M13$^{\alpha LPS}$ at varying amounts, as shown on the x-axis. Negative control (- control) wells did not contain cells but were blocked with BSA solution in the same process (see Methods). Phages were detected by an anti-g8p antibody conjugated to HRP. Substrate conversion was measured by absorbance at 450 nm (y-axis). M13 showed signal when mixed with ER2738 but not DH5α. M13$^{\alpha LPS}$ showed signal when mixed with either strain.

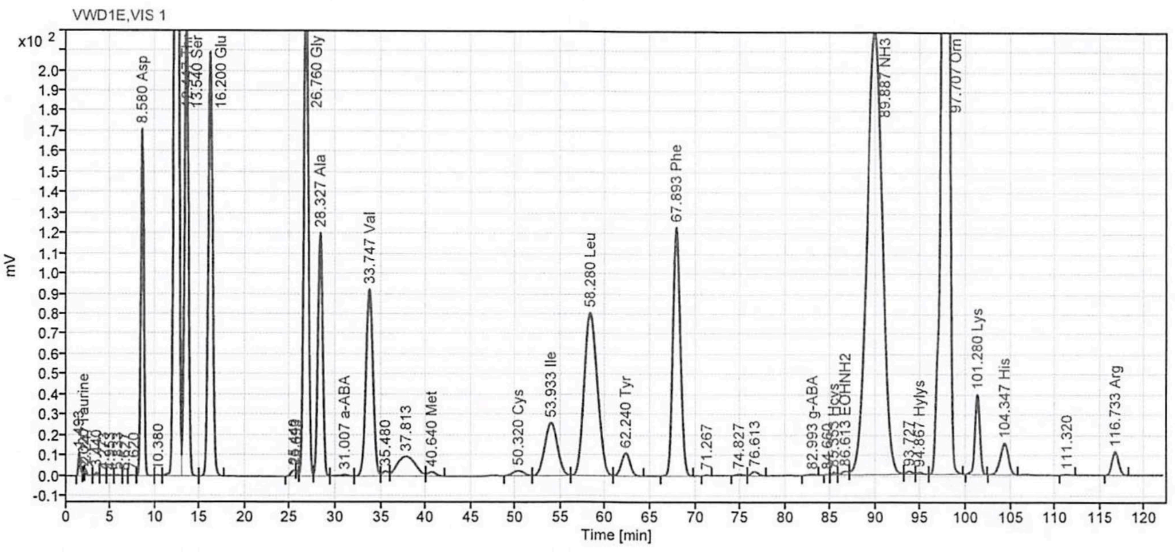

Figure S12. Chromatogram of amino acid composition analysis for PMB-M13$^{\alpha LPS}$.

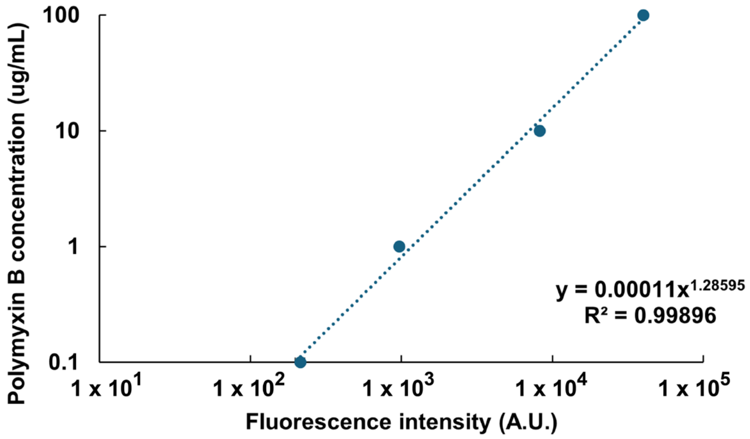

Figure S13. Standard curve of Fluoraldehyde™ o-Phthaldialdehyde Reagent Solution (OPA) for quantifying PMB in 10-fold serial dilutions.

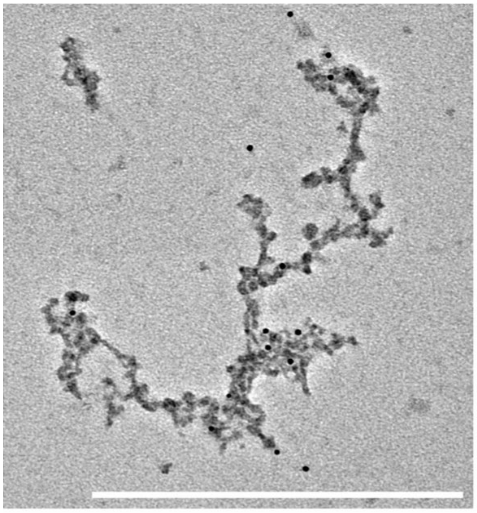

Figure S14. TEM image of PMB-M13$^{\alpha LPS}$. The sample was incubated with anti-PMB mouse monoclonal antibodies and subsequently using gold nanoparticles (black dots) coated with Donkey-anti-Mouse secondary antibodies, demonstrating successful conjugation of PMB to the phage. Compared with M13$^{\alpha LPS}$ phage lacking PMB (Figure 1A), PMB-M13$^{\alpha LPS}$ virions show a tendency toward self-association, likely due to reduced surface charge. Scale bar = 500 nm.

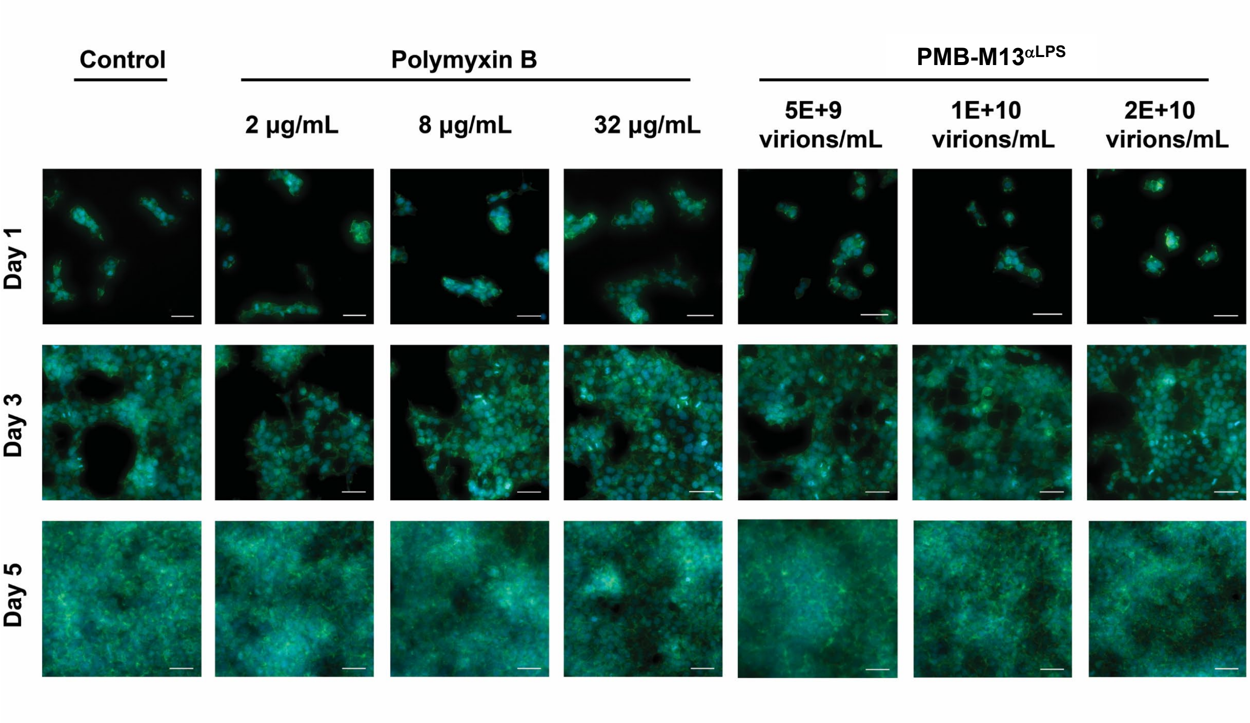

Figure S15. Phalloidin/DAPI staining of HEK 293 cells to examine the cell nucleus, cytoskeleton and cell spreading morphology in the presence of PMB-M13$^{\alpha LPS}$. Cells were grown in 24 well plates and supplemented with media containing different concentrations of PMB and PMB-M13$^{\alpha LPS}$ as indicated in the figure. Cells were stained fluorescently and visualized using a AxioObserver Z1 inverted microscope (Zeiss). Scale bar = 100 µm.

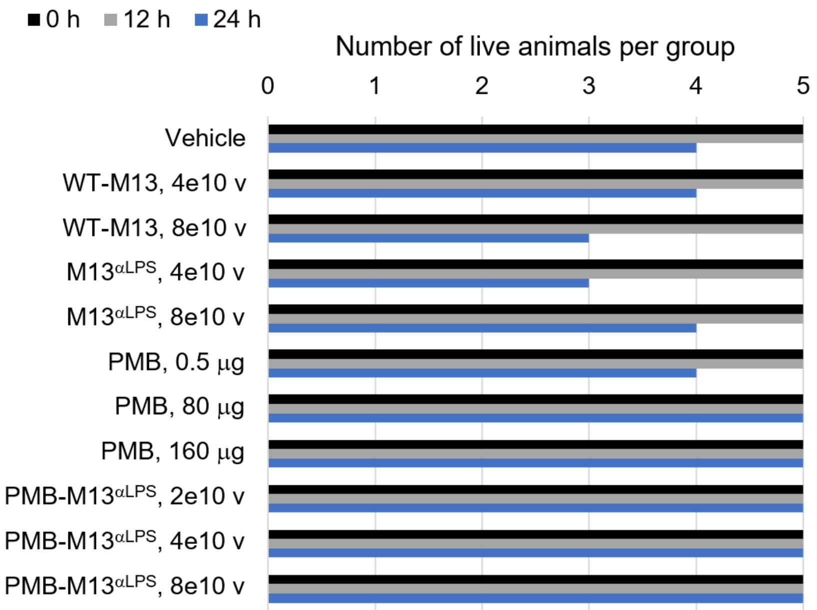

Figure S16. Survival of animals at 24 h in the pneumonia model with different treatments. Each treatment group included 5 animals on Day 0 (black). No animals had reached humane endpoints at 12 h (gray). Some animals had died when checked at 24 h (blue). Surviving animals were sacrificed at 25 h. Also see Figure 4.

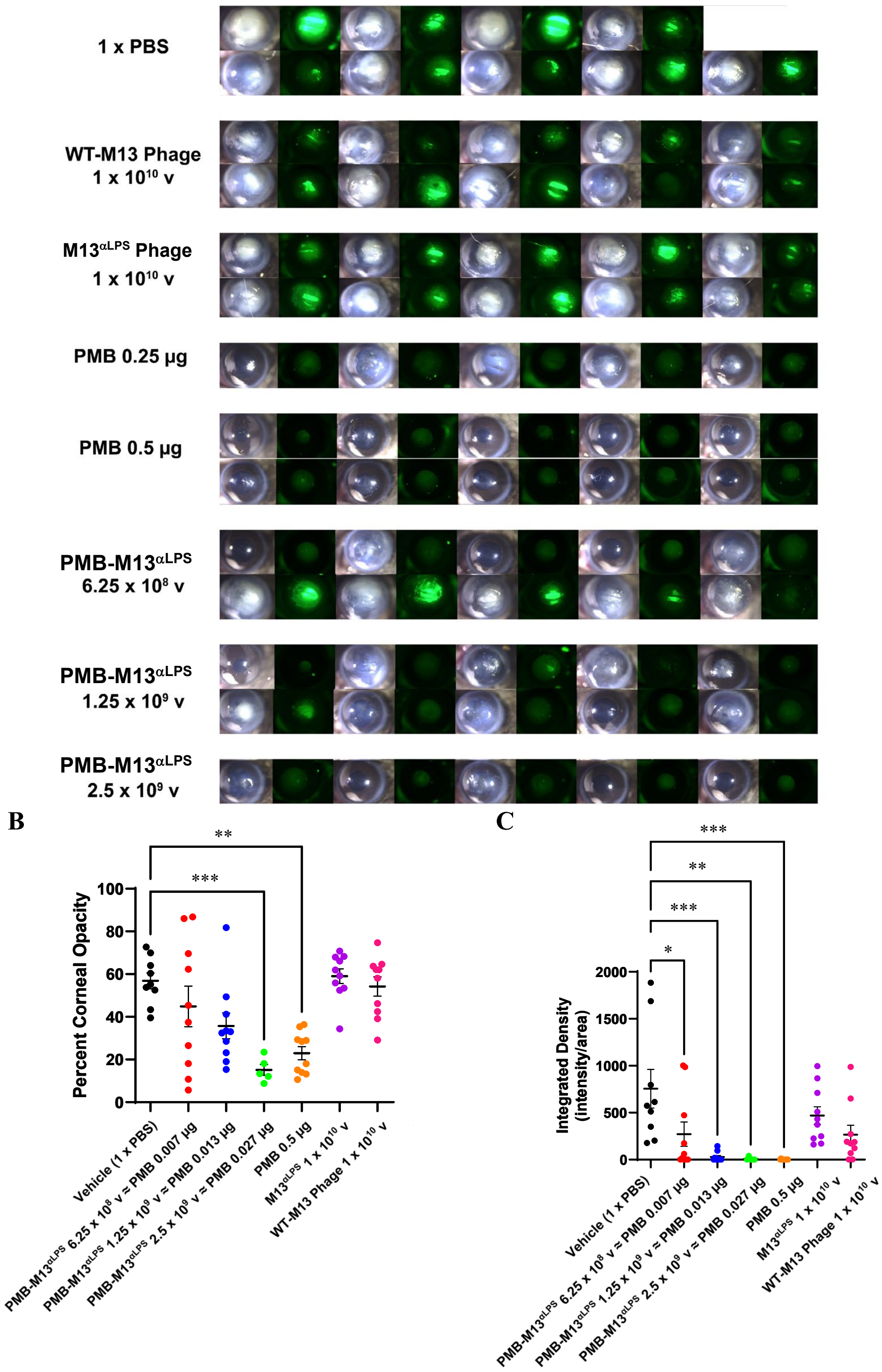

Figure S17. Efficacy of PMB-M13$^{\alpha LPS}$ in a mouse model of *P. aeruginosa* corneal infection. Corneas of C57BL/6 mice were abraded and inoculated with 5 × 10^{4} CFU GFP expressing *P. aeruginosa* (PAO1-GFP). M13$^{\alpha LPS}$, WT-M13 phage, PMB sulfate, or PMB-M13$^{\alpha LPS}$ was administered topically in the first 24 h. Animals

were euthanized 48 h post-infection. **(A)** Brightfield and fluorescent images of infected eyes in each group. **(B)** Corneal opacification and **(C)** GFP fluorescence intensity were quantified by image analysis. Statistical significance of comparisons to the PBS control were determined by one-way ANOVA and Tukey's post hoc test. * means $p<0.05$; ** means $p < 0.01$; *** means $p < 0.0001$. v = virions. 'Vehicle' is treatment with PBS alone. Mean value (solid black dash) and error bars (whiskers) representing 1 standard deviation were calculated from experimental replicates.

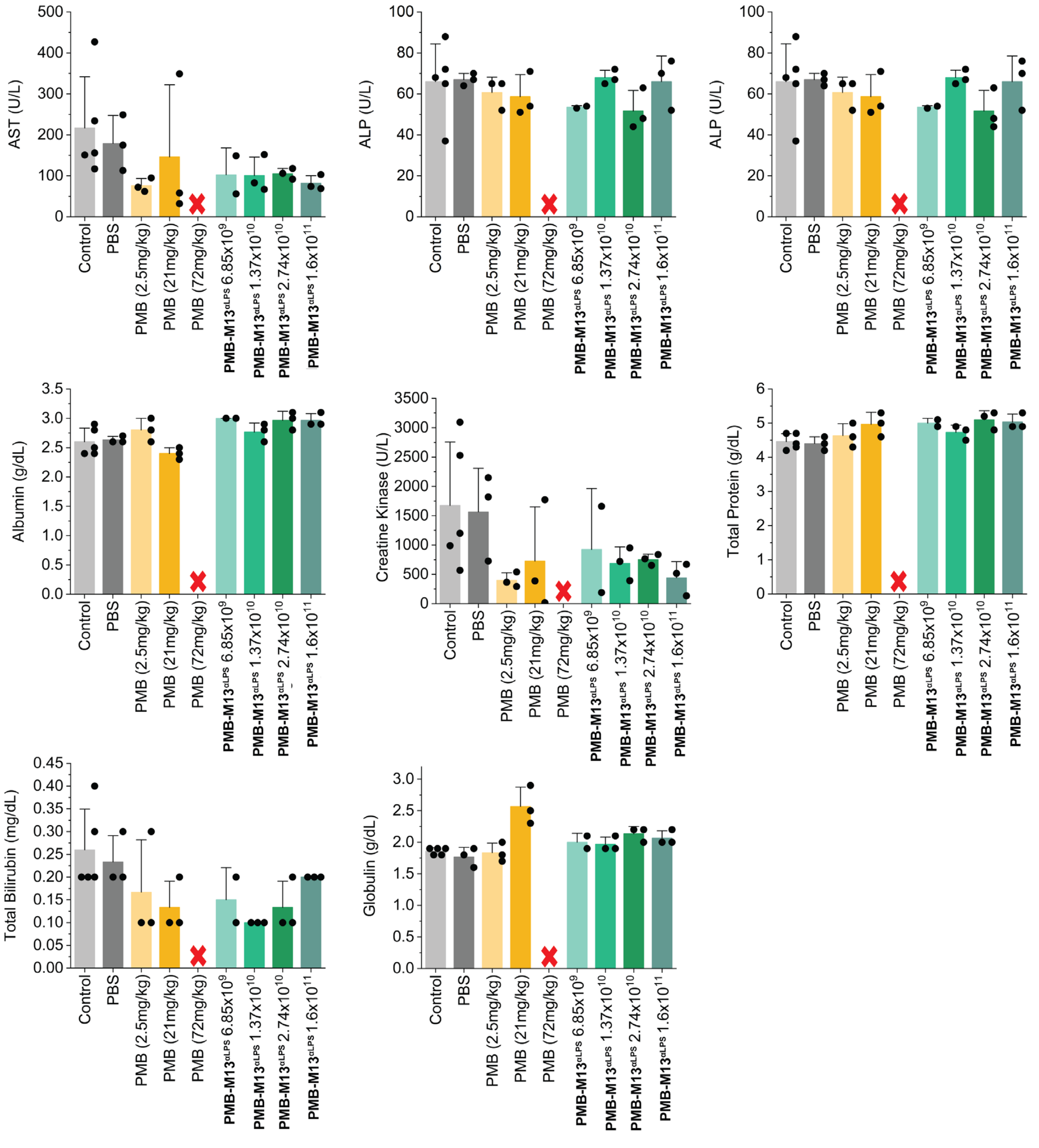

Figure S18. Liver and kidney function blood biomarkers at the end point after 7-day toxicity studies of PMB-M13$^{\alpha LPS}$. Animals in treated groups were injected with 100 µL of test materials (PMB-M13$^{\alpha LPS}$, PMB, or 1x PBS) through IV tail vein injection daily for 7 consecutive days. Biomarker levels did not indicate liver or kidney injury from PMB-M13$^{\alpha LPS}$. However, all animals receiving 72 mg/kg body weight of PMB (N=3) died after the first dose on day 1 (indicated by red 'x' in figure). N=3 per group. Error bars (whiskers in plus direction) representing 1 standard deviation was calculated from experimental replicates.

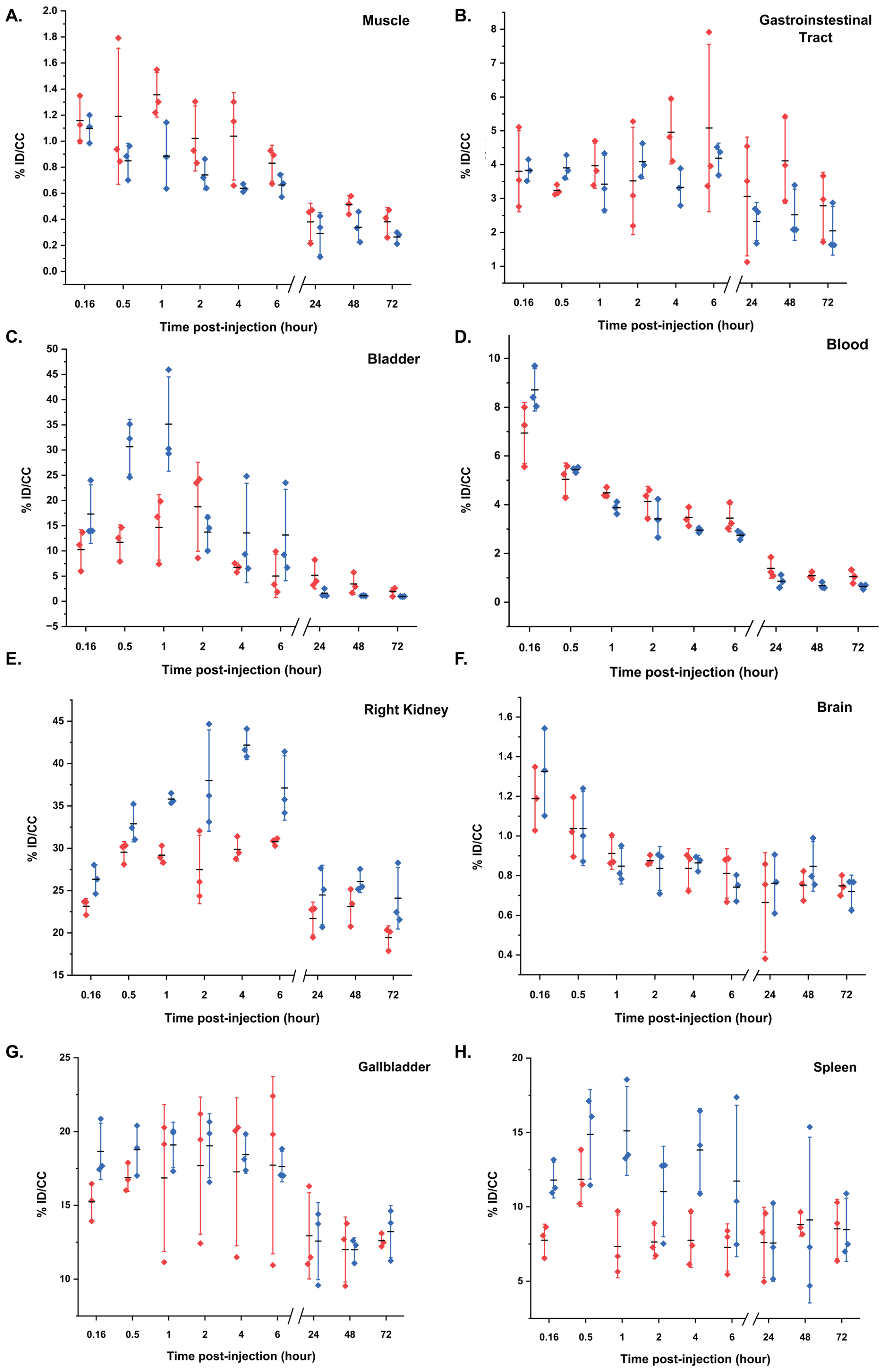

A.
Muscle
% ID/CC
Time post-injection (hour)
B.
Gastroinstestinal Tract
C.
Bladder
D.
Blood
E.
Right Kidney
F.
Brain
G.
Gallbladder
H.
Spleen
0.16
0.5
1
2
4
6
24
48
72

Figure S19. Time course of biodistribution of ^{89}Zr-labeled PMB-M13$^{\alpha LPS}$ (100 μCi) in different organs and tissues: (A) muscle, (B) gastrointestinal tract, (C) bladder, (D) blood, (E) right kidney (F) brain, (G) gallbladder, and (H) spleen over 72 hours. Mice were injected with PMB-M13$^{\alpha LPS}$ alone (blue) or were pre-injected with 1x10^{7} cfu *E. coli* ATCC BAA 1161 (red). Error bars (whiskers) show standard deviation (N=3). Data were quantified from μPET images. Signals are measured as percentages of the injected dose (ID) per volume (cc). Mean value (solid black dash) was calculated from three experimental replicates.

Supporting Tables

Primer Name	5' - 3' Sequence
S239T Forward	GCTGGCGGCGGCACCGGTGGTGGTTCTGGT
S239T Reverse	CGTCTGACCTGCCTCAACCTCCTGTCAAT
pADL-sequencing Forward	AGTGAGCGGTACCCGATAAAAG
pADL-sequencing Reverse	ACCAGCGCCAAAGACATAAG
UpstreamInsert	GAAGTGCCATTCCGCCTGACCTGCGGCCGCTGGATTGTTATTACTCGCGGCCCAGCCGGCCATGGCT
DownstreamInsert	GGCGGCGGCACCGGTAGGCTAGGTGGAGGCTCAGTG
qPCR-upstream	TTGGTGACGTTTCCGGCCTTGC
qPCR-downstream	TCACCGTCACCGACTTGAGCCA
OrfiF1longForward	GGTCGAGGTGCCGTAAAGCACTAAATCGGAACCCTAAAGGG
OriF1longReverse	CCAAAAAACTTGATTAGGGTGATGGTTCACGTA

Table S1. Primer sequences used in this work.

OD(600)	CFU/mL
1.018	3.64E+07
1.075	2.28E+07
1.073	3.26E+07
0.484	1.24E+07
0.46	1.38E+07
0.488	1.32E+07
0.098	3.60E+06
0.098	3.70E+06
0.097	2.76E+06

Table S2. OD at 600 nm vs. colony counts per mL of culture, for *E. coli* BAA 1161.

Species	HLL construct	LLH construct	Bacteria Alone Control
C. sakazakii ATCC 25944	1.25E+07	8.66E+05	1.27E+04
B. cepacia ATCC 25416	2.58E+07	1.60E+06	8.72E+03
A. baumannii ATCC 19606	4.37E+06	2.81E+05	8.58E+03
K. pneumoniae clinical strain A	5.82E+06	1.29E+06	1.16E+04
K. quasipneumoniae ATCC 700603	7.01E+06	3.04E+05	6.81E+03
P. aeruginosa clinical strain J	1.99E+08	5.29E+07	1.27E+04
P. aeruginosa clinical strain G	2.24E+05	7.91E+05	1.24E+04
P. aeruginosa clinical strain F	9.14E+06	3.86E+05	3.55E+04
P. aeruginosa clinical strain E	3.41E+06	2.85E+04	9.29E+03
P. aeruginosa clinical strain C	7.16E+06	2.50E+06	1.18E+04
P. aeruginosa clinical strain B	5.32E+06	6.96E+05	1.77E+04
P. aeruginosa ATCC 25102	8.02E+07	1.06E+07	2.55E+04
E. coli DH5α	2.45E+07	2.80E+04	3.53E+03
MilliQ water	--	--	1.24E+04

Table S3. Amount of HLL or LLH phage bound, quantified as copies by qPCR. Amount of phage incubated = 10^{10} virions. Also see Figure S6.

M13$^{\alpha LPS}$ virions			Bacterial strain	*A. baumannii* ATCC 19606					
1.00E+11	7.09E+8	7.48E+8	7.29E+8	3.06E+8	3.14E+8	3.04E+8	8.08E+8	7.90E+8	8.73E+8
1.00E+10	2.36E+8	2.45E+8	2.32E+8	9.67E+7	9.91E+7	9.96E+7	1.83E+8	1.89E+8	1.82E+8
1.00E+9	4.44E+7	4.47E+7	4.29E+7	3.38E+7	3.44E+7	3.75E+7	5.07E+7	4.92E+7	5.01E+7
1.00E+8	8.29E+6	7.91E+6	8.63E+6	6.18E+6	5.78E+6	5.63E+6	7.90E+6	8.80E+6	8.59E+6
1.00E+7	1.28E+6	1.42E+6	1.26E+6	1.70E+6	3.82E+5	4.77E+5	2.58E+6	2.70E+6	2.71E+6
1.00E+6	6.81E+5	5.96E+5	5.62E+5	9.79E+5	9.69E+5	1.71E+6	9.03E+5	9.52E+5	9.74E+5
1.00E+5	2.26E+5	2.80E+5	2.85E+5	3.01E+6	9.12E+6	3.28E+6	3.37E+6	2.79E+6	2.99E+6
Negative Control	3.83E+5	3.85E+5	4.12E+5	4.10E+6	1.03E+6	9.70E+5	3.62E+6	7.22E+5	7.21E+5
M13$^{\alpha LPS}$ virions			Bacterial strain	*B. cepacia* ATCC 25416					
1.00E+11	1.62E+9	1.34E+9	1.43E+9	1.42E+9	1.32E+9	1.39E+9	1.53E+9	1.59E+9	1.74E+9
1.00E+10	3.86E+8	3.86E+8	3.59E+8	5.64E+8	5.30E+8	5.28E+8	3.86E+8	3.63E+8	3.94E+8
1.00E+9	6.27E+7	6.13E+7	6.10E+7	6.19E+7	6.50E+7	6.42E+7	8.50E+7	8.14E+7	8.31E+7
1.00E+8	1.51E+7	1.42E+7	1.45E+7	1.67E+7	1.44E+7	1.54E+7	1.84E+7	1.67E+7	1.86E+7
1.00E+7	2.75E+6	2.73E+6	2.66E+6	3.22E+6	3.07E+6	3.15E+6	2.51E+6	2.85E+6	2.81E+6
1.00E+6	1.20E+6	1.21E+6	9.37E+5	1.25E+6	1.32E+6	1.28E+6	5.74E+5	6.07E+5	6.24E+5
1.00E+5	3.02E+5	3.25E+5	3.17E+5	4.65E+5	4.66E+5	5.01E+5	3.73E+5	3.72E+5	3.97E+5
Negative Control	4.96E+5	5.03E+5	5.13E+5	3.88E+5	4.23E+5	4.67E+5	4.49E+5	3.80E+5	3.90E+5
M13$^{\alpha LPS}$ virions			Bacterial strain	*E. coli* DH5α					
1.00E+11	1.40E+9	1.38E+9	1.46E+9	1.30E+9	1.30E+9	1.30E+9	1.07E+9	1.06E+9	1.19E+9
1.00E+10	1.19E+8	1.15E+8	1.14E+8	5.06E+8	4.85E+8	5.05E+8	4.22E+8	4.31E+8	4.30E+8
1.00E+9	8.18E+7	8.96E+7	8.82E+7	9.27E+7	9.04E+7	1.09E+8	9.98E+7	1.01E+8	1.04E+8
1.00E+8	1.87E+7	1.99E+7	1.90E+7	1.14E+7	1.10E+7	1.12E+7	2.04E+7	2.08E+7	2.27E+7
1.00E+7	2.84E+6	2.94E+6	2.83E+6	1.68E+6	1.78E+6	1.73E+6	3.18E+6	3.35E+6	3.65E+6
1.00E+6	8.15E+5	7.41E+5	9.15E+5	6.39E+5	7.00E+5	7.47E+5	3.87E+6	3.84E+6	4.03E+6
1.00E+5	5.51E+6	6.20E+6	6.74E+6	3.27E+5	3.48E+5	3.45E+5	2.68E+6	2.77E+6	2.69E+6
Negative Control	3.16E+5	3.25E+5	3.44E+5	3.07E+4	4.22E+4	3.78E+4	2.48E+6	2.50E+6	2.60E+6
M13$^{\alpha LPS}$ virions			Bacterial strain	*E. coli* ATCC BAA 1161					
1.00E+11	5.54E+8	5.96E+8	6.96E+8	6.06E+7	1.17E+8	6.33E+7	2.69E+8	2.95E+8	3.75E+8
Negative Control	1.14E+6	1.24E+6	1.18E+6	2.11E+2	3.43E+1	7.20E+1	1.28E+2	1.19E+2	8.05E+1
M13$^{\alpha LPS}$ virions			Bacterial strain	*K. quasipneumoniae* ATCC 700603					
1.00E+11	1.18E+9	1.19E+9	1.17E+9	8.82E+8	8.19E+8	8.56E+8	3.63E+8	3.78E+8	4.13E+8
1.00E+10	2.14E+8	2.10E+8	2.10E+8	1.87E+8	1.85E+8	1.93E+8	2.03E+8	2.01E+8	2.02E+8
1.00E+9	4.68E+7	4.46E+7	4.33E+7	3.36E+7	3.18E+7	3.49E+7	1.32E+7	1.37E+7	1.37E+7
1.00E+8	7.79E+6	7.88E+6	7.58E+6	1.14E+7	1.19E+7	1.19E+7	5.31E+6	5.35E+6	5.33E+6
1.00E+7	1.74E+6	1.71E+6	1.72E+6	1.70E+6	1.68E+6	1.85E+6	1.46E+6	1.60E+6	1.87E+6
1.00E+6	3.63E+5	3.22E+5	3.60E+5	6.08E+5	6.45E+5	5.80E+5	2.96E+5	2.91E+5	3.24E+5
1.00E+5	2.95E+5	3.45E+5	3.63E+5	2.48E+5	2.35E+5	2.28E+5	4.36E+5	4.43E+5	4.54E+5
Negative Control	4.81E+5	4.73E+5	4.98E+5	1.31E+5	1.37E+5	1.31E+5	1.34E+5	1.48E+5	1.42E+5
M13$^{\alpha LPS}$ virions			Bacterial strain	*K. pneumoniae* clinical strain A					
1.00E+11	3.64E+8	3.63E+8	3.47E+8	2.26E+8	2.31E+8	2.54E+8	1.24E+8	1.24E+8	1.33E+8
1.00E+10	1.69E+8	1.64E+8	1.70E+8	1.34E+8	1.34E+8	1.38E+8	2.74E+7	2.82E+7	2.88E+7
1.00E+9	3.53E+7	3.57E+7	3.60E+7	1.56E+7	1.48E+7	1.57E+7	8.55E+6	8.42E+6	8.54E+6
1.00E+8	5.73E+6	5.81E+6	5.71E+6	4.46E+6	4.44E+6	4.62E+6	1.42E+6	2.00E+6	1.41E+6
1.00E+7	6.33E+5	6.69E+5	7.47E+5	1.12E+6	1.12E+6	1.07E+6	9.11E+4	8.88E+4	1.19E+5
1.00E+6	3.45E+5	3.27E+5	3.26E+5	1.39E+5	1.39E+5	1.36E+5	2.84E+5	2.98E+5	2.86E+5
1.00E+5	2.26E+5	2.33E+5	2.34E+5	5.48E+4	5.41E+4	5.28E+4	8.35E+4	1.06E+5	1.01E+5
Negative Control	1.20E+5	1.11E+5	1.30E+5	7.97E+4	5.23E+4	8.74E+3	7.45E+4	8.01E+4	2.60E+4
M13$^{\alpha LPS}$ virions			Bacterial strain	*P. aeruginosa* ATCC 25102					
1.00E+11	1.39E+9	1.30E+9	1.24E+9	1.27E+9	1.23E+9	1.07E+9	1.01E+9	1.10E+9	9.94E+8
1.00E+10	1.42E+8	1.36E+8	1.30E+8	1.36E+8	1.31E+8	1.30E+8	1.15E+8	1.28E+8	1.24E+8
1.00E+9	2.87E+7	2.73E+7	2.84E+7	3.02E+7	3.01E+7	2.84E+7	2.70E+7	2.67E+7	2.68E+7
1.00E+8	6.45E+6	6.00E+6	5.72E+6	2.81E+4	5.95E+4	2.68E+4	6.33E+6	1.69E+7	7.65E+6
1.00E+7	2.17E+6	2.09E+6	2.23E+6	9.81E+3	3.60E+4	4.55E+4	1.89E+6	1.79E+6	1.91E+6
1.00E+6	3.83E+4	1.62E+4	2.21E+4	1.99E+4	2.67E+4	3.94E+4	2.38E+6	2.47E+6	2.25E+6
1.00E+5	1.66E+6	1.62E+6	1.49E+6	2.96E+4	5.90E+4	8.66E+4	8.97E+6	8.67E+6	8.31E+6
Negative Control	6.93E+6	1.08E+7	1.68E+7	9.73E+4	8.17E+4	1.23E+5	3.77E+4	3.30E+4	3.07E+4

M13$^{\alpha LPS}$ virions			Bacterial strain		*P. aeruginosa* clinical strain A				
1.00E+11	1.05E+9	1.06E+9	1.02E+9	5.96E+8	5.77E+8	6.09E+8	1.25E+9	1.22E+9	1.37E+9
1.00E+10	3.44E+8	3.44E+8	3.47E+8	3.40E+8	3.13E+8	3.48E+8	3.82E+8	4.32E+8	3.97E+8
1.00E+9	6.37E+7	6.75E+7	6.79E+7	2.82E+3	5.94E+7	5.86E+7	8.16E+7	7.97E+7	8.35E+7
1.00E+8	1.60E+7	1.65E+7	1.59E+7	1.39E+7	1.34E+7	1.37E+7	1.65E+7	1.78E+7	1.74E+7
1.00E+7	2.36E+6	2.46E+6	2.41E+6	4.07E+6	3.96E+6	4.08E+6	2.21E+6	2.88E+6	3.01E+6
1.00E+6	4.74E+5	4.58E+5	4.48E+5	4.31E+5	1.02E+5	4.18E+5	5.52E+5	5.54E+5	5.53E+5
1.00E+5	2.68E+5	2.76E+5	3.62E+5	3.81E+5	3.69E+5	3.71E+5	1.71E+5	2.11E+5	2.01E+5
Negative Control	2.83E+5	3.02E+5	3.41E+5	3.37E+5	3.26E+5	2.96E+5	1.62E+5	1.77E+5	2.28E+5
M13$^{\alpha LPS}$ virions			Bacterial strain		*P. aeruginosa* clinical strain B				
1.00E+11	9.43E+8	9.05E+8	9.21E+8	1.27E+9	1.24E+9	1.18E+9			
Negative Control	1.50E+2	2.57E+2	2.13E+2	4.38E+3	4.67E+3	6.72E+3	2.93E+3	3.46E+3	3.89E+3
M13$^{\alpha LPS}$ virions			Bacterial strain		*P. aeruginosa* clinical strain C				
1.00E+11	8.49E+8	8.78E+8	9.27E+8	1.04E+9	1.17E+9	1.04E+9	1.12E+9	1.13E+9	1.17E+9
Negative Control	1.59E+3	1.18E+3	1.70E+3	4.86E+2	3.80E+2	3.03E+2	4.61E+3	3.64E+3	8.01E+3
M13$^{\alpha LPS}$ virions			Bacterial strain		*P. aeruginosa* clinical strain E				
1.00E+11	1.03E+9	1.09E+9	1.05E+9	5.62E+8	5.81E+8	7.58E+8	1.44E+9	1.42E+9	1.42E+9
Negative Control	9.96E+2	1.59E+3	1.72E+3	1.03E+3	1.10E+3	1.08E+3	3.41E+3	3.91E+3	8.66E+3
M13$^{\alpha LPS}$ virions			Bacterial strain		*P. aeruginosa* clinical strain F				
1.00E+11	9.72E+8	9.98E+8	9.48E+8	1.15E+9	1.16E+9	1.13E+9	8.48E+8	9.16E+8	8.33E+8
1.00E+5	5.13E+2	7.40E+2	5.33E+2	1.43E+3	9.78E+2	1.14E+3	2.41E+3	1.91E+3	2.83E+3
Negative Control									
M13$^{\alpha LPS}$ virions			Bacterial strain		*P. aeruginosa* clinical strain G				
1.00E+11	1.09E+9	8.56E+8	1.07E+9	9.87E+8	9.40E+8	1.04E+9	7.30E+8	7.46E+8	7.40E+8
Negative Control	1.53E+3	2.68E+3	2.39E+3	2.54E+3	2.65E+3	2.90E+3	3.79E+3	5.13E+3	5.79E+3
M13$^{\alpha LPS}$ virions			Bacterial strain		*P. aeruginosa* clinical strain J				
1.00E+11	1.05E+9	1.06E+9	1.02E+9	5.96E+8	5.77E+8	6.09E+8	1.25E+9	1.22E+9	1.37E+9
1.00E+10	3.44E+8	3.44E+8	3.47E+8	3.40E+8	3.13E+8	3.48E+8	3.82E+8	4.32E+8	3.97E+8
1.00E+9	6.37E+7	6.75E+7	6.79E+7	2.82E+3	5.94E+7	5.86E+7	8.16E+7	7.97E+7	8.35E+7
1.00E+8	1.60E+7	1.65E+7	1.59E+7	1.39E+7	1.34E+7	1.37E+7	1.65E+7	1.78E+7	1.74E+7
1.00E+7	2.36E+6	2.46E+6	2.41E+6	4.07E+6	3.96E+6	4.08E+6	2.21E+6	2.88E+6	3.01E+6
1.00E+6	4.74E+5	4.58E+5	4.48E+5	4.31E+5	1.02E+5	4.18E+5	5.52E+5	5.54E+5	5.53E+5
1.00E+5	2.68E+5	2.76E+5	3.62E+5	3.81E+5	3.69E+5	3.71E+5	1.71E+5	2.11E+5	2.01E+5
Negative Control	2.83E+5	3.02E+5	3.41E+5	3.37E+5	3.26E+5	2.96E+5	1.62E+5	1.77E+5	2.28E+5
M13$^{\alpha LPS}$ virions			Bacterial strain		*P. aeruginosa* PAKpmrB6				
1.00E+11	7.79E+7	6.84E+7	7.59E+7	1.13E+3	1.41E+3	1.23E+3	3.96E+8	4.06E+8	4.93E+8
Negative Control	6.74E+3	6.19E+3	7.13E+3	1.99E+4	2.58E+4	2.15E+4	2.86E+0	1.96E-1	6.58E-1
M13$^{\alpha LPS}$ virions			Bacterial strain		*S. aureus* ATCC 25904				
1.00E+11	1.25E+7	1.32E+7	1.32E+7	2.69E+6	2.38E+6	2.72E+6	5.82E+5	1.53E+2	7.76E+4
1.00E+10	6.80E+6	6.64E+6	6.57E+6	2.93E+6	3.03E+6	3.11E+6	3.32E+6	3.67E+6	3.48E+6
1.00E+9	6.06E+6	1.57E+6	7.49E+5	3.38E+5	6.67E+5	4.10E+5	2.93E+5	3.07E+5	3.13E+5
1.00E+8	5.01E+4	3.16E+4	3.27E+4	6.09E+4	7.56E+4	7.25E+4	9.90E+4	4.10E+5	1.71E+5
1.00E+7	9.86E+3	6.71E+3	8.62E+3	7.15E+4	5.41E+4	3.41E+5	2.44E+4	8.74E+3	1.75E+4
1.00E+6	6.20E+3	6.24E+3	6.17E+3	8.35E+3	1.21E+4	1.22E+4	5.30E+3	5.04E+5	5.07E+5
1.00E+5	3.61E+2	1.88E+4	1.59E+4	1.75E+5	8.90E+4	4.21E+4	1.14E+3	1.29E+3	1.73E+3
Negative Control	1.69E+2	1.90E+2	1.42E+2	5.26E+3	4.98E+3	1.77E+4	8.71E+3	9.46E+3	1.73E+4

Table S4. The amount of M13$^{\alpha LPS}$ bound to the cells and negative controls of each cell strain, quantified using qPCR by mixing 1 mL of cells (OD_{600}=1) with 100 μL phage (10^5 to 10^{11} virions) as indicated.

Amino acid	# per M13$^{\alpha LPS}$ phage virion (*x*=0)	Mole % on M13$^{\alpha LPS}$ phages (*x*=0)	Measured mole %	Estimated number of PMB molecules per g8p (*x*)
Asx	8450	6.08	5.21	2
N(Asn)	2860	2.06	n.d.	n.d.
D(Asp)	5590	4.02	n.d.	n.d.
T(Thr)	5765	6.02	24.05	9
S(Ser)	1135	8.13	8.77	0
Glx	8385	6.03	8.57	0
Q(Gln)	2855	2.05	n.d.	n.d.
E(Glu)	5530	3.98	n.d.	n.d.
P(Pro)	2865	2.06	5.79	0
G(Gly)	11305	8.13	10.13	0
A(Ala)	27260	19.61	5.29	35
V(Val)	11050	7.95	6.13	4
I(Ile)	11015	7.92	3.13	20
L(Leu)	8370	4.15	9.81	5
W(Trp)	2755	1.98	n.d.	n.d.
F(Phe)	8295	5.97	8.64	2
H(His)	30	0.02	1.15	0
K(Lys)	13675	9.84	1.64	65
R(Arg)	120	0.09	0.75	0
C(Cys)	70	0.05	n.d.	n.d.
Y(Tyr)	5570	4.01	0.95	41

Table S5. Amino acid composition analysis for PMB-M13$^{\alpha LPS}$. For each amino acid analyzed, the number present on an M13$^{\alpha LPS}$ phage virion was calculated assuming 5 copies of each minor capsid protein and 2700 copies of g8p (no PMB). The corresponding mole percentage (no PMB) and the actual measured mole percentage of each amino acid on PMB-M13$^{\alpha LPS}$ are given. Expected mole percentages for whole numbers of PMB (x = 1, 2, 3...) conjugated per g8p were calculated using a Matlab script (Text S2). The value of x giving the closest mole percentage to the observed value for each amino acid is given in the last column. The median value of all 15 estimates of x was 2, which was used as the overall estimated number of PMB molecules per g8p. n.d. = not determined by amino acid analysis.

		**MBC**			
		PMB (µg/mL)	PMB-M13$^{\alpha LPS}$		$\frac{\mathbf{MBC_{PMBSO4}}}{\mathbf{MBC_{PMB\text{-}M13\alpha LPS}}}$
**Species**	**Strain**		(virions/mL)	(µg/mL)	**(n-fold reduction in MBC)**
E. coli	ATCC 25922	2	5.0E+09	0.054	**37**
E. coli	ATCC BAA 1161	2	5.0E+09	0.054	**37**
E. coli	ATCC 700927	2	5.0E+09	0.054	**37**
P. aeruginosa	ATCC 25102	2	1.0E+10	0.107	**19**
P. aeruginosa	Clinical Strain E	2	1.0E+10	0.107	**19**
P. aeruginosa	PAKpmrB6	32	> 8.0E+10	> 0.859	**-**
P. aeruginosa	GFP-PAO1	2	2.5E+09	0.027	**74**
K. quasipneumoniae	ATCC 700603	4	1.0E+10	0.107	**37**
K. pneumoniae	Clinical Strain A	4	1.0E+10	0.107	**37**
K. pneumoniae	Clinical Strain 326	> 256	> 8.0E+10	> 0.859	**-**
A. baumannii	ATCC 19606	2	1.0E+10	0.107	**19**
B. cepacia	ATCC 25416	>.256	> 8.0E+10	> 0.859	-

Table S6. Minimum bactericidal concentration (MBC) of PMB and PMB-M13$^{\alpha LPS}$, determined *in vitro* for several gram-negative organisms.

Replicate number	*1*	*2*	*3*	*4*
PMB concentration (µg/mL)				
**200**	**cleared**	**cleared**	**cleared**	**cleared**
180	countable	cleared	cleared	cleared
160	countable	cleared	cleared	cleared
140	countable	countable	countable	cleared
120	countable	countable	countable	countable
100	countable	countable	countable	countable
Replicate number	*1*	*2*	*3*	*4*
PMB-M13$^{\alpha LPS}$ concentration (virions/mL)				
2.00E+12	cleared	cleared	cleared	cleared
**1.00E+12**	**cleared**	**cleared**	**cleared**	**cleared**
5.00E+11	countable	countable	countable	countable
2.50E+11	full plate	full plate	full plate	full plate
1.25E+11	full plate	full plate	full plate	full plate
6.25E+10	full plate	full plate	full plate	full plate

Table S7. MBC assay for *P. aeruginosa* biofilm, plating results for 300 µL of sample. Cleared = no colonies. Countable = individual colonies observed. Full plate = confluent lawn. The concentration giving no colonies on all 4 replicates was taken as the MBC (bold lines).

PMB-M13$^{\alpha LPS}$ concentration (virions/mL)	Week 1			Week 2			Week 3			Week 4		
4.00E+10	0.042	0.041	0.041	0.042	0.041	0.043	0.043	0.042	0.043	0.042	0.041	0.041
2.00E+10	0.042	0.042	0.042	0.042	0.041	0.042	0.043	0.043	0.042	0.043	0.042	0.041
1.00E+10	0.043	0.042	0.041	0.043	0.041	0.042	0.043	0.041	0.042	0.043	0.042	0.041
5.00E+09	0.043	0.041	0.042	0.043	0.041	0.042	0.046	0.042	0.042	0.043	0.041	0.041
2.50E+09	0.966	0.042	0.869	0.043	0.041	0.041	1.129	0.042	0.042	0.043	0.043	0.042
1.25E+09	0.997	0.936	0.914	1.046	1.015	0.943	1.105	0.959	1.078	0.878	0.845	0.855
Growth Control	0.990	0.977	0.961	1.080	1.042	1.034	1.202	1.154	1.012	0.883	0.917	0.973
Sterility Control	0.043	0.043	0.043	0.045	0.042	0.041	0.043	0.042	0.042	0.043	0.041	0.041
PMB-M13$^{\alpha LPS}$ concentration (virions/mL)	Week 5			Week 6			Week 7			Week 8		
4.00E+10	0.042	0.040	0.041	0.041	0.041	0.041	0.040	0.040	0.041	0.041	0.040	0.041
2.00E+10	0.041	0.042	0.041	0.041	0.043	0.041	0.041	0.040	0.041	0.041	0.040	0.041
1.00E+10	0.045	0.042	0.040	0.042	0.042	0.041	0.041	0.041	0.041	0.041	0.040	0.040
5.00E+09	0.042	0.041	0.040	0.041	0.041	0.042	0.041	0.095	0.041	0.041	0.042	0.041
2.50E+09	0.042	0.041	0.043	0.961	0.875	0.860	0.046	0.880	0.511	0.042	0.042	0.040
1.25E+09	0.582	0.045	0.042	0.958	0.928	0.916	1.003	0.952	0.907	1.059	1.121	1.075
Growth Control	1.032	1.007	1.013	1.039	0.976	0.964	1.033	0.973	0.957	1.158	1.052	1.019
Sterility Control	0.043	0.043	0.042	0.042	0.042	0.041	0.043	0.041	0.041	0.042	0.041	0.040
PMB-M13$^{\alpha LPS}$ concentration (virions/mL)	Week 9			Week 10			Week 11			Week 12		
4.00E+10	0.041	0.041	0.041	0.040	0.040	0.041	0.041	0.041	0.041	0.041	0.041	0.041
2.00E+10	0.042	0.042	0.041	0.040	0.040	0.040	0.041	0.041	0.042	0.041	0.041	0.041
1.00E+10	0.042	0.041	0.041	0.041	0.041	0.040	0.041	0.040	0.041	0.042	0.040	0.041
5.00E+09	0.043	0.041	0.042	0.041	0.040	0.040	0.041	0.040	0.041	0.042	0.040	0.040
2.50E+09	0.043	0.041	0.856	1.005	0.984	0.725	0.042	0.483	0.041	0.042	0.045	0.729
1.25E+09	1.040	0.974	0.942	1.282	1.208	1.217	0.979	0.948	0.900	1.084	1.015	1.013
Growth Control	1.025	1.018	0.949	1.246	1.244	1.236	1.024	1.008	0.963	1.090	1.022	0.987
Sterility Control	0.042	0.042	0.041	0.041	0.041	0.041	0.042	0.042	0.042	0.041	0.041	0.040

Table S8. MIC assays in triplicate over time for a preparation of PMB-M13$^{\alpha LPS}$. OD values are given with the MIC value highlighted in green for each series. The MIC of 2.5 to 5 x 10^9 virions/mL did not change appreciably over time, indicating stability during storage.

PMB Sulfate (SO_4) MIC		PMB Sulfate (SO_4) concentration (μg/mL)											
Bacteria Species	strain	GC	0.5	1	2	4	8	16	32	64	128	258	SC
E. coli	ATCC BAA 1161	0.93	0.83	0.81	0.04	0.04	0.04	0.04	0.04	0.04	0.04	0.04	0.04
		0.90	0.82	0.80	0.04	0.04	0.04	0.04	0.04	0.04	0.04	0.04	0.04
		0.92	0.86	0.87	0.05	0.05	0.04	0.05	0.04	0.05	0.04	0.04	0.04
P. aeruginosa	ATCC 25102	0.95	0.95	0.99	0.04	0.04	0.04	0.04	0.04	0.04	0.04	0.04	0.04
		0.97	0.88	1.04	0.04	0.04	0.04	0.04	0.04	0.04	0.04	0.04	0.04
		1.03	0.90	0.98	0.04	0.04	0.04	0.04	0.04	0.04	0.04	0.04	0.04
A. baumannii	ATCC 19606	0.84	0.80	0.44	0.04	0.04	0.04	0.04	0.04	0.04	0.05	0.05	0.05
		0.84	0.82	0.48	0.05	0.04	0.05	0.05	0.04	0.05	0.05	0.05	0.04
		0.81	0.78	0.53	0.04	0.05	0.05	0.05	0.05	0.05	0.05	0.05	0.04

PMB-M13$^{\alpha LPS}$ MIC		PMB-M13$^{\alpha LPS}$ concentration (μg/mL PMB equivalent)							
Bacteria Species	strain	GC	0.003	0.007	0.013	0.027	0.054	0.107	SC
E. coli	ATCC BAA 1161	0.58	0.62	0.62	0.60	0.32	0.04	0.04	0.04
		0.57	0.56	0.56	0.56	0.04	0.05	0.04	0.04
		0.53	0.55	0.57	0.56	0.04	0.04	0.04	0.04
P. aeruginosa	ATCC 25102	0.63	0.64	0.66	0.69	0.22	0.04	0.04	0.04
		0.65	0.64	0.68	0.69	0.31	0.04	0.04	0.04
		0.64	0.64	0.66	0.67	0.20	0.04	0.04	0.04
A. baumannii	ATCC 19606	0.43	0.42	0.36	0.34	0.21	0.05	0.04	0.04
		0.44	0.42	0.38	0.30	0.04	0.04	0.04	0.04
		0.43	0.41	0.36	0.27	0.05	0.04	0.04	0.04

Table S9. Raw plate reader data for Figure 2A. Triplicate data was collected for the listed species and strains, the OD_{600} value was read on a plate reader as indication of cell growth. GC: growth control, SC: sterility control.

Antibiotic	MIC (µg/mL)	
Amikacin	8	S
Gentamicin	>16	R
Piperacillin/Tazobactam	64/4	I
Aztreonam	8	S
Aztreonam/avibactam	8/4	-
Cefazolin	>32	-
Cefepime	8-16	S/I
Cefoxitin	>32	-
Ceftazidime	16-32	I/R
Ceftazidime/avibactam	2/4	S
Ceftriaxone	>64	-
Cefiderocol	0.5-1	S
Imipenem	16-32	R
Imipenem/chelators	16	-
Imipenem/relebactam	2/4	-
Meropenem	4-16	R
Ciprofloxacin	>8	R
Colistin	2	S
Tigecycline	>8	-
Trimethoprim/Sulfamethoxazole	4/76	-

Table S10. Antibiotic resistances for *P. aeruginosa* AR Bank #0266. MICs and antibiotic susceptibility were determined following the Clinical Laboratory Standards Institute (CLSI) M07-Ed11 microdilution procedure and the CLSI M100 interpretive criteria. S denotes susceptible, R denotes resistant or not susceptible, and I denotes intermediate susceptibility. According to the CDC & FDA AR Isolate Bank annotations made using Resfinder Analysis of the whole genome sequence, strain AR Bank #0266 carries the aph(3')-IIb gene for aminoglycoside resistance, the OXA-395, PDC-5 resistance genes for β-lactam resistance, the fosA gene for fosfomycin resistance, and bcr1, catB7 genes for phenicols/bicyclomycin resistance.

Group	Test Article	Dose Route/ Schedule	Scheduled Sacrifice Time	Animal No.	Lung Weight (g)	CFU/lung	Log (CFU/lung)	δ	Δ
1	Baseline counts	N/A	1 h	1	0.143	6.30E+06	6.80		
				2	0.139	9.40E+05	5.97		
				3	0.127	4.40E+06	6.64		
				4	0.143	1.15E+06	6.06		
				5	0.134	3.20E+06	6.51		
				Mean	0.137	3.20E+06	6.40	--	--
				SEM	0.003	1.01E+06	0.16		
2	Vehicle (1xPBS)	50 µL/mouse IN, QD	25 h	1	0.216	1.24E+07	7.09		
				2[d]	N/A	N/A	N/A		
				3	0.251	3.30E+08	8.52		
				4	0.213	1.27E+08	8.10		
				5	0.224	4.80E+06	6.68		
				Mean	0.226	1.19E+08	7.60	--	1.20
				SEM	0.009	7.58E+07	0.43		
3	PT# 1284598 (NID-442) ($M13^{\alpha LPS}$ phage)	8×10^{10} virions/dose IN, QD	25 h	1	0.206	8.10E+07	7.91		
				2	0.350	1.45E+09	9.16		
				3	0.184	7.70E+06	6.89		
				4	0.233	3.30E+06	6.52		
				5[d]	N/A	N/A	N/A		
				Mean	0.243	3.86E+08	7.62	0.02	1.22
				SEM	0.037	3.55E+08	0.59		
4	PT# 1284598 (NID-442) ($M13^{\alpha LPS}$ phage)	4×10^{10} virions/dose IN, QD	25 h	1	0.210	6.60E+08	8.82		
				2[d]	N/A	N/A	N/A		
				3	0.207	1.16E+08	8.06		
				4	0.193	2.14E+07	7.33		
				5[d]	N/A	N/A	N/A		
				Mean	0.203	2.66E+08	8.07	0.47	1.67
				SEM	0.005	1.99E+08	0.43		
5	PT# 1284599 (NID-443) (WT-M13 phage)	8×10^{10} virions/dose IN, QD	25 h	1	0.291	2.00E+09	9.30		
				2[d]	N/A	N/A	N/A		
				3	0.193	3.60E+06	6.56		
				4[d]	N/A	N/A	N/A		
				5	0.333	5.80E+09	9.76		
				Mean	0.272	2.60E+09	8.54	0.94	2.14
				SEM	0.041	1.70E+09	1.00		
6	PT# 1284599 (NID-443) (WT-M13 phage)	4×10^{10} virions/dose IN, QD	25 h	1[d]	N/A	N/A	N/A		
				2	0.231	4.70E+09	9.67		
				3	0.198	4.10E+06	6.61		
				4	0.255	8.90E+08	8.95		
				5	0.218	3.10E+06	6.49		
				Mean	0.226	1.40E+09	7.93	0.33	1.53
				SEM	0.012	1.12E+09	0.81		

Group	Test Article	Dose Route/ Schedule	Scheduled Sacrifice Time	Animal No.	Lung Weight (g)	CFU/lung	Log (CFU/lung)	δ	Δ
7	PT# 1284600 (NID-444) (PMB)	160 µg/dose IN, QD	25 h	1	0.192	1.70E+02	2.23		
				2	0.186	4.00E+05	5.60		
				3	0.183	3.50E+02	2.54		
				4	0.188	3.10E+03	3.49		
				5	0.192	3.00E+02	2.48		
				Mean	0.188	8.08E+04	3.27*	-4.33*	-3.13[#]
				SEM	0.002	7.98E+04	0.62		

8	PT# 1284600 (NID-444) (PMB)	80 µg/dose IN, QD	25 h	1	0.192	4.50E+05	5.65		
				2	0.180	9.90E+04	5.00		
				3	0.171	6.30E+04	4.80		
				4	0.182	5.80E+04	4.76		
				5	0.174	2.22E+04	4.35		
				Mean	0.180	1.38E+05	4.91*	-2.69*	-1.49
				SEM	0.004	7.88E+04	0.21		
9	PT# 1284600 (NID-444) (PMB)	0.5 µg/dose IN, QD	25 h	1	0.214	2.92E+06	6.47		
				2	0.283	8.20E+05	5.91		
				3[d]	N/A	N/A	N/A		
				4	0.236	1.25E+09	9.10		
				5	0.190	5.50E+06	6.74		
				Mean	0.231	3.15E+08	7.05	-0.55	0.65
				SEM	0.020	3.12E+08	0.70		
10	PT# 1284597 (NID-441) (PMB - M13$^{\alpha LPS}$)	8 × 10^{10} virions/dose (0.528 µg/dose) IN, QD	25 h	1	0.138	1.80E+05	5.26		
				2	0.210	2.10E+04	4.32		
				3	0.215	5.70E+04	4.76		
				4	0.202	1.07E+04	4.03		
				5	0.219	1.47E+06	6.17		
				Mean	0.197	3.48E+05	4.91*	-2.69*	-1.49
				SEM	0.015	2.82E+05	0.38		
11	PT# 1284597 (NID-441) (PMB - M13$^{\alpha LPS}$)	4 × 10^{10} virions/dose (0.264 µg/dose) IN, QD	25 h	1	0.210	4.00E+04	4.60		
				2	0.163	5.40E+03	3.73		
				3	0.203	1.77E+07	7.25		
				4	0.173	1.37E+05	5.14		
				5	0.193	8.60E+04	4.93		
				Mean	0.188	3.59E+06	5.13*	-2.47*	-1.27
				SEM	0.009	3.53E+06	0.58		
12	PT# 1284597 (NID-441) (PMB - M13$^{\alpha LPS}$)	2 × 10^{10} virions/dose (0.132 µg/dose) IN, QD	25 h	1	0.185	8.10E+04	4.91		
				2	0.179	8.40E+07	7.92		
				3	0.213	1.12E+06	6.05		
				4	0.178	3.60E+06	6.56		
				5	0.188	7.20E+06	6.86		
				Mean	0.189	1.92E+07	6.46	-1.14	0.06
				SEM	0.006	1.62E+07	0.49		

δ: The $\log_{10}$ difference in the bacterial counts (CFU/lung) relative to the vehicle group (25 h vehicle counts).
Δ: The $\log_{10}$ difference in the bacterial counts (CFU/lung) relative to the baseline group (1 h initial counts).
(*): Indicates a significant difference ($p < 0.05$) compared to the vehicle control group as determined by one-way ANOVA and Dunnett's test.
(#): Indicates a significant difference ($p < 0.05$) compared to the baseline count as determined by one-way ANOVA and Dunnett's test.
(d): Indicates animals found dead before the scheduled sacrifice time point.
Concentration in the parenthesis is the equivalent PMB concentration.

Table S11. CFU/lung data for lung infection model in immunocompetent BALB/c mice. Bacterial burden in the total lung homogenate was determined by performing 10-fold serial dilutions and plating 0.1 mL of each onto MacConkey II agar plates. Colonies were counted after 18 – 24 h incubation. For each animal, the tissue weight and bacterial counts in each tissue homogenate dilution were recorded. For each tissue, the homogenate dilution that yielded the largest number of colonies, between 10 to 300 colonies per plate, was selected to calculate the bacterial counts per tissue (CFU/tissue) and the bacterial counts were tabulated. The raw colony count data of the homogenate dilutions were inspected for proportionality within the dilution series. The 10-fold serial dilutions are expected to show 10-fold reductions in counts. Disproportionate data, such as fewer counts in the undiluted homogenate samples compared to the diluted sample, would indicate inhibition of colony growth due to drug carryover from the lung tissue to the test plate. No aberrant titration data was observed.

Group	Test Articles	Animal No.	CFU/mL Homogenized Tissue
1	Vehicle (1xPBS)	1	430,000
		2	710,000
		3	2,900,000
		4	14,000,000
		5	210,000
		6	1,700,000
		7	1,500,000
		8	4,300,000
		9	400,000
2	$M13^{\alpha LPS}$ Phage 1E10 v	1	990,000
		2	580,000
		3	2,500,000
		4	1,280,000
		5	200,000
		6	900,000
		7	100,000
		8	220,000
		9	2,700,000
		10	2,700,000
3	WT-M13 Phage 1E10 v	1	13,0000
		2	19,000
		3	46,000
		4	490,000
		5	14,000
		6	440,000
		7	45,000
		8	1,300,000
		9	600
		10	5,300,000
4	PMB 0.5 µg	1	0
		2	0
		3	0
		4	0
		5	0
5	PMB 0.25 µg	1	0
		2	3,100
		3	0
		4	200
		5	0
6	PMB-$M13^{\alpha LPS}$ 2.5E9 v (PMB 0.017 µg)	1	500
		2	100
		3	0
		4	3,500
		5	0
7	PMB-$M13^{\alpha LPS}$ 1.25E9 v (PMB 0.008 µg)	1	0
		2	700
		3	29,000
		4	57,000
		5	100
		6	0
		7	0
		8	100
		9	260,000
		10	0
8	PMB-$M13^{\alpha LPS}$ 1.25E9 v (PMB 0.008 µg)	1	2,600,000
		2	1,100,000
		3	1,800,000
		4	140,000
		5	27,000
		6	100
		7	100
		8	0
		9	5,200
		10	1,000

Table S12. CFU/mL homogenized tissue data for *P. aeruginosa* corneal keratitis model. Bacterial burden in the total homogenate was determined by performing 10-fold serial dilutions and plating 0.01 mL of each onto LB agar plates. Colonies were counted after 18 – 24 h incubation and the CFU/mL of homogenized tissues were tabulated.

Animal #	Substance administered once per day for 7 days through IV tail vein injection in 100µL 1 x PBS buffer
16	1 x PBS
17	
18	
19	2.5 mg/kg body weight PMB
20	
21	
22	6.85 x 10^9 PMB-M13$^{\alpha LPS}$
23	
24	
25	1.37 x 10^10 PMB-M13$^{\alpha LPS}$
26	
27	
28	2.74 x 10^10 PMB-M13$^{\alpha LPS}$
29	
30	
31	No Injection of substance
32	
1	21 mg/kg body weight PMB
2	
3	
4	72 mg/kg body weight PMB
5	
6	
7	1.6 x 10^11 PMB-M13$^{\alpha LPS}$
8	
9	

Table S13. Animal numbering for histology during toxicity testing. Histological samples were numbered according to this table and the pathologist was blinded to the identity of each sample. Histology images are given on the DRYAD database. No histology images were available for animals number 4-6 due to death after single dose of test articles.

Supporting Text

Text S1. Recombinant M13 Phage Receptor-Binding Protein Constructs Verified by Sanger Sequencing

>Receptor Binding Protein HLL Construct
ATGAAATACCTATTGCCTACGGCGGCCGCTGGATTGTTATTACTCGCGGCCCAGCCGGCCAT
GGCTGAAGTCAAACTTGTCGAATCCGGTGGAGGGCTGGTACAGCCTGGTGGTTCCCTGAGTC
TGTCTTGTGCTGCGTCTGGTTTTACTTTTTCAGATTATTACATGACTTGGGTGCGTCAGGCCC
CCGGAAAAGCCCCAGAATGGCTCGCATTAATAAGAAATAAACGGAATGGTGATACAGCTGA
GTATTCCGCGAGTGTGAAAGGCCGCTTTACGATTTCCCGTGATTATTCACGGAGCATTCTGC
ATCTTCAAATGAATGCACTTCGCACTGAAGATTCGGCGACATACTATTGTGTGAGACAGGGT
CGCGGTTACACCTTAGATTATTGGGGCCAGGGTACCTCAGTGACCGTTTCTTCCGCCAAAAC
GACCGCTCCATCAGTGTATCCTTTAGCTCCGGTTTGTGGCGATACCACCGGGTCCTCGGTTAC
GCTGGGCTGCCTGGTAAAAGGTTACTTTCCGGAACCCGTTACACTGACATGGAACAGCGGCT
CCTTGTCAAGTGGTGTTCACACATTTCCCGCCGTGTTACAGTCTGGTCTCTACACGCTGAGTA
GCTCTGTCACCGTTACATCTTCTACATGGCCTAGCCAGTCTATTACCTGCAATGTAGCGCATC
CTGCGTCAAGCACGAAAGTTGATAAGAAAATCGAACCAGGTGGCGGGGGATCAGGAGGCG
GTGGGTCAGGCGGGGGAGGTTCGGACATACAAATGAATCAGAGCCCGAGCTCTTTGTCTGC
AAGCCTGGGCGATACCATCAGTATTACTTGTCGCGCCAGTCAGAATATTAATATCTGGTTAA
GCTGGTACCAACAGAAGCCTGGTAATGTGCCTAAACTTCTGATCTACAAAGCTAGCAATCTT
CATACAGGAGTACCGTCACGTTTCAGTGGCTCAGGAAGCGGCACCGACTTCACGTTGATTAT
CTCATCGCTCCAACCAGAGGATATTGCAACTTATTATTGCTTGCAGGGACAGAGCTATCCGC
GGACATTTGGGGGTGGTACCAAACTGGAGATTAAACGAGGCGACGCCGCACCGACTGTTTC
AATCTTCCCACCGTCGAGTGAACAATTAACTTCTGGGGGCGCGTCCGTCGTGTGTTTCCTCAA
CAACTTTTATCCCAAGGACATTAACGTAAAGTGGAAGATAGATGGGTCCGAACGTCAAAAC
GGAGTCTTGAACAGTTGGACTGACCAGGATTCAAAGGATAGCACGTATAGCATGAGTTCGA
CGCTGACACTGACGAAAGACGAGTATGAGCGACATAATTCGTATACCTGCGAAGCAACACA
CAAAACCTCTACTAGTCCGATAGTTAAAAGTTTTAACCGTGGCGGGGGCACCGGTGGTGGTT
CTGGTGGCGGCTCTGAGGGTGGCGGCTCTGAGGGTGGCGGTTCTGAGGGTGGCGGCTCTGA
GGGTGGCGGTTCCGGTGGCGGCTCCGGTTCCGGTGATTTTGATTATGAAAAAATGGCAAACG
CTAATAAGGGGGCTATGACCGAAAATGCCGATGAAAACGCGCTACAGTCTGACGCTAAAGG
CAAACTTGATTCTGTCGCTACTGATTACGGTGCTGCTATCGATGGTTTCATTGGTGACGTTTC
CGGCCTTGCTAATGGTAATGGTGCTACTGGTGATTTTGCTGGCTCTAATTCCCAAATGGCTCA
AGTCGGTGACGGTGATAATTCACCTTTAATGAATAATTTCCGTCAATATTTACCTTCTTTGCC
TCAGTCGGTTGAATGTCGCCCTTATGTCTTTGGCGCTGGTAAACCATATGAATTTTCTATTGA
TTGTGACAAAATAAACTTATTCCGTGGTGTCTTTGCGTTTCTTTTATATGTTGCCACCTTTATG
TATGTATTTTCGACGTTTGCTAACATACTGCGTAATAAGGAGTCTTAA

>Receptor Binding Protein LLH Construct
ATGAAATACCTATTGCCTACGGCGGCCGCTGGATTGTTATTACTCGCGGCCCAGCCGGCCAT
GGCTGATATTCAAATGAATCAGTCCCCTTCTTCCTTATCAGCCTCTTTGGGTGATACAATCAG
CATTACATGCCGAGCGTCACAGAATATCAACATCTGGCTGTCATGGTATCAACAAAAGCCGG
GTAATGTTCCAAAACTGTTGATCTACAAAGCTTCCAATCTTCATACTGGTGTTCCTTCGCGTT
TCAGTGGTAGCGGATCGGGTACTGATTTTACACTGATTATTTCGAGCCTGCAGCCTGAAGAC
ATTGCTACCTACTATTGCCTCCAAGGTCAGAGCTACCCGCGTACATTCGGCGGCGGCACTAA
ACTTGAGATAAAACGGGGCGACGCCGCCCCTACAGTATCAATCTTTCCGCCCTCCAGTGAAC
AGCTTACGAGTGGCGGTGCATCAGTTGTCTGTTTTTTGAACAACTTCTACCCAAAGGATATA

AACGTAAAGTGGAAGATTGACGGTTCAGAAAGACAGAATGGCGTGCTGAACTCTTGGACGG
ACCAGGATAGCAAAGACAGCACGTATAGTATGTCATCAACTTTAACACTCACTAAAGATGA
GTATGAAAGACATAACTCTTATACATGCGAAGCAACCCACAAGACCAGTACCTCTCCGATTG
TGAAAAGTTTCAATCGGGGAGGAGGGGGTAGTGGAGGTGGTGGCTCCGGAGGGGGCGGCTC
TGAAGTGAAGCTTGTAGAATCTGGTGGCGGCTTAGTGCAGCCCGGGGGTTCTCTCAGTCTCT
CCTGTGCAGCCTCAGGTTTTACGTTTAGCGATTATTATATGACATGGGTACGTCAGGCGCCA
GGGAAAGCCCCCGAATGGCTGGCATTGATTCGCAATAAACGAAATGGGGATACGGCCGAGT
ACAGTGCTTCAGTAAAAGGGCGGTTTACTATATCTCGTGATTATTCACGCAGCATTCTGCATC
TTCAAATGAATGCACTGCGCACTGAAGATAGCGCTACCTATTATTGTGTCCGCCAGGGACGT
GGGTACACCTTAGATTATTGGGGTCAAGGCACCTCCGTTACCGTCAGTTCCGCAAAAACGAC
CGCGCCGTCTGTGTATCCACTGGCGCCTGTTTGCGGTGACACGACGGGGAGTTCCGTGACCC
TGGGTTGTTTGGTTAAAGGATATTTTCCCGAGCCTGTCACGCTGACCTGGAATAGCGGCAGC
CTGTCGAGCGGGGTTCATACATTTCCAGCTGTCTTACAGTCTGGCCTGTACACATTATCGAGC
TCAGTTACTGTGACCTCGTCTACTTGGCCGTCGCAGTCCATAACGTGTAACGTGGCGCACCC
GGCGAGTAGCACCAAAGTTGATAAAAAAATCGAGCCGGGCGGAGGAACCGGTGGTGGTTCT
GGTGGCGGCTCTGAGGGTGGCGGCTCTGAGGGTGGCGGTTCTGAGGGTGGCGGCTCTGAGG
GTGGCGGTTCCGGTGGCGGCTCCGGTTCCGGTGATTTTGATTATGAAAAAATGGCAAACGCT
AATAAGGGGGCTATGACCGAAAATGCCGATGAAAACGCGCTACAGTCTGACGCTAAAGGCA
AACTTGATTCTGTCGCTACTGATTACGGTGCTGCTATCGATGGTTTCATTGGTGACGTTTCCG
GCCTTGCTAATGGTAATGGTGCTACTGGTGATTTTGCTGGCTCTAATTCCCAAATGGCTCAAG
TCGGTGACGGTGATAATTCACCTTTAATGAATAATTTCCGTCAATATTTACCTTCTTTGCCTC
AGTCGGTTGAATGTCGCCCTTATGTCTTTGGCGCTGGTAAACCATATGAATTTTCTATTGATT
GTGACAAAATAAACTTATTCCGTGGTGTCTTTGCGTTTCTTTTATATGTTGCCACCTTTATGTA
TGTATTTTCGACGTTTGCTAACATACTGCGTAATAAGGAGTCTTAA

Text S2. Matlab code for calculating mole percentage of amino acids in PMB-M13$^{\alpha LPS}$ given a varying number of PMB molecules (x) conjugated per g8p.

```
clear;clc;
mcpcopy =5; %minor capsid protein copies is 5 for each
M13otherproteins=repmat('EVKLVESGGGLVQPGGSLSLSCAASGFTFSDYYMTWVRQAPGKAPEWLALIRN
KRNGDTAEYSASVKGRFTISRDYSRSILHLQMNALRTEDSATYYCVRQGRGYTLDYWGQGTSVTVSSAKT
TAPSVYPLAPVCGDTTGSSVTLGCLVKGYFPEPVTLTWNSGSLSSGVHTFPAVLQSGLYTLSSSVTVTSSTW
PSQSITCNVAHPASSTKVDKKIEPGGGGSGGGGSGGGGSDIQMNQSPSSLSASLGDTISITCRASQNINIWLS
WYQQKPGNVPKLLIYKASNLHTGVPSRFSGSGSGTDFTLIISSLQPEDIATYYCLQGQSYPRTFGGGTKLEIK
RGDAAPTVSIFPPSSEQLTSGGASVVCFLNNFYPKDINVKWKIDGSERQNGVLNSWTDQDSKDSTYSMSST
LTLTKDEYERHNSYTCEATHKTSTSPIVKSFNRGGGTGGGSGGGSEGGGSEGGGSEGGGSEGGGSGGGSGS
GDFDYEKMANANKGAMTENADENALQSDAKGKLDSVATDYGAAIDGFIGDVSGLANGNATGDFAGSN
SQMAQVGDGDNSPLMNNFRQYLPSLPQSVECRPYVFGAGKPYEFSIDCDKINLFRGVFAFLLYVATFMYVF
STFANILRNKES',1,mcpcopy);%g3p sequence for anti-LPS phage
M13g8p=repmat('AEGDDPAKAAFNSLQASATEYIGYAWAMVVVIVGATIGIKLFKKFTSKAS',1,2700);%g8p
sequence for anti-LPS phage
G6P=repmat('PVLLGIPLLLRFLGFLLVTLFGYLLTFLKKGFGKIAIAISLFLALIIGLNSILVGYLSDISAQLPSDF
VQGVQLILPSNALPCFYVILSVKAAIFIFDVKQKIVSYLDWDK',1,mcpcopy);%g6p sequence for anti-LPS
phage
G7P=repmat('EQVADFDTIYQAMIQISVVLCFALGIIAGGQR',1,mcpcopy);%g7p sequence for anti-LPS phage
G9P=repmat('SVLVYSFASFVLGWCLRSGITYFTRLMETSS',1,mcpcopy);%g9p sequence for anti-LPS phage
PMBseq = 'TTFL';%protein sequence for PMB
PMBperg8p = x; %variable that could be changed, number of PMB conjugated per g8p
PMBtotal = repmat(PMBseq,1,2700*PMBperg8p);%all amino acid for 2700 copies of g8p conjugated with x PMB
per g8p
seq = append(M13otherproteins,M13g8p,G6P,G7P,G9P,PMBtotal);%total amino acids
allAA = sort('ARNDQEGHILKFPSTYVWCM');%counting and sorting all amino acids
counts = histc(seq, allAA);%counting each amino acid
sumi=sum(counts);
freq = counts/sumi;%calculating each amino acid frequency

for aa = allAA
    fprintf('%c: %d/%d (%.4f%%)\n', aa, counts(allAA==aa), sum(counts), freq(allAA==aa));
end
```
